# Comparison of pharmacogenomic information for drug approvals provided by the national regulatory agencies in Korea, Europe, Japan, and the United States

**DOI:** 10.3389/fphar.2023.1205624

**Published:** 2023-06-08

**Authors:** Mijin Lee, Ji Min Han, Jaeyeon Lee, Ju Young Oh, Jung Sun Kim, Hye Sun Gwak, Kyung Hee Choi

**Affiliations:** ^1^ College of Pharmacy, Sunchon National University, Suncheon, South Korea; ^2^ College of Pharmacy, Chungbuk National University, Cheongjusi, South Korea; ^3^ College of Pharmacy and Graduate School of Pharmaceutical Sciences, Ewha Womans University, Seoul, South Korea; ^4^ College of Pharmacy, Gachon University, Incheon, South Korea

**Keywords:** pharmacogenomic information, biomarkers, drug labeling, genetic testing, safety

## Abstract

Pharmacogenomics, which is defined as the study of changes in the properties of DNA and RNA associated with drug response, enables the prediction of the efficacy and adverse effects of drugs based on patients’ specific genetic mutations. For the safe and effective use of drugs, it is important that pharmacogenomic information is easily accessible to clinical experts and patients. Therefore, we examined the pharmacogenomic information provided on drug labels in Korea, Europe, Japan, and the United States (US). The selection of drugs that include pharmacogenomic information was based on the drug list that includes genetic information from the Korea Ministry of Food and Drug Safety (MFDS) and US Food and Drug Administration (FDA) websites. Drug labels were retrieved from the sites of MFDS, FDA, European Medicines Agency, and Japanese Pharmaceuticals and Medical Devices Agency. Drugs were classified as per the Anatomical Therapeutic Chemical code, and the biomarkers, labeling sections, and necessity of genetic tests were determined. In total, 348 drugs were selected from 380 drugs with available pharmacogenomic information in Korea and the US after applying the inclusion and exclusion criteria. Of these drugs, 137, 324, 169, and 126 were with pharmacogenomics information in Korea, the US, Europe, and Japan, respectively. The most commonly represented drug class was antineoplastic and immunomodulating agents. Regarding the classification as per the mentioned biomarkers, the cytochrome P450 enzyme was the most frequently mentioned information, and the targeted anticancer drugs most commonly required genetic biomarker testing. The reasons for differences in drug labeling information based on country include differences in mutant alleles according to ethnicity, frequencies at which drug lists are updated, and pharmacogenomics-related guidelines. Clinical experts must continuously strive to identify and report mutations that can explain drug efficacy or side effects for safe drug use.

## Introduction

Pharmacogenomics describes the variability of drug response according to a patient’s genetic information. Pharmacogenomics can facilitate the development of safe and effective drug therapy for individual patients by enabling appropriate drug selection and dose adjustment (Relling and Evans, 2015). Pharmacogenomics has developed rapidly over the past 20 years, consequently promoting the realization of “personalized medicine” in healthcare (Roncato et al., 2021).

Several initiatives have sought to apply pharmacogenomics to clinical practice. The Clinical Pharmacogenetics Implementation Consortium in the United States (US) and the Dutch Pharmacogenetics Working Group in Europe are the leading scientific consortia in the field of pharmacogenomics, and they are responsible for creating guidelines for the clinical application of genetic test results (Relling and Klein, 2011; Swen et al., 2011; Cecchin et al., 2017). Pharmacogenomic information is currently reflected in drug labels developed by regulatory agencies, and these data are used as biomarkers for drug treatment (FDA, 2021a; PMDA, 2021). Regulatory agencies in various countries, including the US and Europe, have supported the reflection of pharmacogenomic information via the publication of related guidance (Mehta et al., 2020). The European Medicines Agency has defined pharmacogenomics terms in “Position paper on terminology in pharmacogenetics” since 2002, and the US Food and Drug Administration (FDA) has defined pharmacogenomics and related terms in “Guidance for industry pharmacogenomic data submissions” since 2005. Guidelines have been prepared for subsequent application in clinical trials. In Japan, a guideline for collecting pharmacogenomic information has been published since 2005 (Ishiguro et al., 2008), and in Korea, a guideline for evaluating the eligibility of pharmacogenomics and applying the data to clinical trials was published in 2015 (MFDS, 2015a; MFDS, 2015b). Health Canada released the “Guidance document on submission of pharmacogenomic information” in 2007 as a guideline for providing pharmacogenomic data required for drug approval (Joly and Ramos-Paque, 2010).

The drug label reflection of pharmacogenomic information has made it easier to access the relevant information for application in clinical practice (Yoon et al., 2020). Studies examining whether drug labels reduce accidents related to adverse drug reactions and affect treatment outcomes are ongoing (Vredenburgh and Zackowitz, 2009; Wolf et al., 2016), and efforts to include pharmacogenomic information on drug labels are continuing (Kircik et al., 2017; Shekhani et al., 2020). In the field of oncology in particular, genetic testing for somatic mutation is already mandatory, and the relevant information is provided on the drug label. Meanwhile, clinical trial data are being added to drug labels for adverse drug reactions according to the genotype (Mehta et al., 2020). Drug labels reflecting pharmacogenomic information will facilitate the implementation of more appropriate and safer drug therapies in clinical settings (Yoon et al., 2020).

As each country has a different method for providing pharmacogenomic information and the standards indicated on the drug label are different, the content and inclusion of information may affect the safe use of drugs by patients. Therefore, by comparing pharmacogenomic information recorded by the US FDA, Korean Ministry of Food and Drug Safety (MFDS), European Medicines Agency, and Japanese Pharmaceuticals and Medical Devices Agency, we investigated the provision of pharmacogenomic information in each country or region as well as the information on drug labels according to the differences in guidelines.

## Materials and methods

### Data sources

To formulate the drug list for confirming the genomics-related drug label as per country, data from Korea and the US, which publish drug lists that include pharmacogenomic information on drug labels on regulatory agency websites, were used. Drug selection was based on the active ingredient by matching the Korean genetic information list uploaded on the Korean MFDS website and the “Table of Pharmacogenomic Biomarkers in Drug Labeling” drug list uploaded on the US FDA website as of December 2021 (MFDS, 2021a; FDA, 2022). Based on the drug list that included pharmacogenomics information, we reviewed whether the pharmacogenomics information of the drugs was included on drug labels formulated by ‘MFDS, FDA, European Medicines Agency’s Summary of Product Characteristics, and Japanese Pharmaceuticals and Medical Devices Agency (EMA, 2021; PMDA, 2021).

Drug labels were searched using by drug ingredient names via drug regulatory authorities in each country to extract information on Anatomical Therapeutic Chemical (ATC) codes, drug-related biomarkers, labeling sections, and the relevant pharmacogenomic contents.

Based on the drug lists of MFDS and FDA, the criteria for selecting drugs that included pharmacogenomic information in the relevant drug approvals in Korea, Europe, Japan, and the US were at least one of the categories as follows.

1) The drug was indicated only for patients with a specific genotype;
2) It was necessary to adjust the dose of the drug according to the genotype;
3) A genetic test for a specific biomarker was required before administration; and
4) Information such as contraindications or cautions regarding drug efficacy or safety was mentioned.

By contrast, drugs for which only genetic information in relation to excipients such as lactose intolerance were provided as well as those for which only the genotype was mentioned but dose adjustment was not required to improve efficacy or safety were excluded.

### Data analysis

To unify and classify the drug groups that were derived from each country based on the finally selected drug list, the groups were classified according to the ATC code using the World Health Organization/ATC defined daily dose index tool (WHOCC, 2021).

To evaluate the pharmacogenomic information on drug labels by country, biomarkers mentioned on drug labels in Korea, the US, Europe, and Japan were analyzed, and their number and properties were determined. For antineoplastic drugs, in particular, genetic testing for biomarkers is often essential; thus, the biomarkers and indications of the drug were confirmed to review the characteristics of drugs that require genetic testing.

There was a difference in drug label items among countries and regions, making it necessary to unify the approved items for comparison. The unification criteria for the permitted items were reclassified as follows.

1) Indications: contents for selecting appropriate patients who require genetic testing from pharmacogenomic information
2) Dosage and Administration; contents corresponding to dosing recommendations or dosage adjustments in the subgroups of patients according to genotype
3) Warnings and Precautions; contents of pharmacokinetic data, warnings, and precautions that affect the safety of the drug
4) Clinical Studies: pharmacogenomic information representing the clinical trial results for experts

## Results

**FIGURE 1:**
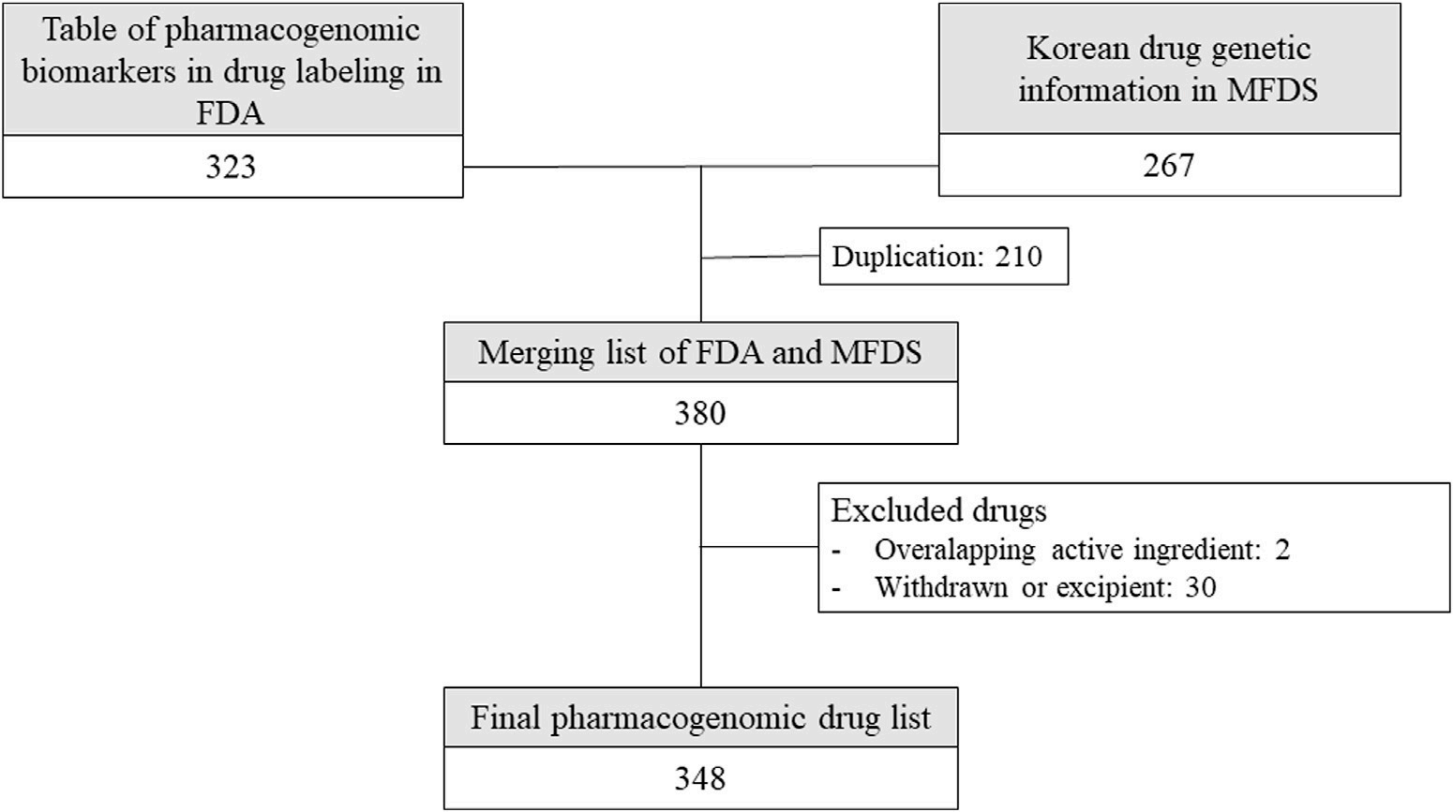
Drugs included in the analysis from drug lists provided by Korean and American regulators. FDA: United States Food and Drug Administration, MFDS: Korean Ministry of Food and Drug Safety.

Among the 380 drugs for which pharmacogenomic information was available in Korea and the US, 348 drugs were selected after excluding drugs with 2 overlapping ingredients; excipients; and drugs that are currently withdrawn or unauthorized in Korea, the US, Europe, or Japan (Figure 1).

Using the final drug list, the number of drugs for which drug genomic information was available was confirmed for each country. The proportion of drugs with available pharmacogenomic information was 59.1% (137 items) in Korea, 93.1% (324 items) in the US, 62.9% (171 items) in Europe, and 54.1% (126 items) in Japan (Table 1). The comparison of genetic information according to the drug list in each country is presented in Supplementary Table S1. The contents of drug labeling by each country are also provided (Supplementary Table S2–Supplementary Table S5).

**TABLE 1:** Number of drugs with pharmacogenomic information by country.

Classification	MFDS	FDA	EMA	PMDA
Authorized drugs	231	348	272	233
Drugs that included pharmacogenomic information	137	324	169	126
Drugs that did not include pharmacogenomic information	96	24	103	107
Withdrawn or Unauthorized drugs	117	0	76	115

MFDS: korean ministry of food and drug safety, FDA: united states food and drug administration, EMA: european medicines agency, PMDA: japanese pharmaceuticals and medical devices agency.

**FIGURE 2:**
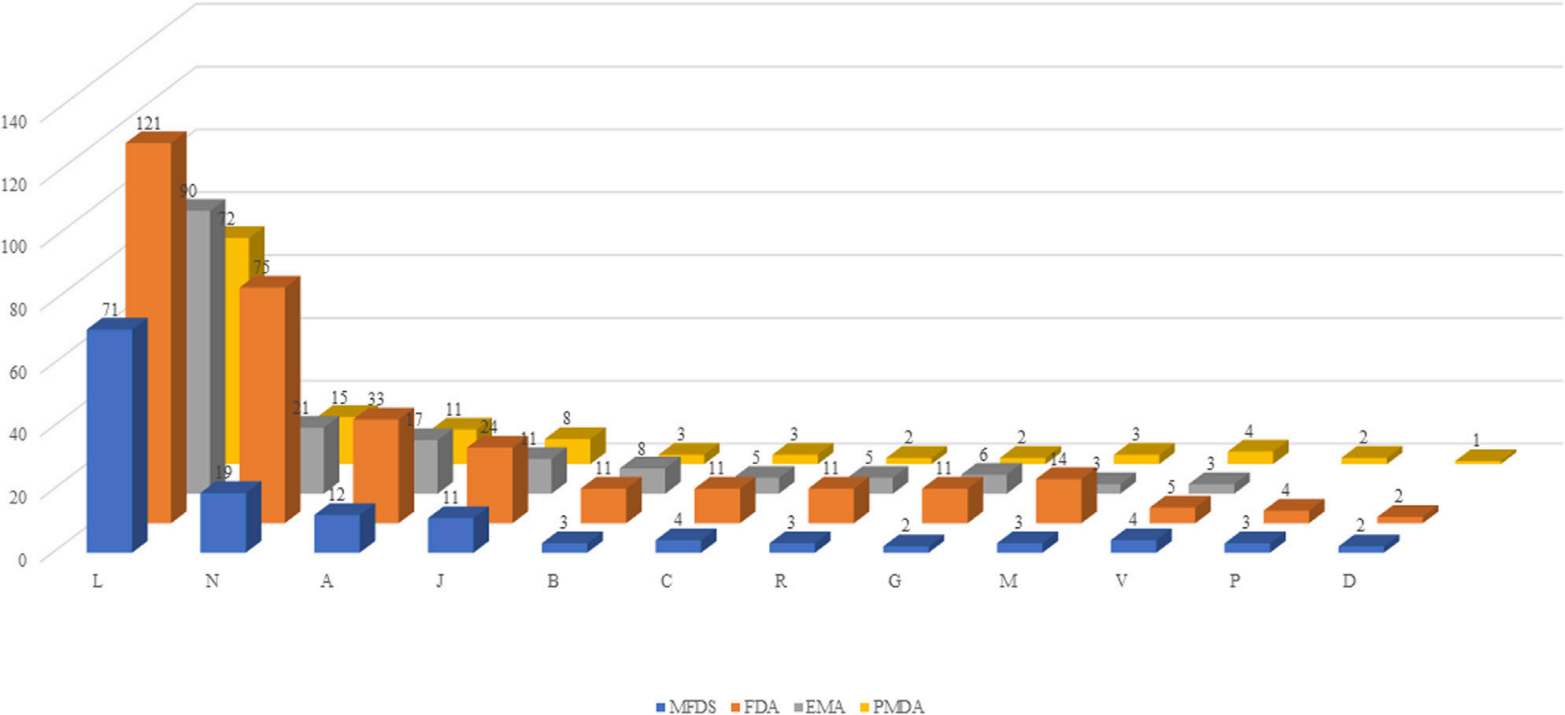
Classification by drug group. MFDS: Korean Ministry of Food and Drug Safety, FDA: United States Food and Drug Administration, EMA: European Medicines Agency, PMDA: Japanese Pharmaceuticals and Medical Devices Agency. A: Alimentary tract and metabolism, B-: Blood and blood forming organs, C: Cardiovascular system, D: Dermatologicals, G: Genitourinary system and sex hormones, J: Anti-infectives for systemic use, L: Antineoplastic and immunomodulating agents, M: Musculo-skeletal system, N: Nervous system, P: Antiparasitic products, insecticides and repellents, R: Respiratory system, S: Sensory organs, V: Various.

Using ATC codes, the drugs were classified into the following 12 drug groups: antineoplastic and immunomodulating drugs, nervous system drugs, alimentary tract and metabolism drugs, anti-infectives for systemic use, blood and blood-forming organ drugs, cardiovascular system drugs, respiratory system drugs, genitourinary system and sex hormone drugs, musculoskeletal system drugs. various, antiparasitic products, insecticides and repellents, and dermatologicals. Antineoplastic and immunomodulating agents accounted for the largest proportion of drugs in all four countries [Korea, 51.8% (71 items); US, 37.6% (121 items); Europe, 53.3% (90 items); and Japan, 57.1% (72 items)], whereas drugs acting on the nervous system accounted for the second largest proportion (Figure 2).

The proportions were confirmed by evaluating the biomarkers mentioned on the drug label. Regarding biomarkers, the most frequently mentioned pharmacogenomic information was the cytochrome P450 enzyme; it was mentioned for 102, 28, 39, and 25 times of the analyzed drugs in the US, Korea, Europe, and Japan, respectively. In the US, glucose-6-phosphate dehydrogenase was the second most common biomarker, accounting for 11.2% of drugs. In the remaining countries, biomarkers related to female cancer and non-small cell lung cancer were the second most frequently mentioned. Concerning the types of biomarkers mentioned, the US cited 63 species, followed by Europe (52 species), Japan (38 species), and Korea (40 species, Table 2).

**TABLE 2:** Classification and label contents according to biomarker.

Pharmacogenomic biomarkers	MFDS	FDA	EMA	PMDA	Note
AGXT		1	1		Alanine--glyoxylate and serine--pyruvate aminotransferase
ALDH5A1		1	1		Succinic semialdehyde dehydrogenase deficiency
ALK, EGFR, RAS, RAIIIF	18	28	22	21	NSCLC or mCRC
Androgen receptor			1		Prostate cancer
APOE		1			
AQP4 (aquaporin 4)	1	3	2	3	Nuromyelitis optica spectrum disorder
BCHE		2			Neuromuscular blocking agent
BCR/ABL1 (Philadelphia chromosome)	5	10	7	6	CML, ALL
BLyS (TNFSF13B, BAFF)			1		Systemic lupus erythematosus
BRAF	5	8	8	6	Metastatic melanoma
BRCA, ERBB2 (HER2), ESR, PGR (hormone receptor)	19	30	23	13	Breast cancer or ovarian cancer
CASR		1			Familial hypocalciuric hypercalcemia
CCR5	1		1	1	Human immunodeficiency virus
CD20, MS4A1 (CD20 antigen)	2	2	3	4	B-cell non-Hodgkin lymphomas
CD30	1	1	1	1	Hodgkin’s lymphoma
CD33		1	1	1	Acute myeloid leukemia
CD38			1		Multiple myeloma
CFTR		4	3		Cystic fibrosis
Chromosome 17p, 11q, 5q	2	5	3	1	
CIAS1, NLRP3		1	1		
CPOX, HMBS, PPOX gene		1			Acute hepatic porphyria
CPS, OTC, AS	1	1	1	1	Urea cycle disorders
CPT2	1	1			Carnitine Palmitoyltransferase II deficiency
CYB5R		5			Methemoglobinemia
CYP isoforms	28	102	39	25	
DMD gene		4			Duchenne muscular dystrophy
DPYD	2	2	1	2	
DRV-RAMs (darunavir resistance-associated mutations)	1				
Factor V Leiden, AT Ⅲ, factor Xa	1	6	4	1	
FGFR		3	1	1	Female cancer
FLT3	2	3	2	1	Acute myeloid leukemia
G6PD	17	37	5	11	Hemolytic anemia
GAA	1	1	1		Metabolic disorder
GALNS	1	1	1	1	Metabolic disorder
GLA	1	1	1	1	Metabolic disorder
Glucocerebrosidase, GBA gene			1	1	Metabolic disorder
HBB gene		3			
Hypercholesterolemia	1	1	1	3	Metabolic disorder
HGPRT	1	1	1	1	Metabolic disorder
HLA-A or B	5	9	2	4	Immune regulation
IDH1, IDH2		3	1		Metabolic disorder
IFNL3	1	12	5		Hepatitis C
IL-12, IL-23	1	1	1		Immune regulation
IL2RA (CD25 antigen)		1			T-cell lymphoma
INI genotypic resistance	1				Human immunodeficiency virus
JAK2				1	Myelofibrosis
LEP		1			
LMNA, ZMPSTE24		1			
MET gene	2	2		2	NSCLC
Microsatellite instability, mismatch repair	1	5	1	3	Various cancers
MYCN gene		1			Neuroblastoma
NAGS	1	1	1	1	Hyperammonemia
NAT2		1	1	1	Metabolic disorder
NECTIN4		1			Metastatic urothelial carcinoma
Nonspecific		18			
NTRK	2	2	2	2	Solid tumors
PCSK9		2			
PDGFRA		3	1	1	Soft tissue sarcoma
PD-L1	3	7	7	5	Cancer immunotherapy
P-gp (MDR1)			1		Efflux pump
PML/RARA	1	2	1	2	Acute promyelocytic leukemia
POLG	1	2			Alpers–Huttenlocher syndrome
PRF1, RAB27A, SH2D1A, STXBP2, STX11, UNC13D, XIAP	1				Hemophagocytic lymphohistiocytosis
PROC, PROS1, SERPINC1 (anti-thrombin III)		6			
P-selectin			1		Sickle cell disease
Reductase			1		Metabolic disorder
RET		3	3	2	Various cancers
Retinal phosphodiesterases			1		Retinitis pigmentosa
RYR1		1			Malignant hyperthermia
SLCO1B1, OCT2	2	3			Metabolic disorder
SMN1, SMN2	2	2	2	2	Spinal muscular atrophy
SSTR	1	1			Gastroenteropancreatic neuroendocrine tumors
TPMT, NUDT15	2	4	1	2	Cytopenias
TPP1	1	1	1	1	Ceroid lipofuscinosis type 2
TTR	1	3	3	2	Amyloidosis
UGT1A1	3	11	5	2	Metabolic disorder

MFDS:korean ministry of food and drug safety, FDA: united states food and drug administration, EMA: european medicines agency, PMDA: japanese pharmaceuticals and medical devices agency.

mCRC: metastatic colorectal cancer, NSCLC: non-small cell lung cancer, CML: chronic myelogenous leukemia. ALL: acute lymphoblastic leukemia.

ALK: anaplastic lymphoma receptor tyrosine kinase, EGFR: epidermal growth factor receptor, AQP4: aquaporin 4, BCHE: butyrylcholinesterase, BLyS: B lymphocyte stimulator, HER2: human epidermal growth factor receptor 2, ESR: estrogen receptor, PGR: progesterone receptor, CASR: calcium-sensing receptor, CCR5: C-C chemokine receptor type 5, CFTR: cystic fibrosis transmembrane conductance regulator, CPS: carbamoyl phosphate synthese, OTC: ornthine transcarbamylase, AS: argininosuccinate synthetase, CPT2: carnitine palmitoyltransferase 2, CYB5R: cytochrome b5 reductase, CYP: cytochrome P450, DPYD: dihydropyrimidine dehydrogenase, FGFR: fibroblast growth factor receptors, FLT3: fms-related tyrosine kinase 3, G6PD: glucose-6-phosphate dehydrogenase, GAA: alpha-glucosidase, GALNS: galactosamine (N-acetyl)-6-sulfatase, GLA: alpha-galactosidase A, HBB: beta-globin, HGPRT: hypoxanthine-guanine phosphoribosyltransferase, HLA: human leukocyte antigens, IDH: isocitrate dehydrogenase, IFNL3: interferon lambda 3, IL-12: interleukin 12, IL-2R, alpha: interleukin 2 receptor alpha, INI: integrase inhibitor, JAK2: Janus kinase 2, MDR1: multidrug resistance protein 1, NAGS: N-acetylglutamate synthase, NAT2: N-acetyltransferase 2, NTRK: neurotrophic tyrosine receptor kinase, PCSK9: proprotein convertase subtilisin/kexin type 9, PDGFRA: platelet-derived growth factor receptor A, P-gp: P-glycoprotein, POLG: DNA, polymerase gamma, RYR1: ryanodine receptor 1, SMN2: survival of motor neuron 2, SSTR: somatostatin receptors, TPMT: thiopurinemethyltransferase, TTR: transthyretin, NUDT15: nucleotide diphosphatase, UGT1A1: UDP, glucuronosyltransferase family 1 member A1.

Several antineoplastic drugs were divided into required and recommended groups according to whether genetic testing for biomarkers was placed in the “indication and usage” and “dosage and administration” sections or the “precautions for use” section on the drug label. We found that 92 drugs were classified with testing required and 31 drugs were testing recommendation. (Table 3; Table 4). Most drugs requiring genetic testing related to targeted anticancer agents, but biomarkers related to metabolism were recommended for assessment only in some patients in whom genetic testing was not essential.

**TABLE 3:** Anticancer drugs and required pharmacogenomic biomarker testing.

Drug	MFDS	FDA	EMA	PMDA	E/T
Abemaciclib	HR, HER2	HR, HER2	HR, HER2	HR, HER2	E
Ado-Trastuzumab Emtansine	HER2	HER2	HER2	HER2	E
Afatinib	EGFR	EGFR	EGFR	EGFR	E
Alectinib	ALK	ALK	ALK	ALK	E
Alpelisib	N/A	HER2, HR, PIK3CA	HER2, HR, PIK3CA	N/A	E
Amivantamab-vmjw	N/A	EGFR	N/A	N/A	E
Anastrozole	HR	HR	HR	None	E
Arsenic Trioxide	PML/RARA	PML/RARA	PML/RARA	PML/RARA^*^	E
Atezolizumab	EGFR, ALK	EGFR, ALK, PD-L1, BRAF	EGFR, ALK, PD-L1	EGFR, ALK, PD-L1, HER2	E
Avapritinib	N/A	PDGFRA	PDGFRA	N/A	E
Binimetinib	N/A	BRAF	BRAF	BRAF	E
Blinatumomab	None	BCR-ABL1^*^	BCR-ABL1, MRD, CD19	None	E
Bosutinib	N/A	BCR-ABL1	BCR-ABL1	BCR-ABL1^*^	E
Brentuximab Vedotin	CD30	TNFRSF8 (CD30)	CD30/TNFRSF8	CD30	E
Brigatinib	ALK	ALK	ALK	ALK	E
Capmatinib	MET	MET	None	MET	E
Casimersen	N/A	DMD	N/A	N/A	E
Ceritinib	ALK	ALK	ALK	ALK	E
Cetuximab	EGFR, RAS	EGFR, RAS	EGFR, RAS	EGFR, RAS	E/T
Cobimetinib	BRAF	BRAF	BRAF	N/A	E
Crizotinib	ALK, ROS1	ALK, ROS1	ALK, ROS1	ALK, ROS1	E
Dabrafenib	BRAF	BRAF, RAS	BRAF	BRAF, RAS	E/T
Dacomitinib	EGFR	EGFR	EGFR	EGFR, L858R	E
Dasatinib	BCR/ABL1	BCR-ABL1	BCR-ABL	BCR-ABL	E
Denileukin Diftitox	N/A	IL2RA (CD25 antigen)	None	None	E
Docetaxel	HER2	ESR, PGR (Hormone receptor) ^*^	HER2	None	E/T
Dostarlimab-gxly	N/A	Mismatch repair	Mismatch repair deficient (dMMR), Microsatellite instability-high(MSI-H)	N/A	E
Durvalumab	PD-L1^*^	PD-L1^*^	PD-L1	None	E
Enasidenib	N/A	IDH2	Isocitrate dehydrogenase 2 (IDH2)	N/A	E
Encorafenib	BRAF	BRAF, RAS	BRAF, RAS	BRAF	E/T
Entrectinib	NTRK, ROS1	NTRK, ROS1	NTRK, ROS1	NTRK, ROS1	E
Erdafitinib	N/A	FGFR	None	N/A	E/T
Erlotinib	EGFR	EGFR	EGFR, UGT1A1	EGFR	E/T
Everolimus	nonsteroidal-AIs, ESR, HER2	HER2, ESR	HER2	HER2, ESR^*^	E
Exemestane	ESR	ESR, PGR (Hormone receptor)	None	None	E
Fam-Trastuzumab Deruxtecan-nxki	N/A	HER2	HER2	HER2	E
Fulvestrant	HER2, HR	HER2, ESR, PGR (Hormone receptor)	HR, HER2	HER2, ESR^*^	E
Gefitinib	EGFR	EGFR	EGFR	EGFR	E
Gemtuzumab Ozogamicin	N/A	CD33	CD33	CD33	E
Gilteritinib	FLT3	FLT3	FLT3	FLT3	E
Goserelin	ESR	ESR, PGR (Hormone receptor)	None	None	E
Ibrutinib	None	Chromosome 17p	Chromosome 17p^*^	None	E
Imatinib	BCR-ABL1, KIT, PDGFR	BCR-ABL1, KIT, PDGFRB, FIP1L1-PDGFRA	BCR-ABL1, KIT, PDGFR, FIP1L1-PDGFR	BCR-ABL1, KIT, FIP1L1-PDGFRα	E
Infigratinib	N/A	FGFR2	N/A	N/A	E
Ipilimumab	None	MSI, Mismatch repair, PD-L1, ALK, EGFR	PD-L1, ALK, EGFR	PD-L1, EGFR, ALK, MSI, Mismatch repair	E
Ivosidenib	N/A	IDH1	None	N/A	E
Larotrectinib	NTRK	NTRK	NTRK	NTRK	E
Lenalidomide	Chromosome 5q	Chromosome 5q	Chromosome 5q	Chromosome 5q	E
Lenvatinib	MSI, Mismatch repair	MSI, Mismatch repair	None	None	E
Letrozole	ESR, PGR	ESR, PGR (Hormone receptor)	ESR, PGR^*^	None	E
Lonafarnib	N/A	LMNA, ZMPSTE24	N/A	N/A	E
Lorlatinib	N/A	ALK, ROS1	ALK	ALK, ROS1	E
Margetuximab-cmkb	N/A	HER2	N/A	N/A	E
Midostaurin	FLT3	FLT3	FLT3	N/A	E
Neratinib	HER2	HER2	HER2	N/A	E
Nilotinib	BCR-ABL1	BCR-ABL1	BCR-ABL1	BCR-ABL1^*^	E
Niraparib	BRCA, HRD	BRCA, HRD	BRCA^*^	BRCA, HRD	E
Nivolumab	EGFR, ALK, PD-L1	PD-L1, MSI, Mismatch repair, EGFR, ALK	PD-L1, EGFR, ALK	MSI, EGFR, ALK, PD-L1, Mismatch repair	E
Olaparib	BRCA	BRCA, HER2, ESR, PGR (Hormone receptor), HRD, Homologous recombination repair	BRCA, HER2, genomic instability	BRCA, HER2, Homologous recombination repair	E
Osimertinib	EGFR	EGFR	EGFR	EGFR	E
Palbociclib	HER2, HR	ESR (Hormone receptor), HER2	HER2	Hormone receptor (HR), HER2	E
Panitumumab	N/A	RAS	RAS	RAS	E
Pembrolizumab	PD-L1, EGFR, ALK	BRAF, PD-L1, MSI, Mismatch repair, EGFR, ALK^*^	BRAF, PD-L1, EGFR, ALK	PD-L1, ALK, EGFR, MSI	E
Pemigatinib	N/A	FGFR2	FGFR2	FGFR2	E
Pertuzumab	HER2	HER2	HER2	HER2	E
Ponatinib	BCR-ABL, T315I	BCR-ABL	BCR-ABL	BCR-ABL	E
Ramucirumab	EGFR, ALK	EGFR	EGFR, RAS	EGFR	E
Regorafenib	RAS	RAS	RAS	RAS^*^	E/T
Ribociclib	HR, HER2	ESR, PGR (Hormone receptor), HER2	HR, HER2	N/A	E
Rituximab	CD20	CD20	CD20	CD20	E
Rucaparib	N/A	BRCA	BRCA	N/A	E
Selpercatinib	N/A	RET	RET	RET	E
Sotorasib	N/A	KRAS	N/A	N/A	E
Talazoparib	BRCA, HER2	BRCA, HER2	BRCA, HER2	N/A	E
Tamoxifen	None	ESR, PGR (Hormone receptor)	None	None	E
Tepotinib	MET	MET	N/A	MET	E
Tipiracil and Trifluridine	HER2, RAS	HER2, RAS	HER2, RAS^*^	HER2, RAS^*^	E/T
Toremifene	None	ESR (Hormone receptor)	Estrogen receptor	Estrogen receptor (ER)	E
Trametinib	BRAF, G6PD	BRAF, G6PD, RAS	BRAF	BRAF	E/T
Trastuzumab	HER2	HER2	HER2	HER2	E
Tretinoin	None	PML/RARA	None	PML/RARA	E
Tucatinib	N/A	HER2	None	N/A	E
Vemurafenib	BRAF	BRAF, RAS	BRAF, RAS	BRAF, RAS	E/T
Vincristine	None	BCR-ABL1	None	None	E

E: efficacy, T: toxicity, N/A: not authorized, None: No content on the label.

^*^ Not required test.

**TABLE 4:** Anticancer drugs and recommended pharmacogenomic biomarker testing.

Drug	MFDS	FDA	EMA	PMDA	E/T
Axitinib	CYP3A4/5	None	None	None	T
Azathioprine	TPMT, NUDT15	TPMT, NUDT15	None	TPMT, NUDT15	T
Belimumab	None	None	BLyS (TNFSF13B, BAFF)	None	E
Belinostat	N/A	UGT1A1	None	N/A	T
Capecitabine	DPYD	DPYD	DPYD	DPYD	T
Cisplatin	None	TPMT	None	None	E
Enzalutamide	CYP2C8	None	Androgen receptor	None	E/T
Fluorouracil	DPYD	DPYD	None	DPYD	T
Flutamide	N/A	G6PD	None	None	T
Irinotecan	UGT1A1	UGT1A1	UGT1A1	UGT1A1	T
Mercaptopurine	TPMT	TPMT, NUDT15	TPMT, NUDT15	NUDT15	T
Pazopanib	UGT1A1	UGT1A1, HLA-B	HLA-B	None	T
Peginterferon Alfa-2b	None	IFNL3 (IL28B)	None	None	E
Pralsetinib	N/A	CCDC6-RET, KIF5B-RET, RET	N/A	N/A	E
Ruxolitinib	None	None	None	JAK2	E
Sacituzumab Govitecan-hziy	N/A	UGT1A1	None	N/A	T
Thioguanine	None	TPMT, NUDT15	None	N/A	T
Vandetanib	CYP3A4	None	RET	RET	E/T

E: efficacy, T: toxicity, N/A: not authorized, None: No content on the label.

By checking drug label items that included pharmacogenomic information, we found that in Korea, pharmacogenomic information was primarily included for indications (49.6%) and warnings and precautions (41.6%), whereas in the US, pharmacogenomic information was most commonly included for warnings and precautions (50.9%). In Europe (53.3%) and Japan (55.6%), pharmacogenomic information was most frequently included for indications (Table 5).

**TABLE 5:** Drug labeling section, including drug genomic information for each country.

Labeling section	MFDS	FDA	EMA	PMDA
Indications	68	105	90	70
Dosage and Administration	11	26	12	8
Warnings and Precautions	57	165	43	45
Clinical Studies	1	28	24	3

MFDS: korean ministry of food and drug safety, FDA: united states food and drug administration, EMA: european medicines agency, PMDA: japanese pharmaceuticals and medical devices agency.

## Discussion

Pharmacogenomics, which is a field of precision medicine that aims to develop preventative and treatment strategies considering individual genetic diversity, can be helpful for predicting the safety and efficacy of drugs and ensuring that drugs are prescribed according to patients’ genetic characteristics (Collins and Varmus, 2015). Because of the challenge regarding the clinical utility of routine genetic testing, its impact on drug labeling and its clinical application remains limited (Gillis and Innocenti, 2014). In addition, if the information reflected on the drug label differs among countries, the pharmacogenomic information applicable to the patient will be further reduced. Therefore, comparing information on drug labels among different countries to recognize differences is important for improving access to pharmacogenomic information. Although drug regulatory agencies in different countries should include relevant information on the drug label, the content of the label differed among the countries in some case. The same biomarkers were mentioned by all countries only in select cases. The following were the reasons for the differences in label information: differences in the frequency of mutant alleles because of ethnic differences, country-specific pharmacogenomic guidelines, and frequency of pharmacogenomic drug list updates (Yasuda et al., 2008; Imatoh et al., 2018).

An example of ethnic differences can be observed for carbamazepine. Carbamazepine-induced Stevens–Johnson syndrome and toxic epidermal necrolysis are highly correlated with HLA-B*1502, the allele frequency of which varies according to ethnicity, being most common in some Asian populations (Hung et al., 2006). In Korea and the US, the carbamazepine label stated that testing for HLA-B*1502 might be necessary (FDA, 2021b; MFDS, 2021b). Meanwhile, in Europe, where the probability of HLA-B*1502 presence is low, the carbamazepine label did not include biomarker information. Another example of a drug requiring an ethnicity-based treatment strategy is the antihypertensive combination regimen of isosorbide dinitrate and hydralazine, which has been proven to be effective only in African Americans in clinical trials and was approved by FDA for use only in this population (Taylor et al., 2004). For this reason, if the target of the drug is the biomarker testing is required. When the need for testing was restricted to specific patients or ethnic populations or related to safety in a small number of patients, the test was recommended or only required in patients at high risk.

Another factor that causes differences in the genetic information on the drug label among countries is the difference in the guidelines for genetic information. All four reviewed countries or regions follow the International Conference on Harmonization of Technical Requirements for Registration of Pharmaceuticals for Human Use guidelines for the definitions of terms and regulatory data for the qualification of genomic biomarkers; however, recommendations for drug labeling for genetic information are based on country-specific guidelines. The US recommends a labeling section for pharmacogenetic information in “Guidance for Industry Clinical Pharmacogenomics: Premarket Evaluation in Early-Phase Clinical Studies and Recommendations for Labeling” (published in January 2013), and Korea published recommendations for labeling pharmacogenomics in 2015. When appropriate patient selection or genome testing is required, the recommendations are described in the “indication and usage” section, recommendations for patient subgroups according to the genotype are provided in the “dosage and administration” section, and other matters related to safety are placed in the “precautions for use” section (FDA, 2012; MFDS, 2015). In Europe, the results of the pharmacovigilance assessments of drugs, that include drug genomic information, were included in the appropriate treatment recommendations for labeling in 2013 (EMA, 2015). Since 2005, Japan has required data from genetic and many other tests in addition to the guidelines for the use of pharmacogenetics in clinical trials in terms of efficacy, effect, usage, dosage, or use precautions for drug approval, and since 2014, drugs that require genetic testing for specific biomarkers have been regulated (PMDA, 2005; PMDA, 2022). The genetic information of a new drug may be provided or missed depending on the timing of the drug list updates that include genetic information. In the US, it was confirmed that the drug list that includes pharmacogenomic information is at least updated annually, whereas in Korea, the list has not been updated since 2018. Meanwhile, a drug list that includes pharmacogenomic information is not provided in Europe and Japan.

Among the drugs for which pharmacogenomic information is mentioned, antineoplastic agents most commonly required a biomarker test. The importance of pharmacogenomic information in cancer treatment is well known because many anticancer drugs have narrow therapeutic ranges, and patients’ genetic background can have an effect on the pharmacokinetics of anticancer drugs (Roncato et al., 2021). In addition, it has been revealed that targeted agents can be used as therapeutic options in addition to commonly used cytotoxic agents based on specific genetic mutations in various cancers including breast cancer, lung cancer, colorectal cancer, and melanoma (Flaherty et al., 2010; Zhou et al., 2011; Yang et al., 2012; Roengvoraphoj et al., 2013). In this case, genetic testing is usually required or recommended to predict efficacy prior to the initiation of targeted therapy.

As a study limitation, it is possible that individual judgment was involved in the process of matching the pharmacogenomic list and collecting the pharmacogenomic information. However, to exclude this possibility as far as possible, the drug selection and exclusion criteria were set and then the information was collected and analyzed. It was difficult to compare information among the regions because the expression method and language of drug label items differed among the countries. In the process of unifying the information, we tried to minimize deviations by setting classification criteria.

Among the items of the drug label, the information on the pharmacogenomics mentioned in the indications is important for defining the patient group; thus, genetic testing is mandatory. When genomic information was included for items other than indications, however, only some drug lists actively recommended genetic testing. Targeted anticancer drugs were developed using genetic testing in the preclinical stage, and genetic testing was applied in clinical trials to collect information. By contrast, as side effects were documented and research progressed on drugs such as warfarin and abacavir after they were marketed, the relationship with the genome was confirmed and genetic tests were developed and approved (Cohen, 2012). Although side effects related to pharmacogenomics have been reported in each country, genetic testing is not actively recommended on drug labels. Hence, it is vital to secure evidence on the clinical usefulness of drug genomic information that can be used for genetic testing and follow-up management for already approved drugs.

Because pharmacogenomics information can ensure the safe use of drugs by patients, it is important to quickly and accurately provide this information on the drug label in a manner that is easily accessible to the patient and medical staff. Therefore, healthcare providers, including clinicians and pharmacists, require continuously updated information regarding mutations that can explain efficacy or adverse effects in patients during treatment via continuous pharmacogenomic studies and drug label updates. In addition, we believe that each country should both provide latest information via the rapid updates of genetic information and harmonize drug labeling guidelines related to pharmacogenomics with other countries.

## Conclusion

The reasons for differences in drug label information among countries include differences in mutant alleles according to ethnicity, update frequency of drug lists, and pharmacogenomics-related guidelines. To provide a wide range of information to patients and medical staff, it is important to harmonize the standards of drug labels among countries. Furthermore, for the safe use of drugs in patients, clinical experts must continuously strive to identify and report mutations that can explain drug efficacy or side effects in patients.

## Data Availability

The original contributions presented in the study are included in the article/Supplementary Material, further inquiries can be directed to the corresponding authors.
