# Supplementary material for: Comparison of pharmacogenomic information for drug approvals provided by the national regulatory agencies in Korea, Europe, Japan, and the United States
Source: Front Pharmacol. 2023 Jun 8;14:1205624. doi: 10.3389/fphar.2023.1205624 (PMC10285385; doi:10.3389/fphar.2023.1205624)
Supplement: Supplementary file 1 [file Table1.DOCX]

**S1 Table.** Pharmacogenomic drug information comparison between countries

| **Drug** | **MFDS** | **FDA** | **EMA** | **PMDA** |
| --- | --- | --- | --- | --- |
| Abacavir | O | O | O | O |
| Abemaciclib | O | O | O | O |
| Ado-Trastuzumab Emtansine | O | O | O | O |
| Aducanumab-avwa | - | O | - | - |
| Afatinib | O | O | O | O |
| Alectinib | O | O | O | O |
| Alglucosidase Alfa | O | O | O | X |
| Alirocumab | X | X | O | X |
| Aliskiren | - | X | O | X |
| Allopurinol | O | O | X | O |
| Alpelisib | - | O | O | - |
| Amifampridine | - | O | O | - |
| Amitriptyline | X | O | O | X |
| Amivantamab-vmjw | - | O | - | - |
| Amoxapine(loxapine) | X | O | X | X |
| Amphetamine | - | O | - | - |
| Anakinra | - | O | O | - |
| Anastrozole | O | O | O | X |
| Arformoterol | - | O | - | - |
| Aripiprazole | X | O | O | X |
| Arsenic Trioxide | O | O | O | O |
| Articaine and Epinephrine | X | O | X | - |
| Ascorbic Acid, PEG-3350, Potassium Chloride, Sodium Ascorbate, Sodium Chloride, and Sodium Sulfate | X | O | X | - |
| Atazanavir | X | X | O | O |
| Atezolizumab | O | O | O | O |
| Atomoxetine | O | O | X | O |
| Atorvastatin | X | X | X | O |
| Avapritinib | - | O | O | - |
| Avatrombopag | - | O | O | - |
| Avelumab | X | O | O | O |
| Axitinib | O | X | X | X |
| Azathioprine | O | O | X | O |
| Belimumab | X | X | O | X |
| Belinostat | - | O | X | - |
| Binimetinib | - | O | O | O |
| Blinatumomab | X | O | O | X |
| Boceprevir | - | O | - | - |
| Bosutinib | - | O | O | O |
| Brentuximab Vedotin | O | O | O | O |
| Brexpiprazole | O | O | O | O |
| Brigatinib | O | O | O | O |
| Brivaracetam | X | O | O | - |
| Bupivacaine | X | O | X | X |
| Bupropion | X | O | X | - |
| Busulfan | X | O | X | X |
| Cabotegravir and Rilpivirine | - | O | - | - |
| Cabozantinib | X | O | O | X |
| Capecitabine | O | O | O | O |
| Capmatinib | O | O | X | O |
| Carbamazepine | O | O | X | O |
| Carglumic Acid | O | O | O | O |
| Cariprazine | - | O | X | - |
| Carisoprodol | - | O | X | - |
| Carvedilol | O | O | X | X |
| Casimersen | - | O | - | - |
| Ceftriaxone | X | O | X | X |
| Celecoxib | X | O | X | O |
| Cemiplimab-rwlc | - | O | O | - |
| Ceritinib | O | O | O | O |
| Cerliponase Alfa | O | O | O | O |
| Cetuximab | O | O | O | O |
| Cevimeline | - | O | - | X |
| Chloroprocaine | - | O | X | - |
| Chloroquine | O | O | X | - |
| Chlorpropamide | - | O | - | X |
| Cholic acid | - | O | O | - |
| Cisplatin | X | O | X | X |
| Citalopram | X | O | X | - |
| Clobazam | O | O | X | X |
| Clomipramine | X | O | - | X |
| Clopidogrel | O | O | O | O |
| Clozapine | X | O | X | X |
| Cobimetinib | O | O | O | - |
| Codeine | O | O | O | O |
| Crizanlizumab-tmca | - | O | O | - |
| Crizotinib | O | O | O | O |
| Dabrafenib | O | O | O | O |
| Daclatasvir | X | O | - | X |
| Dacomitinib | O | O | O | O |
| Dapsone | O | O | - | O |
| Darifenacin | - | O | O | - |
| Darunavir | O | X | X | X |
| Dasabuvir, Ombitasvir, Paritaprevir, and Ritonavir | - | O | X | - |
| Dasatinib | O | O | O | O |
| Denileukin Diftitox | - | O | X | X |
| Desflurane | X | O | X | X |
| Desipramine | - | O | - | - |
| Desvenlafaxine | X | O | X | - |
| Deutetrabenazine | O | O | - | - |
| Dexlansoprazole | X | O | X | - |
| Dextromethorphan and Quinidine | - | O | - | - |
| Diazepam | X | O | - | X |
| Dinutuximab | - | O | - | X |
| Divalproex sodium | O | O | - | - |
| Docetaxel | O | O | O | X |
| Dolutegravir | O | O | O | X |
| Donepezil | X | O | X | X |
| Dostarlimab-gxly | - | O | O | - |
| Doxepin | X | O | - | - |
| Dronabinol | - | O | X | - |
| Drospirenone and Ethinyl Estradiol | O | O | X | X |
| Duloxetine | X | O | O | X |
| Durvalumab | O | O | O | X |
| Duvelisib | - | O | X | - |
| Eculizumab | X | O | O | O |
| Efavirenz | O | O | O | O |
| Elagolix | - | O | X | - |
| Elbasvir and Grazoprevir | O | O | O | - |
| Elexacaftor, Ivacaftor, and Tezacaftor | - | O | O | - |
| Eliglustat | O | O | O | O |
| Elosulfase | O | O | O | O |
| Eltrombopag | O | O | O | O |
| Emapalumab-lzsg | - | O | X | - |
| Enasidenib | - | O | O | - |
| Encorafenib | O | O | O | O |
| Enfortumab Vedotin-ejfv | - | O | X | - |
| Entrectinib | O | O | O | O |
| Enzalutamide | O | X | O | X |
| Erdafitinib | - | O | X | - |
| Eribulin | X | O | O | X |
| Erlotinib | O | O | O | O |
| Erythromycin and Sulfisoxazole | - | O | - | - |
| Escitalopram | O | O | X | O |
| Esomeprazole | X | O | O | O |
| Estradiol and Progesterone | X | O | X | - |
| Eteplirsen | - | O | - | - |
| Ethinyl estradiol and Norelgestromin | - | X | O | - |
| Everolimus | O | O | O | O |
| Evinacumab-dgnb | - | O | O | - |
| Evolocumab | O | X | O | O |
| Exemestane | O | O | X | X |
| Fampridine | O | X | X | - |
| Fam-Trastuzumab Deruxtecan-nxki | - | O | O | O |
| Fesoterodine | X | O | O | O |
| Flibanserin | - | O | X | - |
| Fluorouracil | O | O | X | O |
| Fluoxetine | X | O | X | - |
| Flurbiprofen | X | O | X | X |
| Flutamide | - | O | X | X |
| Fluvoxamine | X | O | X | X |
| Formoterol | X | O | - | X |
| Fosphenytoin | O | O | - | X |
| Fulvestrant | O | O | O | O |
| Galantamine | O | O | X | X |
| Gefitinib | O | O | O | O |
| Gemtuzumab Ozogamicin | - | O | O | O |
| Gilteritinib | O | O | O | O |
| Givosiran | - | O | X | X |
| Glimepiride | O | O | X | X |
| Glipizide | X | O | X | - |
| Glyburide (Glibenclamide) | O | O | O | X |
| Golodirsen | - | O | - | - |
| Goserelin | O | O | X | X |
| Hydralazine | X | O | - | X |
| Hydroxychloroquine | O | O | X | O |
| Ibritumomab | O | X | O | O |
| Ibrutinib | X | O | O | X |
| Iloperidone | - | O | - | - |
| Imatinib | O | O | O | O |
| Imipramine | X | O | - | X |
| Indacaterol | O | O | X | O |
| Inebilizumab-cdon | O | O | X | O |
| Infigratinib | - | O | - | - |
| Inotersen | - | O | O | - |
| Inotuzumab Ozogamicin | O | O | O | O |
| Ipilimumab | X | O | O | O |
| Irinotecan | O | O | O | O |
| Isatuximab- irfc | - | O | O | X |
| Isoflurane | X | O | X | X |
| Isoniazid | X | X | X | O |
| Isoniazid, Pyrazinamide, and Rifampin | X | O | - | - |
| Isosorbide Dinitrate | X | O | - | X |
| Isosorbide Mononitrate | X | O | - | X |
| Ivacaftor | - | O | O | - |
| Ivacaftor and Lumacaftor | - | O | O | - |
| Ivacaftor and Tezacaftor | - | O | O | - |
| Ivosidenib | - | O | X | - |
| Ixabepilone | - | O | - | - |
| Lacosamide | X | O | X | O |
| Lansoprazole | X | O | O | X |
| Lapatinib | O | O | O | O |
| Larotrectinib | O | O | O | O |
| Ledipasvir and Sofosbuvir | X | O | O | X |
| Lenalidomide | O | O | O | O |
| Lenvatinib | O | O | X | X |
| Lesinurad | - | O | - | - |
| Letrozole | O | O | O | O |
| Lidocaine and Prilocaine | O | O | O | O |
| Lidocaine and Tetracaine | - | O | X | - |
| Lofexidine | - | O | - | - |
| Lomitapide | - | X | O | O |
| Lonafarnib | - | O | - | - |
| Lorlatinib | - | O | O | O |
| Lumasiran | - | O | O | - |
| Luspatercept–aamt | - | O | X | - |
| Lusutrombopag | - | O | X | X |
| Lutetium Dotatate Lu-177 | O | O | X | X |
| Mafenide | O | O | - | - |
| Maraviroc | O | X | O | O |
| Margetuximab-cmkb | - | O | - | - |
| Meclizine | X | O | - | - |
| Meloxicam | X | O | X | X |
| Mepivacaine | X | O | X | X |
| Mercaptopurine | O | O | O | O |
| Methylene Blue | O | O | O | O |
| Metoclopramide | O | O | X | X |
| Metoprolol | X | O | - | X |
| Metreleptin | - | O | X | X |
| Midostaurin | O | O | O | - |
| Migalastat | O | O | O | O |
| Mirabegron | X | O | X | X |
| Mivacurium | - | O | X | - |
| Modafinil | O | O | X | X |
| Moviprep | - | X | - | O |
| Mycophenolic Acid | O | O | O | O |
| Nalidixic Acid | X | O | X | O |
| Nebivolol | X | O | X | - |
| Nefazodone | - | O | - | - |
| Neratinib | O | O | O | - |
| Nilotinib | O | O | O | O |
| Niraparib | O | O | O | O |
| Nitrofurantoin | X | O | - | - |
| Nivolumab | O | O | O | O |
| Norfloxacin | O | X | X | X |
| Nortriptyline | X | O | X | X |
| Nusinersen | O | O | O | O |
| Obinutuzumab | X | O | O | O |
| Ofatumumab | - | X | X | O |
| Olaparib | O | O | O | O |
| Olaratumab | - | O | - | - |
| Oliceridine | - | O | - | - |
| Omacetaxine | - | O | - | - |
| Ombitasvir, Paritaprevir, and Ritonavir | - | O | X | - |
| Omeprazole | X | O | O | O |
| Ondansetron | X | O | X | X |
| Osimertinib | O | O | O | O |
| Ospemifene | - | O | X | - |
| Oxcarbazepine | O | O | X | X |
| Oxymetazoline and Tetracaine | - | O | X | - |
| Palbociclib | O | O | O | O |
| Paliperidone | X | O | X | X |
| Palonosetron | X | O | X | O |
| Panitumumab | - | O | O | O |
| Pantoprazole | X | O | X | - |
| Parathyroid Hormone | - | O | X | - |
| Paroxetine | X | O | X | O |
| Patisiran | - | O | O | O |
| Pazopanib | O | O | O | X |
| Peginterferon Alfa-2b | X | O | X | X |
| Pegloticase | - | O | O | - |
| Pembrolizumab | O | O | O | O |
| Pemigatinib | - | O | O | O |
| Perphenazine | X | O | X | X |
| Pertuzumab | O | O | O | O |
| Phenytoin | X | O | - | X |
| Pimozide | O | O | - | X |
| Piroxicam | X | O | X | X |
| Pitolisant | O | O | O | - |
| Ponatinib | O | O | O | O |
| Pralsetinib | - | O | - | - |
| Prasugrel | X | O | X | X |
| Primaquine | O | O | - | O |
| Probenecid | - | O | - | X |
| Procainamide | - | O | - | X |
| Propafenone | X | O | X | X |
| Propranolol | X | O | X | X |
| Protriptyline | - | O | - | - |
| Quinidine | - | O | - | X |
| Quinine Sulfate | - | O | X | X |
| Rabeprazole | X | O | X | O |
| Raloxifene | X | O | O | X |
| Raltegravir | X | O | X | X |
| Ramucirumab | O | O | O | O |
| Ranolazine | O | X | O | - |
| Rasburicase | O | O | O | O |
| Regorafenib | O | O | O | O |
| Ribociclib | O | O | O | - |
| Rimegepant | - | O | - | - |
| Risdiplam | O | O | O | O |
| Risperidone | X | O | X | X |
| Rituximab | O | O | O | O |
| Rivaroxaban | X | O | O | X |
| Ropivacaine | X | O | X | X |
| Rosuvastatin | O | O | X | X |
| Rucaparib | - | O | O | - |
| Ruxolitinib | X | X | X | O |
| Sacituzumab Govitecan-hziy | - | O | X | - |
| Satralizumab-mwge | - | O | O | O |
| Selpercatinib | - | O | O | O |
| Setmelanotide | - | O | O | - |
| Sevoflurane | X | O | X | X |
| Sildenafil | X | X | O | X |
| Simeprevir | - | O | X | X |
| Siponimod | - | O | O | O |
| Sodium Nitrite | - | O | X | - |
| Sodium Oxybate | - | O | O | - |
| Sodium Phenylbutyrate | O | O | O | X |
| Sofosbuvir | O | O | O | X |
| Sofosbuvir and Velpatasvir | - | O | O | X |
| Sofosbuvir, Velpatasvir, and Voxilaprevir | - | O | O | - |
| Sotorasib | - | O | - | - |
| Succimer | - | O | - | - |
| Succinylcholine | X | O | - | X |
| Sulfadiazine | O | O | - | O |
| Sulfamethoxazole and Trimethoprim | O | O | X | O |
| Sulfasalazine | O | O | - | O |
| Synthetic Conjugated Estrogens, A | - | O | - | - |
| Tafamidis | O | O | O | O |
| Tafenoquine | - | O | - | - |
| Talazoparib | O | O | O | - |
| Tamoxifen | O | O | X | X |
| Tamsulosin | X | O | X | X |
| Telaprevir | - | O | - | X |
| Tepotinib | O | O | - | O |
| Tetrabenazine | O | O | X | O |
| Thioguanine | X | O | X | - |
| Thioridazine | - | O | - | - |
| Ticagrelor | X | O | O | X |
| Tipiracil and Trifluridine | O | O | O | O |
| Tolazamide | - | O | - | - |
| Tolbutamide | - | O | - | - |
| Tolterodine | O | O | X | O |
| Toremifene | X | O | O | O |
| Tramadol | O | O | O | O |
| Trametinib | O | O | O | O |
| Trastuzumab | O | O | O | O |
| Tretinoin | X | O | X | O |
| Triheptanoin | - | O | X | - |
| Trimipramine | - | O | - | X |
| Tucatinib | - | O | X | - |
| Umeclidinium | O | O | X | X |
| Upadacitinib | O | O | X | X |
| Ustekinumab | O | O | O | X |
| Valbenazine | - | O | - | - |
| Valproic Acid | O | O | X | O |
| Vandetanib | O | X | O | O |
| Velaglucerase alfa | X | X | O | O |
| Vemurafenib | O | O | O | O |
| Venetoclax | O | O | O | X |
| Venlafaxine | X | O | X | O |
| Viloxazine | - | O | - | - |
| Viltolarsen | - | O | - | - |
| Vincristine | X | O | X | X |
| Vitamin C | O | X | - | X |
| Voriconazole | O | O | O | O |
| Vortioxetine | X | O | O | O |
| Voxelotor | - | O | X | - |
| Warfarin | O | O | - | O |

**S2 Table.** Contents of drug labeling in Korea

| **Drug** | **Therapeutic Area** | **ATC code** | **Biomarker** | | **Labelling Section** | **Date of permission** |
| --- | --- | --- | --- | --- | --- | --- |
| Abacavir | ANTIINFECTIVES FOR SYSTEMIC USE | J05AF06 | HLA-B | | Warnings and Precautions | 2010-06-03 |
| Abemaciclib | ANTINEOPLASTIC AND IMMUNOMODULATING AGENTS | L01EF03 | HR, HER2 | | Indications and Usage | 2019-05-01 |
| Ado-Trastuzumab Emtansine | ANTINEOPLASTIC AND IMMUNOMODULATING AGENTS | L01XC14 | HER2 | | Indications and Usage | 2014-01-24 |
| Aducanumab-avwa | NERVOUS SYSTEM | - | - | | - | - |
| Afatinib | ANTINEOPLASTIC AND IMMUNOMODULATING AGENTS | L01XE13 | EGFR | | Indications and Usage, Warnings and Precautions | 2014-01-29 |
| Alectinib | ANTINEOPLASTIC AND IMMUNOMODULATING AGENTS | L01XE36 | ALK | | Indications and Usage, Dosage and Administration, Warnings and Precautions | 2016-10-26 |
| Alglucosidase Alfa | ALIMENTARY TRACT AND METABOLISM\ | A16AB07 | GAA | | Indications and Usage | 2012-12-20 |
| Alirocumab | CARDIOVASCULAR SYSTEM | C10AX14 | X | |  | 2017-01-20 |
| Aliskiren | CARDIOVASCULAR SYSTEM | C09XA02 | - | | - | - |
| Allopurinol | MUSCULO-SKELETAL SYSTEM | M04AA01 | HLA-B*5801 | | Warnings and Precautions | 1970-09-25 |
| Alpelisib | ANTINEOPLASTIC AND IMMUNOMODULATING AGENTS | L01EM03 | - | | - | - |
| Amifampridine | NERVOUS SYSTEM | N07XX05 | - | | - | - |
| Amitriptyline | NERVOUS SYSTEM | N06AA09 | X | |  | 1970-02-20 |
| Amivantamab-vmjw | ANTINEOPLASTIC AND IMMUNOMODULATING AGENTS | - | - | | - | - |
| Amoxapine | NERVOUS SYSTEM | N06AA17 | X | |  | 1998-09-30 |
| Amphetamine | NERVOUS SYSTEM | N06BA01 | - | | - | - |
| Anakinra | ANTINEOPLASTIC AND IMMUNOMODULATING AGENTS | L04AC03 | - | | - | - |
| Anastrozole | ANTINEOPLASTIC AND IMMUNOMODULATING AGENTS | L02BG03 | ESR1, PGR | | Indications and Usage | 2000-12-16 |
| Arformoterol | RESPIRATORY SYSTEM | R03 | - | | - | - |
| Aripiprazole | NERVOUS SYSTEM | N05AX12 | X | |  | 2002-08-01 |
| Arsenic Trioxide | ANTINEOPLASTIC AND IMMUNOMODULATING AGENTS | L01XX27 | PML/RARA | | Indications and Usage | 2010-06-29 |
| Articaine and Epinephrine | NERVOUS SYSTEM | N01BB58 | X | |  | 2003-02-12 |
| Ascorbic Acid, PEG-3350, Potassium Chloride, Sodium Ascorbate, Sodium Chloride, and Sodium Sulfate | ALIMENTARY TRACT AND METABOLISM\ | A06AD65 | X | |  | 2011-08-05 |
| Atazanavir | ANTIINFECTIVES FOR SYSTEMIC USE | J05AE08 | X | |  | 2004-08-17 |
| Atezolizumab | ANTINEOPLASTIC AND IMMUNOMODULATING AGENTS | L01XC32 | EGFR, ALK | | Indications and Usage | 2017-01-12 |
| Atomoxetine | NERVOUS SYSTEM | N06BA09 | CYP2D6 | | Dosage and Administration, Warnings and Precautions | 2006-09-29 |
| Atorvastatin | CARDIOVASCULAR SYSTEM | C10AA05 | X | |  | 2004-10-25 |
| Avapritinib | ANTINEOPLASTIC AND IMMUNOMODULATING AGENTS | L01EX18 | - | | - | - |
| Avatrombopag | BLOOD AND BLOOD FORMING ORGANS | B02BX08 | - | | - | - |
| Avelumab | ANTINEOPLASTIC AND IMMUNOMODULATING AGENTS | L01XC31 | X | |  | 2019-03-22 |
| Axitinib | ANTINEOPLASTIC AND IMMUNOMODULATING AGENTS | L01XE17 | CYP3A4/5 | | Dosage and Administration, Warnings and Precautions | 2012-08-22 |
| Azathioprine | ANTINEOPLASTIC AND IMMUNOMODULATING AGENTS | L04AX01 | TPMT, NUDT15 | | Warnings and Precautions | 1992-09-25 |
| Belimumab | ANTINEOPLASTIC AND IMMUNOMODULATING AGENTS | L04AA26 | X | |  | 2013-06-21 |
| Belinostat | ANTINEOPLASTIC AND IMMUNOMODULATING AGENTS | L01XH04 | - | | - | - |
| Binimetinib | ANTINEOPLASTIC AND IMMUNOMODULATING AGENTS | L01EE03 | - | | - | - |
| Blinatumomab | ANTINEOPLASTIC AND IMMUNOMODULATING AGENTS | L01XC19 | X | |  | 2015-11-03 |
| Boceprevir | ANTIINFECTIVES FOR SYSTEMIC USE | J05AP03 | - | | - | - |
| Bosutinib | ANTINEOPLASTIC AND IMMUNOMODULATING AGENTS | - | - | | - | - |
| Brentuximab Vedotin | ANTINEOPLASTIC AND IMMUNOMODULATING AGENTS | L01XC12 | CD30 ALK | | Indications and Usage, Dosage and Administration, Warnings and Precautions | 2013-05-16 |
| Brexpiprazole | NERVOUS SYSTEM | N05AX16 | CYP2D6 | | Dosage and Administration, Warnings and Precautions | 2018-02-07 |
| Brigatinib | ANTINEOPLASTIC AND IMMUNOMODULATING AGENTS | L01EA04 | ALK | | Indications and Usage, Dosage and Administration | 2018-11-30 |
| Brivaracetam | NERVOUS SYSTEM | N03AX23 | X | |  | 2019-03-04 |
| Bupivacaine | NERVOUS SYSTEM | N01BB01 | - | | - | - |
| Bupropion | NERVOUS SYSTEM | N06AX12 | X | |  | 2002-01-18 |
| Busulfan | ANTINEOPLASTIC AND IMMUNOMODULATING AGENTS | L01AB01 | X | |  | 1996-11-14 |
| Cabotegravir and Rilpivirine | ANTIINFECTIVES FOR SYSTEMIC USE | J05AJ04 | - | | - | - |
| Cabozantinib | ANTINEOPLASTIC AND IMMUNOMODULATING AGENTS | L01XE26 | X | |  | 2017-09-26 |
| Capecitabine | ANTINEOPLASTIC AND IMMUNOMODULATING AGENTS | L01BC06 | DPYD | | Warnings and Precautions | 2000-05-13 |
| Capmatinib | ANTINEOPLASTIC AND IMMUNOMODULATING AGENTS | L01EX17 | MET exon 14 | | Indications and Usage, Dosage and Administration | 2021-11-23 |
| Carbamazepine | NERVOUS SYSTEM | N03AF01 | HLA-B*1502 | | Warnings and Precautions | 1984-01-30 |
| Carglumic Acid | ALIMENTARY TRACT AND METABOLISM\ | A16AA05 | NAGS (N-acetylglutamate synthase) | | Indications and Usage, Dosage and Administration, Warnings and Precautions | 2012-04-03 |
| Cariprazine | NERVOUS SYSTEM | N05AX15 | - | | - | - |
| Carisoprodol | MUSCULO-SKELETAL SYSTEM | M03BA02 | - | | - | - |
| Carvedilol | CARDIOVASCULAR SYSTEM | C07AG02 | CYP2D6 | | Warnings and Precautions | 1994-08-04 |
| Casimersen | NERVOUS SYSTEM | - | - | | - | - |
| Ceftriaxone | ANTIINFECTIVES FOR SYSTEMIC USE | J01DD04 | X | |  | 1987-03-25 |
| Celecoxib | ANTINEOPLASTIC AND IMMUNOMODULATING AGENTS | M01AH01 | X | |  | 2006-09-11 |
| Cemiplimab-rwlc | ANTINEOPLASTIC AND IMMUNOMODULATING AGENTS | L01XC33 | - | | - | - |
| Ceritinib | ANTINEOPLASTIC AND IMMUNOMODULATING AGENTS | L01XE28 | ALK | | Indications and Usage, Dosage and Administration, Warnings and Precautions | 2015-01-12 |
| Cerliponase Alfa | ALIMENTARY TRACT AND METABOLISM | A16AB17 | TPP1 | | Indications and Usage | 2020-09-08 |
| Cetuximab | ANTINEOPLASTIC AND IMMUNOMODULATING AGENTS | L01XC06 | EGFR, KRAS | | Indications and Usage, Dosage and Administration, Warnings and Precautions | 2009-03-13 |
| Cevimeline | NERVOUS SYSTEM | N07AX03 | - | | - | - |
| Chloroprocaine | NERVOUS SYSTEM | N01BA04 | - | | - | - |
| Chloroquine | ANTIPARASITIC PRODUCTS, INSECTICIDES AND REPELLENTS | P01BA02 | G6PD | | Warnings and Precautions | 1989-03-28 |
| Chlorpropamide | ALIMENTARY TRACT AND METABOLISM | A10BB02 | - | | - | - |
| Cholic acid | ALIMENTARY TRACT AND METABOLISM | A05AA03 | - | | - | - |
| Cisplatin | ANTINEOPLASTIC AND IMMUNOMODULATING AGENTS | L01XA01 | X | |  | 1984-08-03 |
| Citalopram | NERVOUS SYSTEM | N06AB10 | X | |  | 2004-12-07 |
| Clobazam | NERVOUS SYSTEM | N05BA09 | CYP2C19 | | Warnings and Precautions | 1981-02-12 |
| Clomipramine | NERVOUS SYSTEM | N06AA04 | X | |  | 1989-02-16 |
| Clopidogrel | BLOOD AND BLOOD FORMING ORGANS | B01AC04 | CYP2C19 | | Warnings and Precautions | 2005-05-24 |
| Clozapine | NERVOUS SYSTEM | N05AH02 | X | |  | 2003-05-07 |
| Cobimetinib | ANTINEOPLASTIC AND IMMUNOMODULATING AGENTS | L01XE38 | BRAF V600E, BRAF V600K | | Indications and Usage, Dosage and Administration | 2015-11-24 |
| Codeine | RESPIRATORY SYSTEM | R05DA04 | CYP2D6 | | Warnings and Precautions | 1994-09-22 |
| Crizanlizumab-tmca | BLOOD AND BLOOD FORMING ORGANS | B06AX01 | - | | - | - |
| Crizotinib | ANTINEOPLASTIC AND IMMUNOMODULATING AGENTS | L01XE16 | ALK, RIS-1 | | Indications and Usage, Dosage and Administration, Warnings and Precautions | 2011-12-29 |
| Dabrafenib | ANTINEOPLASTIC AND IMMUNOMODULATING AGENTS | L01XE23 | BRAF V600E, BRAF V600K | | Indications and Usage, Dosage and Administration, Warnings and Precautions | 2014-09-25 |
| Daclatasvir | ANTIINFECTIVES FOR SYSTEMIC USE | J05AP07 | X | |  | 2015-04-28 |
| Dacomitinib | ANTINEOPLASTIC AND IMMUNOMODULATING AGENTS | L01XE47 | EGFR | | Indications and Usage, Dosage and Administration, Warnings and Precautions | 2020-02-14 |
| Dapsone | ANTIINFECTIVES FOR SYSTEMIC USE | J04BA02 | G6PD | | Warnings and Precautions | 1983-08-09 |
| Darifenacin | GENITO URINARY SYSTEM AND SEX HORMONES | G04BD10 | - | | - | - |
| Darunavir | ANTIINFECTIVES FOR SYSTEMIC USE | J05AE10 | DRV-RAMs (Darunavir-Resistance associated mutations) | | Indications and Usage, Dosage and Administration | 2010-10-20 |
| Dasabuvir, Ombitasvir, Paritaprevir, and Ritonavir | ANTIINFECTIVES FOR SYSTEMIC USE | J05AP52 | - | | - | - |
| Dasatinib | ANTINEOPLASTIC AND IMMUNOMODULATING AGENTS | L01XE06 | BCR/ABL1 | | Indications and Usage, Dosage and Administration | 2007-01-25 |
| Denileukin Diftitox | ANTINEOPLASTIC AND IMMUNOMODULATING AGENTS | L01XX29 | - | | - | - |
| Desflurane | NERVOUS SYSTEM | N01AB07 | X | |  | 1997-08-16 |
| Desipramine | NERVOUS SYSTEM | N06AA01 | - | | - | - |
| Desvenlafaxine | NERVOUS SYSTEM | N06AX23 | X | |  | 2014-02-06 |
| Deutetrabenazine | NERVOUS SYSTEM | N07XX16 | CYP2D6 | | Warnings and Precautions | 2020-09-16 |
| Dexlansoprazole | ALIMENTARY TRACT AND METABOLISM\ | A02BC06 | X | |  | 2012-10-22 |
| Dextromethorphan and Quinidine | NERVOUS SYSTEM | N07XX59 | - | | - | - |
| Diazepam | NERVOUS SYSTEM | N05BA01 | X | |  | 1977-05-12 |
| Dinutuximab | ANTINEOPLASTIC AND IMMUNOMODULATING AGENTS | L01XC16 | - | | - | - |
| Divalproex sodium | NERVOUS SYSTEM | N03AG01 | POLG (polymerase gamma) | | Warnings and Precautions | 1995-10-07 |
| Docetaxel | ANTINEOPLASTIC AND IMMUNOMODULATING AGENTS | L01CD02 | HER2 | | Indications and Usage, Dosage and Administration | 2006-04-27 |
| Dolutegravir | ANTIINFECTIVES FOR SYSTEMIC USE | J05AX12 | INI genotypic resistance | | Dosage and Administration, Warnings and Precautions | 2014-08-29 |
| Donepezil | NERVOUS SYSTEM | N06DA02 | X | |  | 2000-08-04 |
| Dostarlimab-gxly | ANTINEOPLASTIC AND IMMUNOMODULATING AGENTS | L01XC40 | - | | - | - |
| Doxepin | NERVOUS SYSTEM | N06AA12 | X | |  | 2013-12-24 |
| Dronabinol | ALIMENTARY TRACT AND METABOLISM\ | A04AD10 | - | | - | - |
| Drospirenone and Ethinyl Estradiol | GENITO URINARY SYSTEM AND SEX HORMONES | G03AA12 | CYP1A2 | | Warnings and Precautions | 2008-02-04 |
| Duloxetine | NERVOUS SYSTEM | N06AX21 | X | |  | 2007-07-30 |
| Durvalumab | ANTINEOPLASTIC AND IMMUNOMODULATING AGENTS | L01XC28 | PD-L1(programmed cell death ligand-1) | | Warnings and Precautions | 2018-12-04 |
| Duvelisib | ANTINEOPLASTIC AND IMMUNOMODULATING AGENTS | L01EM04 | - | | - | - |
| Eculizumab | ANTINEOPLASTIC AND IMMUNOMODULATING AGENTS | L04AA25 | X | |  | 2010-01-22 |
| Efavirenz | ANTIINFECTIVES FOR SYSTEMIC USE | J05AG03 | CYP2B6 | | Warnings and Precautions | 2008-08-28 |
| Elagolix | SYSTEMIC HORMONAL PREPARATIONS, EXCL. SEX HORMONES AND INSULINS | H01CC03 | - | | - | - |
| Elbasvir and Grazoprevir | ANTIINFECTIVES FOR SYSTEMIC USE | J05AP54 | IFNL3 | | Dosage and Administration | 2016-11-21 |
| Elexacaftor, Ivacaftor, and Tezacaftor | RESPIRATORY SYSTEM | R07AX32 | - | | - | - |
| Eliglustat | ALIMENTARY TRACT AND METABOLISM\ | A16AX10 | CYP2D6 | | Indications and Usage, Dosage and Administration, Warnings and Precautions | 2015-11-12 |
| Elosulfase | ALIMENTARY TRACT AND METABOLISM\ | A16AB12 | GALNS | | Indications and Usage, Dosage and Administration, Warnings and Precautions | 2015-02-06 |
| Eltrombopag | BLOOD AND BLOOD FORMING ORGANS | B02BX05 | Factor V Leiden, AtⅢ | | Warnings and Precautions | 2010-03-12 |
| Emapalumab-lzsg | ANTINEOPLASTIC AND IMMUNOMODULATING AGENTS | L04AA39 | - | | - | - |
| Enasidenib | ANTINEOPLASTIC AND IMMUNOMODULATING AGENTS | L01XX59 | - | | - | - |
| Encorafenib | ANTINEOPLASTIC AND IMMUNOMODULATING AGENTS | L01EC03 | BRAF V600E | | Indications and Usage, Dosage and Administration, Warnings and Precautions | 2021-08-19 |
| Enflurane | ANTINEOPLASTIC AND IMMUNOMODULATING AGENTS | N01AB04 | - | | - | - |
| Entrectinib | ANTINEOPLASTIC AND IMMUNOMODULATING AGENTS | L01EX14 | NTRK, ROS1 | | Indications and Usage, Dosage and Administration, Warnings and Precautions | 2020-04-21 |
| Enzalutamide | ANTINEOPLASTIC AND IMMUNOMODULATING AGENTS | L02BB | CYP2C8 | | Dosage and Administration, Warnings and Precautions | 1900-01-01 |
| Erdafitinib | ANTINEOPLASTIC AND IMMUNOMODULATING AGENTS | L01EX16 | - | | - | - |
| Eribulin | ANTINEOPLASTIC AND IMMUNOMODULATING AGENTS | L01XX41 | X | |  | 2012-08-17 |
| Erlotinib | ANTINEOPLASTIC AND IMMUNOMODULATING AGENTS | L01XE03 | EGFR | | Indications and Usage, Dosage and Administration, Warnings and Precautions | 2005-07-29 |
| Erythromycin and Sulfisoxazole | ANTIINFECTIVES FOR SYSTEMIC USE | J01FA01 | - | | - | - |
| Escitalopram | NERVOUS SYSTEM | N06AB10 | CYP2C19, CYP2D6 | | Warnings and Precautions | 2004-12-07 |
| Esomeprazole | ALIMENTARY TRACT AND METABOLISM\ | A02BC05 | X | |  | 2000-10-19 |
| Estradiol and Progesterone | GENITO URINARY SYSTEM AND SEX HORMONES | G03FB06 | X | |  | 1993-02-01 |
| Eteplirsen | MUSCULO-SKELETAL SYSTEM | M09AX06 | - | | - | - |
| Ethinyl estradiol and Norelgestromin | GENITO URINARY SYSTEM AND SEX HORMONES | G03AA13 | - | | - | - |
| Everolimus | ANTINEOPLASTIC AND IMMUNOMODULATING AGENTS | L01XE10 | nonsteroidal-AIs, ESR, HER2 | | Indications and Usage | 2009-06-26 |
| Evinacumab-dgnb | ALIMENTARY TRACT AND METABOLISM | - | - | | - | - |
| Evolocumab | CARDIOVASCULAR SYSTEM | C10AX13 | heterozygous, homozygous | | Indications and Usage | 2017-04-13 |
| Exemestane | ANTINEOPLASTIC AND IMMUNOMODULATING AGENTS | L02BG06 | ESR | | Indications and Usage, Warnings and Precautions | 2008-07-24 |
| Fampridine | NERVOUS SYSTEM | N07XX07 | OCT2 | | Warnings and Precautions | 2014-06-23 |
| Fam-Trastuzumab Deruxtecan-nxki | ANTINEOPLASTIC AND IMMUNOMODULATING AGENTS | L01XC03 | - | | - | - |
| Fesoterodine | GENITO URINARY SYSTEM AND SEX HORMONES | G04BD11 | X | |  | 2008-10-02 |
| Flibanserin | GENITO URINARY SYSTEM AND SEX HORMONES | G02CX02 | - | | - | - |
| Fluorouracil | ANTINEOPLASTIC AND IMMUNOMODULATING AGENTS | L01BC02 | DPYD (DPD) | | Warnings and Precautions | 1976-04-14 |
| Fluoxetine | NERVOUS SYSTEM | N06AB03 | X | |  | 1997-10-21 |
| Flurbiprofen | MUSCULO-SKELETAL SYSTEM | M02AA19 | X | |  |  |
| Flutamide | ANTINEOPLASTIC AND IMMUNOMODULATING AGENTS | L02BB01 | - | | - | - |
| Fluvoxamine | NERVOUS SYSTEM | N06AB08 | X | |  | 1999-09-22 |
| Formoterol | RESPIRATORY SYSTEM | R03CC | X | |  | 1986-06-25 |
| Fosphenytoin | NERVOUS SYSTEM | N03AB05 | | HLA-B*1502 | Warnings and Precautions | 2008-06-20 |
| Fulvestrant | ANTINEOPLASTIC AND IMMUNOMODULATING AGENTS | L02BA03 | | ERBB2, HR | Indications and Usage |  |
| Galantamine | NERVOUS SYSTEM | N06DA04 | CYP2D6, CYP3A4 | | Dosage and Administration, Warnings and Precautions | 2007-09-27 |
| Gefitinib | ANTINEOPLASTIC AND IMMUNOMODULATING AGENTS | L01XE02 | EGFR | | Indications and Usage, Warnings and Precautions | 2003-06-14 |
| Gemtuzumab Ozogamicin | ANTINEOPLASTIC AND IMMUNOMODULATING AGENTS | L01XC05 | - | | - | - |
| Gilteritinib | ANTINEOPLASTIC AND IMMUNOMODULATING AGENTS | L01EX13 | FLT3 | | Indications and Usage, Dosage and Administration, Warnings and Precautions | 2020-03-06 |
| Givosiran | ALIMENTARY TRACT AND METABOLISM\ | A16AX16 | - | | - | - |
| Glimepiride | ALIMENTARY TRACT AND METABOLISM | A10BB12 | G6PD | | Warnings and Precautions | 1996-05-22 |
| Glipizide | ALIMENTARY TRACT AND METABOLISM | A10BB07 | X | |  | 1977-10-19 |
| Glyburide | ALIMENTARY TRACT AND METABOLISM | A10BB01 | G6PD | | Warnings and Precautions | 1970-06-01 |
| Golodirsen | MUSCULO-SKELETAL SYSTEM | M09AX08 | - | | - | - |
| Goserelin | ANTINEOPLASTIC AND IMMUNOMODULATING AGENTS | L02AE03 | ESR | | Indications and Usage | 1999-12-22 |
| Hydralazine | CARDIOVASCULAR SYSTEM | C02DB02 | X | |  | 1981-08-07 |
| Hydroxychloroquine | ANTIPARASITIC PRODUCTS, INSECTICIDES AND REPELLENTS | P01BA02 | G6PD | | Warnings and Precautions | 1989-03-28 |
| Ibritumomab | VARIOUS | V10XX02 | CD20 | | Indications and Usage | 2008-01-25 |
| Ibrutinib | ANTINEOPLASTIC AND IMMUNOMODULATING AGENTS | L01XE27 | X | |  | 2014-08-08 |
| Iloperidone | NERVOUS SYSTEM | N05AX14 | - | | - | - |
| Imatinib | ANTINEOPLASTIC AND IMMUNOMODULATING AGENTS | L01XE01 | BCR/ABL1, KIT, PDGFR | | Indications and Usage, Warnings and Precautions | 2006-11-30 |
| Imipramine | NERVOUS SYSTEM | N06AA02 | X | |  | 1982-12-03 |
| Indacaterol | RESPIRATORY SYSTEM | R03AC18 | UGT1A1 | | Warnings and Precautions | 2010-08-26 |
| Inebilizumab-cdon | ANTINEOPLASTIC AND IMMUNOMODULATING AGENTS | L04AA47 | AQP4 | | Indications and Usage | 2021-08-05 |
| Infigratinib | ANTINEOPLASTIC AND IMMUNOMODULATING AGENTS | - | - | | - | - |
| Inotersen | NERVOUS SYSTEM | N07XX15 | - | | - | - |
| Inotuzumab Ozogamicin | ANTINEOPLASTIC AND IMMUNOMODULATING AGENTS | L01XC26 | Philadelphia chromosome | | Indications and Usage, Warnings and Precautions | 2019-01-03 |
| Ipilimumab | ANTINEOPLASTIC AND IMMUNOMODULATING AGENTS | L01XC11 | X | |  | 2014-12-05 |
| Irinotecan | ANTINEOPLASTIC AND IMMUNOMODULATING AGENTS | L01XX19 | UGT1A1 | | Warnings and Precautions | 2001-12-14 |
| Isatuximab- irfc | ANTINEOPLASTIC AND IMMUNOMODULATING AGENTS | L01XC38 | - | | - | - |
| Isoflurane | NERVOUS SYSTEM | N01AB06 | X | |  | 2007-10-17 |
| Isoniazid | ANTIINFECTIVES FOR SYSTEMIC USE | J04AC01 | X | |  | 1997-07-31 |
| Isoniazid, Pyrazinamide, and Rifampin | ANTIINFECTIVES FOR SYSTEMIC USE | J04AM05 | X | |  | 2006-04-28 |
| Isosorbide Dinitrate | CARDIOVASCULAR SYSTEM | C01DA08 | X | |  | 1983-07-05 |
| Isosorbide Mononitrate | CARDIOVASCULAR SYSTEM | C01DA14 | X | |  | 1986-08-20 |
| Ivacaftor | RESPIRATORY SYSTEM | R07AX02 | - | | - | - |
| Ivacaftor and Lumacaftor | RESPIRATORY SYSTEM | R07AX30 | - | | - | - |
| Ivacaftor and Tezacaftor | RESPIRATORY SYSTEM | R07AX31 | - | | - | - |
| Ivosidenib | ANTINEOPLASTIC AND IMMUNOMODULATING AGENTS | L01XX62 | - | | - | - |
| Ixabepilone | ANTINEOPLASTIC AND IMMUNOMODULATING AGENTS | L01DC04 | - | | - | - |
| Lacosamide | NERVOUS SYSTEM | N03AX18 | X | |  | 2016-08-31 |
| Lansoprazole | ALIMENTARY TRACT AND METABOLISM\ | A02BC03 | X | |  | 1997-02-21 |
| Lapatinib | ANTINEOPLASTIC AND IMMUNOMODULATING AGENTS | L01XE07 | ERBB2, HR, HLA-DQA1, HLA-DRB1 | | Indications and Usage, Dosage and Administration | 2007-07-30 |
| Larotrectinib | ANTINEOPLASTIC AND IMMUNOMODULATING AGENTS | L01EX12 | NTRK | | Indications and Usage, Dosage and Administration, Warnings and Precautions | 2020-05-11 |
| Ledipasvir and Sofosbuvir | ANTIINFECTIVES FOR SYSTEMIC USE | J05AP51 | X | |  | 2015-10-13 |
| Lenalidomide | ANTINEOPLASTIC AND IMMUNOMODULATING AGENTS | L04AX04 | 5q | | Indications and Usage | 2009-12-28 |
| Lenvatinib | ANTINEOPLASTIC AND IMMUNOMODULATING AGENTS | L01XE29 | Microsatellite Instability, Mismatch Repair | | Indications and Usage | 2015-10-07 |
| Lesinurad | MUSCULO-SKELETAL SYSTEM | M04AB05 | - | | - | - |
| Letrozole | ANTINEOPLASTIC AND IMMUNOMODULATING AGENTS | L02BG04 | ESR, PGR | | Indications and Usage | 2001-12-11 |
| Lidocaine and Prilocaine | NERVOUS SYSTEM | N01BB | G6PD | | Warnings and Precautions | 1994-03-12 |
| Lidocaine and Tetracaine | NERVOUS SYSTEM | N01BB52 | - | | - | - |
| Lofexidine | NERVOUS SYSTEM | N07BC04 | - | | - | - |
| Lomitapide | CARDIOVASCULAR SYSTEM | C10AX12 | - | | - | - |
| Lonafarnib | ALIMENTARY TRACT AND METABOLISM | - | - | | - | - |
| Lorlatinib | ANTINEOPLASTIC AND IMMUNOMODULATING AGENTS | L01ED05 | - | | - | - |
| Lumasiran | ALIMENTARY TRACT AND METABOLISM | A16AX18 | - | | - | - |
| Luspatercept–aamt | BLOOD AND BLOOD FORMING ORGANS | B03XA06 | - | | - | - |
| Lusutrombopag | BLOOD AND BLOOD FORMING ORGANS | B02BX07 | - | | - | - |
| Lutetium Dotatate Lu-177 | VARIOUS | V10XX04 | SSTR | | Warnings and Precautions | 2020-07-09 |
| Mafenide | DERMATOLOGICALS | D06BA03 | G6PD | | Warnings and Precautions | 2003-04-14 |
| Maraviroc | ANTIINFECTIVES FOR SYSTEMIC USE | J05AX09 | CCR5 | | Indications and Usage, Warnings and Precautions | 2008-03-07 |
| Margetuximab-cmkb | ANTINEOPLASTIC AND IMMUNOMODULATING AGENTS | - | - | | - | - |
| Meclizine | RESPIRATORY SYSTEM | R06AE05 | X | |  | 1996-12-10 |
| Meloxicam | MUSCULO-SKELETAL SYSTEM | M01AC06 | X | |  | 2003-07-11 |
| Mepivacaine | NERVOUS SYSTEM | N01BB03 | X | |  | 1986-09-08 |
| Mercaptopurine | ANTINEOPLASTIC AND IMMUNOMODULATING AGENTS | L01BB02 | TPMT | | Warnings and Precautions | 1988-06-10 |
| Methylene Blue | VARIOUS | V03AB | G6PD | | Warnings and Precautions | 2017-12-14 |
| Metoclopramide | ALIMENTARY TRACT AND METABOLISM\ | A03FA01 | CYPB5R | | Warnings and Precautions | 1972-02-10 |
| Metoprolol | CARDIOVASCULAR SYSTEM | C07AB02 | X | |  | 1981-03-04 |
| Metreleptin | ALIMENTARY TRACT AND METABOLISM | A16AA07 | - | | - | - |
| Midostaurin | ANTINEOPLASTIC AND IMMUNOMODULATING AGENTS | L01XE39 | FLT3 | | Indications and Usage, Dosage and Administration, Warnings and Precautions | 2019-02-14 |
| Migalastat | ALIMENTARY TRACT AND METABOLISM\ | A16AX14 | GLA | | Indications and Usage, Warnings and Precautions | 2017-12-20 |
| Mirabegron | GENITO URINARY SYSTEM AND SEX HORMONES | G04BD12 | X | |  | 2013-12-31 |
| Mivacurium | MUSCULO-SKELETAL SYSTEM | M03AC10 | - | | - | - |
| Modafinil | NERVOUS SYSTEM | N06BA07 | CYP3A4  CYP2C19 | | Dosage and Administration | 2002-05-02 |
| Moviprep | Various | - | - | | - | - |
| Mycophenolic Acid | ANTINEOPLASTIC AND IMMUNOMODULATING AGENTS | L04AA06 | HGPRT | | Warnings and Precautions | 2007-06-18 |
| Nalidixic Acid | ANTIINFECTIVES FOR SYSTEMIC USE | J01MB02 | X | |  | 2004-02-03 |
| Nebivolol | CARDIOVASCULAR SYSTEM | C07AB12 | X | |  | 2006-08-28 |
| Nefazodone | NERVOUS SYSTEM | N06AX06 | - | | - | - |
| Neratinib | ANTINEOPLASTIC AND IMMUNOMODULATING AGENTS | L01EH02 | HR, HER2 | | Indications and Usage | 2021-10-19 |
| Nilotinib | ANTINEOPLASTIC AND IMMUNOMODULATING AGENTS | L01XE08 | ABL1, BCR | | Indications and Usage | 2007-10-26 |
| Niraparib | ANTINEOPLASTIC AND IMMUNOMODULATING AGENTS | L01XX54 | BRCA , HRD | | Indications and Usage, Dosage and Administration, Warnings and Precautions | 2019-03-22 |
| Nitrofurantoin | ANTIINFECTIVES FOR SYSTEMIC USE | J01XE01 | X | |  | 2018-03-23 |
| Nivolumab | ANTINEOPLASTIC AND IMMUNOMODULATING AGENTS | L01XC17 | EGFR ALK  PD-L1(CD274) | | Indications and Usage | 2015-03-20 |
| Norfloxacin | ANTIINFECTIVES FOR SYSTEMIC USE | J01MA06 | G6PD | | Warnings and Precautions | 1984-09-04 |
| Nortriptyline | NERVOUS SYSTEM | N06AA10 | X | |  | 1973-01-17 |
| Nusinersen | MUSCULO-SKELETAL SYSTEM | M09AX07 | SMN2 | | Warnings and Precautions | 2017-12-29 |
| Obinutuzumab | ANTINEOPLASTIC AND IMMUNOMODULATING AGENTS | L01XC15 | X | |  | 2014-09-22 |
| Ofatumumab | Antineoplastic and immunomodulating agents | L01XC10 | - | | - | - |
| Olaparib | ANTINEOPLASTIC AND IMMUNOMODULATING AGENTS | L01XX46 | BRCA | | Indications and Usage, Warnings and Precautions | 2015-08-05 |
| Olaratumab | ANTINEOPLASTIC AND IMMUNOMODULATING AGENTS | L01XC27 | - | | - | - |
| Oliceridine | NERVOUS SYSTEM | - | - | | - | - |
| Omacetaxine | ANTINEOPLASTIC AND IMMUNOMODULATING AGENTS | L01XX40 | - | | - | - |
| Ombitasvir, Paritaprevir, and Ritonavir | ANTIINFECTIVES FOR SYSTEMIC USE | J05AP53 | - | | - | - |
| Omeprazole | ALIMENTARY TRACT AND METABOLISM\ | A02BC01 | X | |  | 1992-09-01 |
| Ondansetron | ALIMENTARY TRACT AND METABOLISM\ | A04AA01 | X | |  | 1999-01-04 |
| Osimertinib | ANTINEOPLASTIC AND IMMUNOMODULATING AGENTS | L01XE35 | EGFR | | Indications and Usage, Dosage and Administration, Warnings and Precautions | 2016-05-19 |
| Ospemifene | GENITO URINARY SYSTEM AND SEX HORMONES | G03XC05 | - | | - | - |
| Oxcarbazepine | NERVOUS SYSTEM | N03AF02 | HLA-B | | Warnings and Precautions | 2000-08-03 |
| Oxymetazoline and Tetracaine | RESPIRATORY SYSTEM | R01AA05 | - | | - | - |
| Palbociclib | ANTINEOPLASTIC AND IMMUNOMODULATING AGENTS | L01XE33 | ERBB2, HR | | Indications and Usage, Warnings and Precautions | 2016-08-29 |
| Paliperidone | NERVOUS SYSTEM | N05AX13 | X | |  | 2010-07-26 |
| Palonosetron | ALIMENTARY TRACT AND METABOLISM\ | A04AA05 | X | |  | 2007-10-24 |
| Panitumumab | ANTINEOPLASTIC AND IMMUNOMODULATING AGENTS | L01XC08 | - | | - | - |
| Pantoprazole | ALIMENTARY TRACT AND METABOLISM\ | A02BC02 | X | |  | 1999-04-14 |
| Parathyroid Hormone | SYSTEMIC HORMONAL PREPARATIONS, EXCL. SEX HORMONES AND INSULINS | H05AA03 | - | | - | - |
| Paroxetine | NERVOUS SYSTEM | N06AB05 | X | |  | 1993-11-20 |
| Patisiran | NERVOUS SYSTEM | N07XX12 | - | | - | - |
| Pazopanib | ANTINEOPLASTIC AND IMMUNOMODULATING AGENTS | L01XE11 | UGT1A1 | | Warnings and Precautions | 2010-08-11 |
| Peginterferon Alfa-2b | ANTINEOPLASTIC AND IMMUNOMODULATING AGENTS | L03AB10 | X | |  | 2013-11-27 |
| Pegloticase | MUSCULO-SKELETAL SYSTEM | M04AX02 | - | | - | - |
| Pembrolizumab | ANTINEOPLASTIC AND IMMUNOMODULATING AGENTS | L01XC18 | PD-L1, EGFR, ALK | | Indications and Usage, Dosage and Administration, Warnings and Precautions | 2015-03-20 |
| Pemigatinib | ANTINEOPLASTIC AND IMMUNOMODULATING AGENTS | L01EX20 | - | | - | - |
| Perphenazine | NERVOUS SYSTEM | N05AB03 | X | |  | 1985-08-12 |
| Pertuzumab | ANTINEOPLASTIC AND IMMUNOMODULATING AGENTS | L01XC13 | ERBB2 | | Indications and Usage | 2013-05-29 |
| Phenytoin | NERVOUS SYSTEM | N03AB02 | X | |  | 1970-02-06 |
| Pimozide | NERVOUS SYSTEM | N05AG02 | CYP2D6 | | Warnings and Precautions | 1985-05-14 |
| Piroxicam | MUSCULO-SKELETAL SYSTEM | M01AC01 | X | |  | 1984-09-21 |
| Pitolisant | NERVOUS SYSTEM | N07XX11 | CYP2D6 | | Dosage and Administration, Warnings and Precautions | 2020-12-30 |
| Ponatinib | ANTINEOPLASTIC AND IMMUNOMODULATING AGENTS | L01XE24 | BCR-ABL1 T315I | | Indications and Usage | 2017-06-26 |
| Pralsetinib | ANTINEOPLASTIC AND IMMUNOMODULATING AGENTS | - | - | | - | - |
| Prasugrel | BLOOD AND BLOOD FORMING ORGANS | B01AC22 | X | |  | 2010-07-29 |
| Primaquine | ANTIPARASITIC PRODUCTS, INSECTICIDES AND REPELLENTS | P01BA03 | G6PD | | Warnings and Precautions | 1999-05-11 |
| Probenecid | MUSCULO-SKELETAL SYSTEM | M04AB01 | - | | - | - |
| Procainamide | CARDIOVASCULAR SYSTEM | C01BA02 | - | | - | - |
| Propafenone | CARDIOVASCULAR SYSTEM | C01BC03 | X | |  | 1997-05-27 |
| Propranolol | CARDIOVASCULAR SYSTEM | C07AA05 | X | |  | 1974-11-05 |
| Protriptyline | NERVOUS SYSTEM | N06AA11 | - | | - | - |
| Quinidine | CARDIOVASCULAR SYSTEM | C01BA01 | - | | - | - |
| Quinine Sulfate | ANTIPARASITIC PRODUCTS, INSECTICIDES AND REPELLENTS | P01BC01 | - | | - | - |
| Rabeprazole | ALIMENTARY TRACT AND METABOLISM\ | A02BC04 | X | |  | 2000-02-21 |
| Raloxifene | GENITO URINARY SYSTEM AND SEX HORMONES | G03XC01 | X | |  | 2001-07-18 |
| Raltegravir | ANTIINFECTIVES FOR SYSTEMIC USE | J05AX08 | X | |  | 2008-06-23 |
| Ramucirumab | ANTINEOPLASTIC AND IMMUNOMODULATING AGENTS | L01XC21 | EGFR, ALK | | Indications and Usage, Dosage and Administration, Warnings and Precautions | 2015-04-15 |
| Ranolazine | CARDIOVASCULAR SYSTEM | C01EB18 | CYP2D6 | | Warnings and Precautions | 2020-03-16 |
| Rasburicase | VARIOUS | V03AF07 | G6PD | | Warnings and Precautions | 2006-04-20 |
| Regorafenib | ANTINEOPLASTIC AND IMMUNOMODULATING AGENTS | L01XE21 | RAS | | Indications and Usage | 2013-08-22 |
| Ribociclib | ANTINEOPLASTIC AND IMMUNOMODULATING AGENTS | L01XE42 | HR, HER2 | | Indications and Usage, Warnings and Precautions | 2019-10-30 |
| Rimegepant | NERVOUS SYSTEM | - | - | | - | - |
| Risdiplam | MUSCULO-SKELETAL SYSTEM | M09AX10 | SMN1, SMN2 | | Warnings and Precautions | 2020-11-02 |
| Risperidone | NERVOUS SYSTEM | N05AX08 | X | |  | 1996-05-06 |
| Rituximab | ANTINEOPLASTIC AND IMMUNOMODULATING AGENTS | L01XC02 | MS4A1 (CD20 antigen) | | Indications and Usage | 2003-11-21 |
| Rivaroxaban | BLOOD AND BLOOD FORMING ORGANS | B01AF01 | X | |  | 2009-04-13 |
| Ropivacaine | NERVOUS SYSTEM | N01BB09 | X | |  | 1998-12-26 |
| Rosuvastatin | CARDIOVASCULAR SYSTEM | C10AA07 | SLCO1B1 | | Warnings and Precautions | 2002-01-15 |
| Rucaparib | ANTINEOPLASTIC AND IMMUNOMODULATING AGENTS | L01XK03 | - | | - | - |
| Ruxolitinib | ANTINEOPLASTIC AND IMMUNOMODULATING AGENTS | L01XE18 | X | |  | 2013-01-21 |
| Sacituzumab Govitecan-hziy | ANTINEOPLASTIC AND IMMUNOMODULATING AGENTS | L01 | - | | - | - |
| Satralizumab-mwge | ANTINEOPLASTIC AND IMMUNOMODULATING AGENTS | L04AC19 | - | | - | - |
| Selpercatinib | ANTINEOPLASTIC AND IMMUNOMODULATING AGENTS | L01 | - | | - | - |
| Setmelanotide | ALIMENTARY TRACT AND METABOLISM | - | - | | - | - |
| Sevoflurane | NERVOUS SYSTEM | N01AB08 | X | |  | 2000-02-14 |
| Sildenafil | GENITO URINARY SYSTEM AND SEX HORMONES | G04BE03 | X | |  | 2004-05-03 |
| Simeprevir | ANTIINFECTIVES FOR SYSTEMIC USE | J05AP05 | - | | - | - |
| Siponimod | ANTINEOPLASTIC AND IMMUNOMODULATING AGENTS | L04AA42 | - | | - | - |
| Sodium Nitrite | VARIOUS | V03AB08 | - | | - | - |
| Sodium Oxybate | NERVOUS SYSTEM | N01AX11 | - | | - | - |
| Sodium Phenylbutyrate | ALIMENTARY TRACT AND METABOLISM\ | A16AX03 | CPS, OTC, AS | | Indications and Usage | 2005-06-20 |
| Sofosbuvir | ANTIINFECTIVES FOR SYSTEMIC USE | J05AP08 | IL28B | | Dosage and Administration | 2015-09-10 |
| Sofosbuvir and Velpatasvir | ANTIINFECTIVES FOR SYSTEMIC USE | J05AP55 | - | | - | - |
| Sofosbuvir, Velpatasvir, and Voxilaprevir | ANTIINFECTIVES FOR SYSTEMIC USE | J05AP56 | - | | - | - |
| Sotorasib | ANTINEOPLASTIC AND IMMUNOMODULATING AGENTS | - | - | | - | - |
| Succimer | VARIOUS | V09CA02 | - | | - | - |
| Succinylcholine | MUSCULO-SKELETAL SYSTEM | M03AB01 | X | |  | 1993-12-18 |
| Sulfadiazine | DERMATOLOGICALS | D06BA01 | G6PD | | Warnings and Precautions |  |
| Sulfamethoxazole and Trimethoprim | ANTIINFECTIVES FOR SYSTEMIC USE | J01EE01 | G6PD | | Warnings and Precautions | 1970-09-25 |
| Sulfasalazine | ALIMENTARY TRACT AND METABOLISM\ | A07EC01 | G6PD | | Warnings and Precautions | 1998-03-31 |
| Synthetic Conjugated Estrogens, A | GENITO URINARY SYSTEM AND SEX HORMONES | - | - | | - | - |
| Tafamidis | NERVOUS SYSTEM | N07XX08 | TTR | | Indications and Usage, Warnings and Precautions | 2020-08-19 |
| Tafenoquine | ANTIINFECTIVES FOR SYSTEMIC USE | P01BA07 | - | | - | - |
| Talazoparib | ANTINEOPLASTIC AND IMMUNOMODULATING AGENTS | L01XK04 | BRCA, ERBB2 (HER2) | | Indications and Usage, Dosage and Administration, Warnings and Precautions | 2020-07-30 |
| Tamoxifen | ANTINEOPLASTIC AND IMMUNOMODULATING AGENTS | L02BA01 | CYP2D6 | | Warnings and Precautions | 1988-06-10 |
| Tamsulosin | GENITO URINARY SYSTEM AND SEX HORMONES | G04CA02 | X | |  | 2003-05-21 |
| Telaprevir | ANTIINFECTIVES FOR SYSTEMIC USE | J05AP02 | - | | - | - |
| Tepotinib | ANTINEOPLASTIC AND IMMUNOMODULATING AGENTS | L01EX21 | MET/ALK, EGFR | | Indications and Usage, Dosage and Administration, Warnings and Precautions | 2021-11-23 |
| Tetrabenazine | NERVOUS SYSTEM | N07XX06 | CYP2D6 | | Warnings and Precautions | 2020-09-16 |
| Thioguanine | ANTINEOPLASTIC AND IMMUNOMODULATING AGENTS | L01BB03 | X | |  | 2005-03-21 |
| Thioridazine | NERVOUS SYSTEM | N05AC02 | - | | - | - |
| Ticagrelor | BLOOD AND BLOOD FORMING ORGANS | B01AC24 | X | |  | 2011-07-22 |
| Tipiracil and Trifluridine | ANTINEOPLASTIC AND IMMUNOMODULATING AGENTS | L01BC59 | ERBB2 (HER2), RAS | | Indications and Usage | 2019-10-17 |
| Tolazamide | ALIMENTARY TRACT AND METABOLISM | A10BB05 | - | | - | - |
| Tolbutamide | ALIMENTARY TRACT AND METABOLISM | A10BB03 | - | | - | - |
| Tolterodine | GENITO URINARY SYSTEM AND SEX HORMONES | G04BD07 | CYP2D6 | | Warnings and Precautions | 2005-07-26 |
| Toremifene | ANTINEOPLASTIC AND IMMUNOMODULATING AGENTS | L02BA02 | X | |  | 1999-09-04 |
| Tramadol | NERVOUS SYSTEM | N02AX02 | CYP2D6 | | Warnings and Precautions | 1981-07-28 |
| Trametinib | ANTINEOPLASTIC AND IMMUNOMODULATING AGENTS | L01XE25 | BRAF V600E, BRAF V600K, G6PD | | Indications and Usage, Dosage and Administration, Warnings and Precautions | 2015-10-01 |
| Trastuzumab | ANTINEOPLASTIC AND IMMUNOMODULATING AGENTS | L01XC03 | ERBB2 | | Indications and Usage, Dosage and Administration, Warnings and Precautions | 2005-07-19 |
| Tretinoin | ANTINEOPLASTIC AND IMMUNOMODULATING AGENTS | L01XX14 | X | |  | 1995-05-03 |
| Triheptanoin | ALIMENTARY TRACT AND METABOLISM\ | A16AX17 | - | | - | - |
| Trimipramine | NERVOUS SYSTEM | N06AA06 | - | | - | - |
| Tucatinib | ANTINEOPLASTIC AND IMMUNOMODULATING AGENTS | L01EH03 | - | | - | - |
| Umeclidinium | RESPIRATORY SYSTEM | R03AL03 | CYP2D6 | | Warnings and Precautions | 2014-07-10 |
| Upadacitinib | ANTINEOPLASTIC AND IMMUNOMODULATING AGENTS | L04AA44 | CYP3A4 | | Warnings and Precautions | 2020-06-04 |
| Ustekinumab | ANTINEOPLASTIC AND IMMUNOMODULATING AGENTS | L04AC05 | IL-12, IL-23 | | Warnings and Precautions | 2011-06-21 |
| Valbenazine | NERVOUS SYSTEM | N07XX13 | - | | - | - |
| Valproic Acid | NERVOUS SYSTEM | N03AG01 | CPT2 | | Warnings and Precautions | 1980-10-08 |
| Vandetanib | ANTINEOPLASTIC AND IMMUNOMODULATING AGENTS | L01XE12 | CYP3A4 | | Warnings and Precautions | 2013-05-24 |
| Velaglucerase alfa | ALIMENTARY TRACT AND METABOLISM\ | A16AB10 | X | |  | 2014-08-29 |
| Vemurafenib | ANTINEOPLASTIC AND IMMUNOMODULATING AGENTS | L01XE15 | BRAF V600E | | Indications and Usage, Dosage and Administration, Warnings and Precautions | 2012-07-23 |
| Venetoclax | ANTINEOPLASTIC AND IMMUNOMODULATING AGENTS | L01XX52 | 17p, 11q, TP53 | | Warnings and Precautions (Clinical data) | 2019-05-29 |
| Venlafaxine | NERVOUS SYSTEM | N06AX16 | X | |  | 2003-11-21 |
| Viloxazine | NERVOUS SYSTEM | N06AX09 | - | | - | - |
| Viltolarsen | NERVOUS SYSTEM | - | - | | - | - |
| Vincristine | ANTINEOPLASTIC AND IMMUNOMODULATING AGENTS | L01CA02 | X | |  | 1986-09-08 |
| Vitamin C | Alimentary tract and metabolism | A11GA01 | G6PD | | Warnings and Precautions | 1999-04-26 |
| Voriconazole | NERVOUS SYSTEM | J02AC03 | CYP3A4 | | Warnings and Precautions | 2001-07-30 |
| Vortioxetine | NERVOUS SYSTEM | N06AX26 | X | |  | 2014-08-19 |
| Voxelotor | BLOOD AND BLOOD FORMING ORGANS | - | - | | - | - |
| Warfarin | BLOOD AND BLOOD FORMING ORGANS | B01AA03 | PT/INR, CYP2C9, VKORC1 | | Dosage and Administration, Warnings and Precautions | 1985-05-28 |

**S3 Table.** Contents of drug labeling in the United States

| **Drug** | **Therapeutic Area** | **ATC code** | **Biomarker** | **Labelling Section** |
| --- | --- | --- | --- | --- |
| Abacavir | ANTIINFECTIVES FOR SYSTEMIC USE | J05AF06 | HLA-B | Boxed Warning, Dosage and Administration, Contraindications, Warnings and Precautions |
| Abemaciclib | ANTINEOPLASTIC AND IMMUNOMODULATING AGENTS | L01EF03 | ESR (Hormone Receptor), ERBB2 (HER2) | Indications and Usage, Adverse Reactions, Clinical Studies |
| Ado-Trastuzumab Emtansine | ANTINEOPLASTIC AND IMMUNOMODULATING AGENTS | L01XC14 | ERBB2 (HER2) | Indications and Usage, Dosage and Administration, Adverse Reactions, Clinical Pharmacology, Clinical Studies |
| Aducanumab-avwa | NERVOUS SYSTEM | - | APOE | Warnings and Precautions, Clinical Studies |
| Afatinib | ANTINEOPLASTIC AND IMMUNOMODULATING AGENTS | L01XE13 | EGFR | Indications and Usage, Dosage and Administration, Adverse Reactions, Clinical Studies |
| Alectinib | ANTINEOPLASTIC AND IMMUNOMODULATING AGENTS | L01XE36 | ALK, ERBB2 (HER2), ESR (Hormone Receptor) | Indications and Usage, Dosage and Administration, Adverse Reactions, Clinical Pharmacology, Clinical Studies |
| Alglucosidase Alfa | ALIMENTARY TRACT AND METABOLISM\ | A16AB07 | GAA | Warnings and Precautions |
| Alirocumab | CARDIOVASCULAR SYSTEM | C10AX14 | X |  |
| Aliskiren | CARDIOVASCULAR SYSTEM | C09XA02 | X |  |
| Allopurinol | MUSCULO-SKELETAL SYSTEM | M04AA01 | HLA-B | Warnings |
| Alpelisib | ANTINEOPLASTIC AND IMMUNOMODULATING AGENTS | L01EM03 | HER2, ESR, PIK3CA | Indication and Usage, Dosage and Administration, Adverse Reactions, Clinical Studies |
| Amifampridine | NERVOUS SYSTEM | N07XX05 | NAT2 | Dosage and Administration, Adverse Reactions, Use in Specific Populations, Clinical Pharmacology |
| Amitriptyline | NERVOUS SYSTEM | N06AA09 | CYP2D6 | Precautions |
| Amivantamab-vmjw | ANTINEOPLASTIC AND IMMUNOMODULATING AGENTS | - | EGFR | Indications and Usage, Dosage and Administration, Adverse Reactions, Clinical Studies |
| Amoxapine | NERVOUS SYSTEM | N06AA17 | CYP2D6 | Precautions |
| Amphetamine | NERVOUS SYSTEM | N06BA01 | CYP2D6 | Clinical Pharmacology |
| Anakinra | ANTINEOPLASTIC AND IMMUNOMODULATING AGENTS | L04AC03 | NLRP3 | Indications and Usage, Dosage and Administration, Warnings and Precautions, Adverse Reactions, Use in Specific Populations, Clinical Pharmacology, Clinical Studies |
| Anastrozole | ANTINEOPLASTIC AND IMMUNOMODULATING AGENTS | L02BG03 | ESR, PGR (Hormone Receptor) | Indications and Usage, Adverse Reactions, Drug Interactions, Clinical Studies |
| Arformoterol | RESPIRATORY SYSTEM | R03 | UGT1A1, CYP2D6 | Clinical Pharmacology |
| Aripiprazole | NERVOUS SYSTEM | N05AX12 | CYP2D6 | Dosage and Administration, Use in Specific Populations, Clinical Pharmacology |
| Arsenic Trioxide | ANTINEOPLASTIC AND IMMUNOMODULATING AGENTS | L01XX27 | PML-RARA | Indications and Usage, Clinical Studies |
| Articaine and Epinephrine | NERVOUS SYSTEM | N01BB58 | G6PD, Nonspecific (Congenital Methemoglobinemia) | Warnings and Precautions |
| Ascorbic Acid, PEG-3350, Potassium Chloride, Sodium Ascorbate, Sodium Chloride, and Sodium Sulfate | ALIMENTARY TRACT AND METABOLISM\ | A06AD65 | G6PD | Warnings and Precautions |
| Atazanavir | ANTIINFECTIVES FOR SYSTEMIC USE | J05AE08 | X |  |
| Atezolizumab | ANTINEOPLASTIC AND IMMUNOMODULATING AGENTS | L01XC32 | CD274 (PD-L1), Gene Signature (T-effector), EGFR, ALK | Indications and Usage, Dosage and Administration, Adverse Reactions, Clinical Pharmacology, Clinical Studies |
| Atomoxetine | NERVOUS SYSTEM | N06BA09 | CYP2D6 | Dosage and Administration, Warnings and Precautions, Adverse Reactions, Drug Interactions, Use in Specific Populations, Clinical Pharmacology |
| Atorvastatin | CARDIOVASCULAR SYSTEM | C10AA05 | X |  |
| Avapritinib | ANTINEOPLASTIC AND IMMUNOMODULATING AGENTS | L01EX18 | PDGFRA | Indications and Usage, Dosage and Administration, Clinical Studies |
| Avatrombopag | BLOOD AND BLOOD FORMING ORGANS | B02BX08 | F2 (Prothrombin), F5 (Factor V Leiden), PROC, PROS1, SERPINC1 (Antithrombin III) / CYP2C9 | Warnings and Precautions / Clinical Pharmacology |
| Avelumab | ANTINEOPLASTIC AND IMMUNOMODULATING AGENTS | L01XC31 | CD274 (PD-L1) | Clinical Studies |
| Axitinib | ANTINEOPLASTIC AND IMMUNOMODULATING AGENTS | L01XE17 | X |  |
| Azathioprine | ANTINEOPLASTIC AND IMMUNOMODULATING AGENTS | L04AX01 | TPMT, NUDT15 | Dosage and Administration,  Warnings, Precautions, Drug Interactions, Adverse Reactions, Clinical Pharmacology |
| Belimumab | ANTINEOPLASTIC AND IMMUNOMODULATING AGENTS | L04AA26 | X |  |
| Belinostat | ANTINEOPLASTIC AND IMMUNOMODULATING AGENTS | L01XH04 | UGT1A1 | Dosage and Administration, Clinical Pharmacology |
| Binimetinib | ANTINEOPLASTIC AND IMMUNOMODULATING AGENTS | L01EE03 | BRAF, UGT1A1 | Indications and Usage, Dosage and Administration, Warnings and Precautions, Adverse Reactions, Use in Specific Populations, Clinical Studies,  Clinical Pharmacology |
| Blinatumomab | ANTINEOPLASTIC AND IMMUNOMODULATING AGENTS | L01XC19 | BCR-ABL1 (Philadelphia chromosome) | Adverse Reactions, Clinical Studies |
| Boceprevir | ANTIINFECTIVES FOR SYSTEMIC USE | J05AP03 | IFNL3 (IL28B) | Clinical Pharmacology |
| Bosutinib | ANTINEOPLASTIC AND IMMUNOMODULATING AGENTS | - | BCR-ABL1 (Philadelphia chromosome) | Indications and Usage, Dosage and Administration, Warnings and Precautions, Adverse Reactions, Use in Specific Populations, Clinical Studies |
| Brentuximab Vedotin | ANTINEOPLASTIC AND IMMUNOMODULATING AGENTS | L01XC12 | ALK, TNFRSF8 (CD30) | Clinical Studies / Indications and Usage, Dosage and Administration, Adverse Reactions, Use in Specific Populations, Clinical Studies |
| Brexpiprazole | NERVOUS SYSTEM | N05AX16 | CYP2D6 | Dosage and Administration, Use in Specific Populations, Clinical Pharmacology |
| Brigatinib | ANTINEOPLASTIC AND IMMUNOMODULATING AGENTS | L01EA04 | ALK | Indications and Usage, Dosage and Administration, Adverse Reactions, Clinical Studies |
| Brivaracetam | NERVOUS SYSTEM | N03AX23 | CYP2C19 | Clinical Pharmacology |
| Bupivacaine | NERVOUS SYSTEM | N01BB01 | G6PD | Warnings |
| Bupropion | NERVOUS SYSTEM | N06AX12 | CYP2D6 | Clinical Pharmacology |
| Busulfan | ANTINEOPLASTIC AND IMMUNOMODULATING AGENTS | L01AB01 | BCR-ABL1 (Philadelphia chromosome) | Clinical Studies |
| Cabotegravir and Rilpivirine | ANTIINFECTIVES FOR SYSTEMIC USE | J05AJ04 | HLA-B/UGT1A1 | Clinical Studies/Clinical Pharmacology |
| Cabozantinib | ANTINEOPLASTIC AND IMMUNOMODULATING AGENTS | L01XE26 | RET | Clinical Studies |
| Capecitabine | ANTINEOPLASTIC AND IMMUNOMODULATING AGENTS | L01BC06 | DPYD | Warnings and Precautions, Patient Counseling Information |
| Capmatinib | ANTINEOPLASTIC AND IMMUNOMODULATING AGENTS | L01EX17 | MET | Indications and Usage, Dosage and Administration, Clinical Studies |
| Carbamazepine | NERVOUS SYSTEM | N03AF01 | HLA-B, HLA-A | Boxed Warning, Warnings, Precautions |
| Carglumic Acid | ALIMENTARY TRACT AND METABOLISM\ | A16AA05 | NAGS | Indications and Usage, Dosage and Administration, Warnings and Precautions, Use in Specific Populations, Clinical Pharmacology, Clinical Studies |
| Cariprazine | NERVOUS SYSTEM | N05AX15 | CYP2D6 | Clinical Pharmacology |
| Carisoprodol | MUSCULO-SKELETAL SYSTEM | M03BA02 | CYP2C19 | Use in Specific Populations, Clinical Pharmacology |
| Carvedilol | CARDIOVASCULAR SYSTEM | C07AG02 | CYP2D6 | Drug Interactions, Clinical Pharmacology |
| Casimersen | NERVOUS SYSTEM | - | DMD | Indications and Usage, Adverse Reactions, Use in Specific Populations, Clinical Pharmacology, Clinical Studies |
| Ceftriaxone | ANTIINFECTIVES FOR SYSTEMIC USE | J01DD04 | G6PD, Nonspecific (Congenital Methemoglobinemia) | Warnings |
| Celecoxib | ANTINEOPLASTIC AND IMMUNOMODULATING AGENTS | M01AH01 | CYP2C9 | Dosage and Administration, Use in Specific Populations, Clinical Pharmacology |
| Cemiplimab-rwlc | ANTINEOPLASTIC AND IMMUNOMODULATING AGENTS | L01XC33 | ALK, PD-L1, EGFR, ROS1 | Indications and Usage, Dosage and Administration, Clinical Studies |
| Ceritinib | ANTINEOPLASTIC AND IMMUNOMODULATING AGENTS | L01XE28 | ALK | Indications and Usage, Dosage and Administration, Warning and Precautions, Adverse Reactions, Clinical Studies |
| Cerliponase Alfa | ALIMENTARY TRACT AND METABOLISM\ | A16AB17 | TPP1 | Indications and Usage, Use in Specific Populations, Clinical Studies |
| Cetuximab | ANTINEOPLASTIC AND IMMUNOMODULATING AGENTS | L01XC06 | EGFR, RAS | Indications and Usage, Dosage and Administration, Adverse Reactions, Clinical Studies |
| Cevimeline | NERVOUS SYSTEM | N07AX03 | CYP2D6 | Precautions |
| Chloroprocaine | NERVOUS SYSTEM | N01BA04 | G6PD, Nonspecific (Congenital Methemoglobinemia) | Warnings |
| Chloroquine | ANTIPARASITIC PRODUCTS, INSECTICIDES AND REPELLENTS | P01BA02 | G6PD | Precautions, Adverse Reactions |
| Chlorpropamide | ALIMENTARY TRACT AND METABOLISM | A10BB02 | G6PD | Precautions |
| Cholic acid | ALIMENTARY TRACT AND METABOLISM | A05AA03 | AMACR, AKR1D1, CYP7A1, CYP27A1, DHCR7, HSD3B2 (Bile Acid Synthesis Disorders) | Indications and Usage, Dosage and Administration, Warnings and Precautions, Adverse Reactions, Use in Specific Populations, Clinical Studies |
| Cisplatin | ANTINEOPLASTIC AND IMMUNOMODULATING AGENTS | L01XA01 | TPMT | Adverse Reactions |
| Citalopram | NERVOUS SYSTEM | N06AB10 | CYP2C19, CYP2D6 | Dosage and Administration, Warnings, Clinical Pharmacology |
| Clobazam | NERVOUS SYSTEM | N05BA09 | CYP2C19 | Dosage and Administration, Use in Specific Populations, Clinical Pharmacology |
| Clomipramine | NERVOUS SYSTEM | N06AA04 | CYP2D6 | Precautions |
| Clopidogrel | BLOOD AND BLOOD FORMING ORGANS | B01AC04 | CYP2C19 | Boxed Warning, Warnings and Precautions, Clinical Pharmacology |
| Clozapine | NERVOUS SYSTEM | N05AH02 | CYP2D6 | Dosage and Administration, Use in Specific Populations, Clinical Pharmacology |
| Cobimetinib | ANTINEOPLASTIC AND IMMUNOMODULATING AGENTS | L01XE38 | BRAF | Indications and Usage, Dosage and Administration, Adverse Reactions, Clinical Studies |
| Codeine | RESPIRATORY SYSTEM | R05DA04 | CYP2D6 | Boxed Warning, Warnings and Precautions, Use in Specific Populations, Patient Counseling Information |
| Crizanlizumab-tmca | BLOOD AND BLOOD FORMING ORGANS | B06AX01 | HBB | Adverse Reactions, Clinical Studies |
| Crizotinib | ANTINEOPLASTIC AND IMMUNOMODULATING AGENTS | L01XE16 | ALK, ROS1 | Indications and Usage, Dosage and Administration, Adverse Reactions, Use in Specific Populations, Clinical Pharmacology, Clinical Studies |
| Dabrafenib | ANTINEOPLASTIC AND IMMUNOMODULATING AGENTS | L01XE23 | BRAF, G6PD, RAS | Indications and Usage, Dosage and Administration, Warnings and Precautions, Adverse Reactions, Clinical Pharmacology, Clinical Studies, Patient Counseling Information |
| Daclatasvir | ANTIINFECTIVES FOR SYSTEMIC USE | J05AP07 | IFNL3 (IL28B) | Clinical Studies |
| Dacomitinib | ANTINEOPLASTIC AND IMMUNOMODULATING AGENTS | L01XE47 | EGFR | Indications and Usage, Dosage and Administration, Adverse Reactions, Use in Specific Populations, Clinical Studies |
| Dapsone | ANTIINFECTIVES FOR SYSTEMIC USE | J04BA02 | G6PD, Nonspecific (Congenital Methemoglobinemia) | Warnings and Precautions, Use in Specific Populations, Patient Counseling Information, Adverse Reactions |
| Darifenacin | GENITO URINARY SYSTEM AND SEX HORMONES | G04BD10 | CYP2D6 | Clinical Pharmacology |
| Darunavir | ANTIINFECTIVES FOR SYSTEMIC USE | J05AE10 | X |  |
| Dasabuvir, Ombitasvir, Paritaprevir, and Ritonavir | ANTIINFECTIVES FOR SYSTEMIC USE | J05AP52 | IFNL3 (IL28B) | Clinical Studies |
| Dasatinib | ANTINEOPLASTIC AND IMMUNOMODULATING AGENTS | L01XE06 | BCR-ABL1 (Philadelphia chromosome) | Indications and Usage, Dosage and Administration, Warnings and Precautions, Adverse Reactions, Use in Specific Populations, Clinical Studies |
| Denileukin Diftitox | ANTINEOPLASTIC AND IMMUNOMODULATING AGENTS | L01XX29 | IL2RA (CD25 antigen) | Indications and Usage, Clinical Studies |
| Desflurane | NERVOUS SYSTEM | N01AB07 | Nonspecific (Genetic Susceptibility to Malignant Hyperthermia) | Contraindications |
| Desipramine | NERVOUS SYSTEM | N06AA01 | CYP2D6 | Precautions |
| Desvenlafaxine | NERVOUS SYSTEM | N06AX23 | CYP2D6 | Clinical Pharmacology |
| Deutetrabenazine | NERVOUS SYSTEM | - | CYP2D6 | Dosage and Administration, Warnings and Precautions, Use in Specific Populations, Clinical Pharmacology |
| Dexlansoprazole | ALIMENTARY TRACT AND METABOLISM\ | A02BC06 | CYP2C19 | Drug Interactions, Clinical Pharmacology |
| Dextromethorphan and Quinidine | NERVOUS SYSTEM | N07XX59 | CYP2D6 | Warnings and Precautions, Clinical Pharmacology |
| Diazepam | NERVOUS SYSTEM | N05BA01 | CYP2C19 | Clinical Pharmacology |
| Dinutuximab | ANTINEOPLASTIC AND IMMUNOMODULATING AGENTS | L01XC16 | MYCN | Clinical Studies |
| Divalproex sodium | NERVOUS SYSTEM | N03AG01 | POLG | Contraindications, Warnings and Precautions |
| Docetaxel | ANTINEOPLASTIC AND IMMUNOMODULATING AGENTS | L01CD02 | ESR, PGR (Hormone Receptor) | Clinical Studies |
| Dolutegravir | ANTIINFECTIVES FOR SYSTEMIC USE | J05AX12 | UGT1A1 | Clinical Pharmacology |
| Donepezil | NERVOUS SYSTEM | N06DA02 | CYP2D6 | Clinical Pharmacology |
| Dostarlimab-gxly | ANTINEOPLASTIC AND IMMUNOMODULATING AGENTS | L01XC40 | Mismatch Repair | Indication and Usage, Dosage and Administration, Adverse Reactions, Clinical Studies |
| Doxepin | NERVOUS SYSTEM | N06AA12 | CYP2D6, CYP2C19 | Clinical Pharmacology |
| Dronabinol | ALIMENTARY TRACT AND METABOLISM\ | A04AD10 | CYP2C9 | Use in Specific Populations, Clinical Pharmacology |
| Drospirenone and Ethinyl Estradiol | GENITO URINARY SYSTEM AND SEX HORMONES | G03AA12 | CYP2C19 | Clinical Pharmacology |
| Duloxetine | NERVOUS SYSTEM | N06AX21 | CYP2D6 | Drug Interactions |
| Durvalumab | ANTINEOPLASTIC AND IMMUNOMODULATING AGENTS | L01XC28 | CD274 (PD-L1) | Clinical Pharmacology, Clinical Studies |
| Duvelisib | ANTINEOPLASTIC AND IMMUNOMODULATING AGENTS | L01EM04 | Chromosome 17p | Clinical Studies |
| Eculizumab | ANTINEOPLASTIC AND IMMUNOMODULATING AGENTS | L04AA25 | ACHR, AQP4 | Indications and Usage, Clinical Studies |
| Efavirenz | ANTIINFECTIVES FOR SYSTEMIC USE | J05AG03 | CYP2B6 | Clinical Pharmacology |
| Elagolix | SYSTEMIC HORMONAL PREPARATIONS, EXCL. SEX HORMONES AND INSULINS | H01CC03 | SLCO1B1 | Clinical Pharmacology |
| Elbasvir and Grazoprevir | ANTIINFECTIVES FOR SYSTEMIC USE | J05AP54 | IFNL3 (IL28B) | Clinical Studies |
| Elexacaftor, Ivacaftor, and Tezacaftor | RESPIRATORY SYSTEM | R07AX32 | CFTR | Indications and Usage, Use in Specific Populations, Clinical Pharmacology, Clinical Studies |
| Eliglustat | ALIMENTARY TRACT AND METABOLISM\ | A16AX10 | CYP2D6 | Indications and Usage, Dosage and Administration, Contraindications, Warnings and Precautions, Drug Interactions, Use in Specific Populations, Clinical Pharmacology, Clinical Studies |
| Elosulfase | ALIMENTARY TRACT AND METABOLISM\ | A16AB12 | GALNS | Indications and Usage, Warnings and Precautions, Use in Specific Populations, Clinical Pharmacology, Clinical Studies |
| Eltrombopag | BLOOD AND BLOOD FORMING ORGANS | B02BX05 | F5 (Factor V Leiden), SERPINC1 (Antithrombin III) / Chromosome 7, Chromosome 13 | Warnings and Precautions / Adverse Reactions |
| Emapalumab-lzsg | ANTINEOPLASTIC AND IMMUNOMODULATING AGENTS | L04AA39 | PRF1, RAB27A, SH2D1A, STXBP2, STX11, UNC13D, XIAP (Hemophagocytic Lymphohistiocytosis) | Clinical Studies |
| Enasidenib | ANTINEOPLASTIC AND IMMUNOMODULATING AGENTS | L01XX59 | IDH2 | Indications and Usage, Dosage and Administration, Clinical Pharmacology, Clinical Studies |
| Encorafenib | ANTINEOPLASTIC AND IMMUNOMODULATING AGENTS | L01EC03 | BRAF, RAS | Indications and Usage, Dosage and Administration, Warnings and Precautions, Adverse Reactions, Use in Specific Populations, Clinical Pharmacology, Clinical Studies |
| Enfortumab Vedotin-ejfv | ANTINEOPLASTIC AND IMMUNOMODULATING AGENTS | N01AB04 | NECTIN4 | Clinical Studies |
| Entrectinib | ANTINEOPLASTIC AND IMMUNOMODULATING AGENTS | L01EX14 | ROS1, NTRK | Indications and Usage, Dosage and Administration, Adverse Reactions, Use in Specific Populations, Clinical Pharmacology, Clinical Studies |
| Enzalutamide | ANTINEOPLASTIC AND IMMUNOMODULATING AGENTS | L02BB | X |  |
| Erdafitinib | ANTINEOPLASTIC AND IMMUNOMODULATING AGENTS | L01EX16 | FGFR / CYP2C9 | Indications and Usage, Dosage and Administration, Adverse Reactions, Clinical Studies, Patient Counseling Information / Use in Specific Populations, Clinical Pharmacology |
| Eribulin | ANTINEOPLASTIC AND IMMUNOMODULATING AGENTS | L01XX41 | ERBB2 (HER2), ESR, PGR (Hormone Receptor) | Clinical Studies |
| Erlotinib | ANTINEOPLASTIC AND IMMUNOMODULATING AGENTS | L01XE03 | EGFR | Indications and Usage, Dosage and Administration, Adverse Reactions, Clinical Studies |
| Erythromycin and Sulfisoxazole | ANTIINFECTIVES FOR SYSTEMIC USE | J01FA01 | G6PD | Precautions |
| Escitalopram | NERVOUS SYSTEM | N06AB10 | CYP2D6, CYP2C19 | Drug Interactions |
| Esomeprazole | ALIMENTARY TRACT AND METABOLISM\ | A02BC05 | CYP2C19 | Drug Interactions, Clinical Pharmacology |
| Estradiol and Progesterone | GENITO URINARY SYSTEM AND SEX HORMONES | G03FB06 | PROC, PROS1, SERPINC1 (Antithrombin III) | Contraindications |
| Eteplirsen | MUSCULO-SKELETAL SYSTEM | M09AX06 | DMD | Indications and Usage, Adverse Reactions, Use in Specific Populations, Clinical Studies |
| Ethinyl estradiol and Norelgestromin | GENITO URINARY SYSTEM AND SEX HORMONES | G03AA13 | X |  |
| Everolimus | ANTINEOPLASTIC AND IMMUNOMODULATING AGENTS | L01XE10 | ERBB2 (HER2), ESR (Hormone Receptor) | Indications and Usage, Dosage and Administration, Warnings and Precautions, Adverse Reactions, Use in Specific Populations, Clinical Pharmacology, Clinical Studies |
| Evinacumab-dgnb | ALIMENTARY TRACT AND METABOLISM | - | LDLR, Homozygous Familial Hypercholesterolemia | Indication and Usage, Adverse Reactions, Use in Specific Populations, Clinical Pharmacology, Clinical Studies |
| Evolocumab | CARDIOVASCULAR SYSTEM | C10AX13 | X |  |
| Exemestane | ANTINEOPLASTIC AND IMMUNOMODULATING AGENTS | L02BG06 | ESR, PGR (Hormone Receptor) | Indications and Usage, Dosage and Administration, Clinical Studies |
| Fampridine | NERVOUS SYSTEM | N07XX07 | X |  |
| Fam-Trastuzumab Deruxtecan-nxki | ANTINEOPLASTIC AND IMMUNOMODULATING AGENTS | L01XC03 | ERBB2 (HER2) | Indications and Usage, Warnings and Precautions, Adverse Reactions, Use in Specific Populations, Clinical Pharmacology, Clinical Studies |
| Fesoterodine | GENITO URINARY SYSTEM AND SEX HORMONES | G04BD11 | CYP2D6 | Drug Interactions, Clinical Pharmacology |
| Flibanserin | GENITO URINARY SYSTEM AND SEX HORMONES | G02CX02 | CYP2C9, CYP2C19, CYP2D6 | Clinical Pharmacology, Adverse Reactions, Use in Specific Populations |
| Fluorouracil | ANTINEOPLASTIC AND IMMUNOMODULATING AGENTS | L01BC02 | DPYD | Contraindications, Warnings |
| Fluoxetine | NERVOUS SYSTEM | N06AB03 | CYP2D6 | Precautions, Clinical Pharmacology |
| Flurbiprofen | MUSCULO-SKELETAL SYSTEM | M02AA19 | CYP2C9 | Clinical Pharmacology |
| Flutamide | ANTINEOPLASTIC AND IMMUNOMODULATING AGENTS | L02BB01 | G6PD | Warnings |
| Fluvoxamine | NERVOUS SYSTEM | N06AB08 | CYP2D6 | Drug Interactions |
| Formoterol | RESPIRATORY SYSTEM | R03CC | CYP2D6, CYP2C19 | Clinical Pharmacology |
| Fosphenytoin | NERVOUS SYSTEM | N03AB05 | HLA-B | Warnings and Precautions |
| Fulvestrant | ANTINEOPLASTIC AND IMMUNOMODULATING AGENTS | L02BA03 | ERBB2 (HER2), ESR, PGR (Hormone Receptor) | Indications and Usage, Adverse Reactions, Clinical Studies |
| Galantamine | NERVOUS SYSTEM | N06DA04 | CYP2D6 | Clinical Pharmacology |
| Gefitinib | ANTINEOPLASTIC AND IMMUNOMODULATING AGENTS | L01XE02 | EGFR, CYP2D6 | Indications and Usage, Dosage and Administration, Clinical Studies |
| Gemtuzumab Ozogamicin | ANTINEOPLASTIC AND IMMUNOMODULATING AGENTS | L01XC05 | CD33 | Indications and Usage, Dosage and Administration, Adverse Reactions, Clinical Studies |
| Gilteritinib | ANTINEOPLASTIC AND IMMUNOMODULATING AGENTS | L01EX13 | FLT3 | Indications and Usage, Dosage and Administration, Clinical Studies |
| Givosiran | ALIMENTARY TRACT AND METABOLISM\ | A16AX16 | CPOX, HMBS, PPOX (Acute Hepatic Porphyria) | Clinical Studies |
| Glimepiride | ALIMENTARY TRACT AND METABOLISM | A10BB12 | G6PD | Warnings and Precautions, Adverse Reactions |
| Glipizide | ALIMENTARY TRACT AND METABOLISM | A10BB07 | G6PD | Precautions |
| Glyburide | ALIMENTARY TRACT AND METABOLISM | A10BB01 | G6PD | Precautions |
| Golodirsen | MUSCULO-SKELETAL SYSTEM | M09AX08 | DMD | Indications and Usage, Use in Specific Populations, Clinical Pharmacology, Clinical Studies |
| Goserelin | ANTINEOPLASTIC AND IMMUNOMODULATING AGENTS | L02AE03 | ESR, PGR (Hormone Receptor) | Indications and Usage, Clinical Studies |
| Hydralazine | CARDIOVASCULAR SYSTEM | C02DB02 | Nonspecific (NAT) | Clinical Pharmacology |
| Hydroxychloroquine | ANTIPARASITIC PRODUCTS, INSECTICIDES AND REPELLENTS | P01BA02 | G6PD | Precautions, Adverse Reactions |
| Ibritumomab | VARIOUS | V10XX02 | X |  |
| Ibrutinib | ANTINEOPLASTIC AND IMMUNOMODULATING AGENTS | L01XE27 | Chromosome 17p, Chromosome 11q, MYD88 | Indications and Usage, Clinical Studies |
| Iloperidone | NERVOUS SYSTEM | N05AX14 | CYP2D6 | Dosage and Administration, Warnings and Precautions, Drug Interactions, Clinical Pharmacology |
| Imatinib | ANTINEOPLASTIC AND IMMUNOMODULATING AGENTS | L01XE01 | KIT, BCR-ABL1 (Philadelphia chromosome), PDGFRB, FIP1L1-PDGFRA | Indications and Usage, Dosage and Administration, Clinical Studies |
| Imipramine | NERVOUS SYSTEM | N06AA02 | CYP2D6 | Precautions |
| Indacaterol | RESPIRATORY SYSTEM | R03AC18 | UGT1A1 | Clinical Pharmacology |
| Inebilizumab-cdon | ANTINEOPLASTIC AND IMMUNOMODULATING AGENTS | L04AA47 | AQP4 | Indications and Usage, Clinical Studies |
| Infigratinib | ANTINEOPLASTIC AND IMMUNOMODULATING AGENTS | - | FGFR2 | Indications and Usage, Dosage and Administration, Clinical Studies |
| Inotersen | NERVOUS SYSTEM | N07XX15 | TTR | Adverse Reactions, Clinical Pharmacology |
| Inotuzumab Ozogamicin | ANTINEOPLASTIC AND IMMUNOMODULATING AGENTS | L01XC26 | BCR-ABL1 (Philadelphia chromosome) | Clinical Studies |
| Ipilimumab | ANTINEOPLASTIC AND IMMUNOMODULATING AGENTS | L01XC11 | HLA-A, Microsatellite Instability, Mismatch Repair, CD274 (PD-L1), ALK, EGFR | Clinical Studies / Indications and Usage, Adverse Reactions, Use in Specific Populations, Clinical Studies |
| Irinotecan | ANTINEOPLASTIC AND IMMUNOMODULATING AGENTS | L01XX19 | UGT1A1 | Dosage and Administration, Warnings and Precautions, Clinical Pharmacology |
| Isatuximab- irfc | ANTINEOPLASTIC AND IMMUNOMODULATING AGENTS | L01XC38 | Chromosome 17p, Chromosome 4p;14q, Chromosome 14q;16q | Clinical Studies |
| Isoflurane | NERVOUS SYSTEM | N01AB06 | Nonspecific (Genetic Susceptibility to Malignant Hyperthermia) | Contraindications |
| Isoniazid | ANTIINFECTIVES FOR SYSTEMIC USE | J04AC01 | X |  |
| Isoniazid, Pyrazinamide, and Rifampin | ANTIINFECTIVES FOR SYSTEMIC USE | J04AM05 | Nonspecific (NAT) | Clinical Pharmacology |
| Isosorbide Dinitrate | CARDIOVASCULAR SYSTEM | C01DA08 | CYB5R | Overdosage |
| Isosorbide Mononitrate | CARDIOVASCULAR SYSTEM | C01DA14 | CYB5R | Overdosage |
| Ivacaftor | RESPIRATORY SYSTEM | R07AX02 | CFTR | Indications and Usage, Adverse Reactions, Use in Specific Populations, Clinical Pharmacology, Clinical Studies |
| Ivacaftor and Lumacaftor | RESPIRATORY SYSTEM | R07AX30 | CFTR | Indications and Usage, Adverse Reactions, Use in Specific Populations, Clinical Studies |
| Ivacaftor and Tezacaftor | RESPIRATORY SYSTEM | R07AX31 | CFTR | Indications and Usage, Adverse Reactions, Use in Specific Populations, Clinical Pharmacology, Clinical Studies |
| Ivosidenib | ANTINEOPLASTIC AND IMMUNOMODULATING AGENTS | L01XX62 | IDH1 | Indications and Usage, Dosage and Administration, Clinical Pharmacology, Clinical Studies |
| Ixabepilone | ANTINEOPLASTIC AND IMMUNOMODULATING AGENTS | L01DC04 | ERBB2 (HER2), ESR, PGR (Hormone Receptor) | Clinical Studies |
| Lacosamide | NERVOUS SYSTEM | N03AX18 | CYP2C19 | Clinical Pharmacology |
| Lansoprazole | ALIMENTARY TRACT AND METABOLISM\ | A02BC03 | CYP2C19 | Drug Interactions, Clinical Pharmacology |
| Lapatinib | ANTINEOPLASTIC AND IMMUNOMODULATING AGENTS | L01XE07 | ERBB2 (HER2), ESR, PGR (Hormone Receptor) / HLA-DQA1, HLA-DRB1 | Indications and Usage, Dosage and Administration, Adverse Reactions, Use in Specific Populations, Clinical Studies / Clinical Pharmacology |
| Larotrectinib | ANTINEOPLASTIC AND IMMUNOMODULATING AGENTS | L01EX12 | NTRK | Indications and Usage, Dosage and Administration, Adverse Reactions, Clinical Studies |
| Ledipasvir and Sofosbuvir | ANTIINFECTIVES FOR SYSTEMIC USE | J05AP51 | IFNL3 (IL28B) | Clinical Studies |
| Lenalidomide | ANTINEOPLASTIC AND IMMUNOMODULATING AGENTS | L04AX04 | Chromosome 5q | Boxed Warning, Indications and Usage, Adverse Reactions, Use in Specific Populations, Clinical Studies |
| Lenvatinib | ANTINEOPLASTIC AND IMMUNOMODULATING AGENTS | L01XE29 | Microsatellite Instability, Mismatch Repair | Indications and Usage, Adverse Reactions, Clinical Studies |
| Lesinurad | MUSCULO-SKELETAL SYSTEM | M04AB05 | CYP2C9 | Drug Interactions, Clinical Pharmacology |
| Letrozole | ANTINEOPLASTIC AND IMMUNOMODULATING AGENTS | L02BG04 | ESR, PGR (Hormone Receptor) | Indications and Usage, Adverse Reactions, Clinical Studies |
| Lidocaine and Prilocaine | NERVOUS SYSTEM | N01BB | Nonspecific (Congenital Methemoglobinemia), G6PD | Warnings and Precautions, Clinical Pharmacology |
| Lidocaine and Tetracaine | NERVOUS SYSTEM | N01BB52 | G6PD, Nonspecific (Congenital Methemoglobinemia) | Warnings and Precautions |
| Lofexidine | NERVOUS SYSTEM | N07BC04 | CYP2D6 | Use in Specific Populations |
| Lomitapide | CARDIOVASCULAR SYSTEM | C10AX12 | X |  |
| Lonafarnib | ALIMENTARY TRACT AND METABOLISM | - | LMNA, ZMPSTE24 | Indications and Usage, Adverse Reactions, Use in Specific Populations, Clinical Studies |
| Lorlatinib | ANTINEOPLASTIC AND IMMUNOMODULATING AGENTS | L01ED05 | ALK, ROS1 | Indications and Usage, Adverse Reactions, Clinical Studies |
| Lumasiran | ALIMENTARY TRACT AND METABOLISM | A16AX18 | AGXT | Indications and Usage, Adverse Reactions, Use in Specific Populations, Clinical Pharmacology, Clinical Studies |
| Luspatercept–aamt | BLOOD AND BLOOD FORMING ORGANS | B03XA06 | HBB/F2 (Prothrombin) | Clinical Studies/Warnings and Precautions |
| Lusutrombopag | BLOOD AND BLOOD FORMING ORGANS | B02BX07 | F5 (Factor V Leiden), PROC, PROS1, SERPINC1 (Antithrombin III) | Warnings and Precautions |
| Lutetium Dotatate Lu-177 | VARIOUS | V10XX04 | SSTR | Indications and Usage, Adverse Reactions, Clinical Pharmacology, Clinical Studies |
| Mafenide | DERMATOLOGICALS | D06BA03 | G6PD | Warnings, Adverse Reactions |
| Maraviroc | ANTIINFECTIVES FOR SYSTEMIC USE | J05AX09 | X |  |
| Margetuximab-cmkb | ANTINEOPLASTIC AND IMMUNOMODULATING AGENTS | - | HER2, FCGR2A, FCGR2B, FCGR3A | Indications and Usage, Adverse Reactions, Clinical Pharmacology, Clinical Studies |
| Meclizine | RESPIRATORY SYSTEM | R06AE05 | CYP2D6 | Warnings and Precautions |
| Meloxicam | MUSCULO-SKELETAL SYSTEM | M01AC06 | CYP2C9 | Use in Specific Populations, Clinical Pharmacology |
| Mepivacaine | NERVOUS SYSTEM | N01BB03 | G6PD, Nonspecific (Congenital Methemoglobinemia) | Warnings |
| Mercaptopurine | ANTINEOPLASTIC AND IMMUNOMODULATING AGENTS | L01BB02 | TPMT, NUDT15 | Dosage and Administration, Warnings and Precautions, Adverse Reactions, Clinical Pharmacology |
| Methylene Blue | VARIOUS | V03AB | G6PD | Contraindications, Warnings and Precautions |
| Metoclopramide | ALIMENTARY TRACT AND METABOLISM\ | A03FA01 | CYB5R, G6PD, CYP2D6 | Use in Specific Populations, Overdosage, Dosage and Administration, Clinical Pharmacology |
| Metoprolol | CARDIOVASCULAR SYSTEM | C07AB02 | CYP2D6 | Drug Interactions, Clinical Pharmacology |
| Metreleptin | ALIMENTARY TRACT AND METABOLISM | A16AA07 | LEP | Contraindications |
| Midostaurin | ANTINEOPLASTIC AND IMMUNOMODULATING AGENTS | L01XE39 | FLT3, NPM1, KIT | Indications and Usage, Dosage and Administration, Adverse Reactions, Clinical Studies |
| Migalastat | ALIMENTARY TRACT AND METABOLISM\ | A16AX14 | GLA | Indications and Usage, Dosage and Administration, Clinical Pharmacology, Clinical Studies |
| Mirabegron | GENITO URINARY SYSTEM AND SEX HORMONES | G04BD12 | CYP2D6 | Clinical Pharmacology |
| Mivacurium | MUSCULO-SKELETAL SYSTEM | M03AC10 | BCHE | Warnings, Precautions, Clinical Pharmacology |
| Modafinil | NERVOUS SYSTEM | N06BA07 | CYP2D6 | Clinical Pharmacology |
| Moviprep | Various | - | X |  |
| Mycophenolic Acid | ANTINEOPLASTIC AND IMMUNOMODULATING AGENTS | L04AA06 | HPRT1 | Warnings and Precautions |
| Nalidixic Acid | ANTIINFECTIVES FOR SYSTEMIC USE | J01MB02 | G6PD | Precautions, Adverse Reactions |
| Nebivolol | CARDIOVASCULAR SYSTEM | C07AB12 | CYP2D6 | Dosage and Administration, Clinical Pharmacology |
| Nefazodone | NERVOUS SYSTEM | N06AX06 | CYP2D6 | Precautions |
| Neratinib | ANTINEOPLASTIC AND IMMUNOMODULATING AGENTS | L01EH02 | ERBB2 (HER2), ESR, PGR (Hormone Receptor) | Indications and Usage, Adverse Reactions, Clinical Studies |
| Nilotinib | ANTINEOPLASTIC AND IMMUNOMODULATING AGENTS | L01XE08 | BCR-ABL1 (Philadelphia chromosome) / UGT1A1 | Indications and Usage, Dosage and Administration, Warnings and Precautions, Adverse Reactions, Use in Specific Populations, Clinical Pharmacology, Clinical Studies / Clinical Pharmacology |
| Niraparib | ANTINEOPLASTIC AND IMMUNOMODULATING AGENTS | L01XX54 | BRCA, Genomic Instability (Homologous Recombination Deficiency) | Indication and Usage, Dosage and Administration, Clinical Studies |
| Nitrofurantoin | ANTIINFECTIVES FOR SYSTEMIC USE | J01XE01 | G6PD | Warnings, Adverse Reactions |
| Nivolumab | ANTINEOPLASTIC AND IMMUNOMODULATING AGENTS | L01XC17 | BRAF, CD274 (PD-L1), Microsatellite Instability, Mismatch Repair, EGFR, ALK | Adverse Reactions, Clinical Studies |
| Norfloxacin | ANTIINFECTIVES FOR SYSTEMIC USE | J01MA06 | X |  |
| Nortriptyline | NERVOUS SYSTEM | N06AA10 | CYP2D6 | Precautions |
| Nusinersen | MUSCULO-SKELETAL SYSTEM | M09AX07 | SMN2 | Clinical Pharmacology, Clinical Studies |
| Obinutuzumab | ANTINEOPLASTIC AND IMMUNOMODULATING AGENTS | L01XC15 | MS4A1 (CD20 antigen) | Clinical Studies |
| Ofatumumab | Antineoplastic and immunomodulating agents | L01XC10 | X |  |
| Olaparib | ANTINEOPLASTIC AND IMMUNOMODULATING AGENTS | L01XX46 | BRCA, ERBB2 (HER2), ESR, PGR (Hormone Receptor), BRCA, Genomic Instability (Homologous Recombination Deficiency), Homologous Recombination Repair, PPP2R2A | Indications and Usage, Dosage and Administration, Warnings and Precautions, Adverse Reactions, Clinical Studies |
| Olaratumab | ANTINEOPLASTIC AND IMMUNOMODULATING AGENTS | L01XC27 | PDGFRA | Clinical Studies |
| Oliceridine | NERVOUS SYSTEM | - | CYP2D6 | Warnings and Precautions, Drug Interactions, Use in Specific Populations, Clinical Pharmacology |
| Omacetaxine | ANTINEOPLASTIC AND IMMUNOMODULATING AGENTS | L01XX40 | BCR-ABL1 (Philadelphia chromosome) | Clinical Studies |
| Ombitasvir, Paritaprevir, and Ritonavir | ANTIINFECTIVES FOR SYSTEMIC USE | J05AP53 | IFNL3 (IL28B) | Clinical Studies |
| Omeprazole | ALIMENTARY TRACT AND METABOLISM\ | A02BC01 | CYP2C19 | Drug Interactions, Clinical Pharmacology |
| Ondansetron | ALIMENTARY TRACT AND METABOLISM\ | A04AA01 | CYP2D6 | Clinical Pharmacology |
| Osimertinib | ANTINEOPLASTIC AND IMMUNOMODULATING AGENTS | L01XE35 | EGFR | Indications and Usage, Dosage and Administration, Adverse Reactions, Clinical Studies |
| Ospemifene | GENITO URINARY SYSTEM AND SEX HORMONES | G03XC05 | CYP2C9, CYP2B6 | Clinical Pharmacology |
| Oxcarbazepine | NERVOUS SYSTEM | N03AF02 | HLA-B | Warnings and Precautions |
| Oxymetazoline and Tetracaine | RESPIRATORY SYSTEM | R01AA05 | G6PD, Nonspecific (Congenital Methemoglobinemia) | Warnings and Precautions |
| Palbociclib | ANTINEOPLASTIC AND IMMUNOMODULATING AGENTS | L01XE33 | ESR (Hormone Receptor), ERBB2 (HER2) | Indications and Usage, Adverse Reactions, Clinical Studies |
| Paliperidone | NERVOUS SYSTEM | N05AX13 | CYP2D6 | Clinical Pharmacology |
| Palonosetron | ALIMENTARY TRACT AND METABOLISM\ | A04AA05 | CYP2D6 | Clinical Pharmacology |
| Panitumumab | ANTINEOPLASTIC AND IMMUNOMODULATING AGENTS | L01XC08 | EGFR, RAS | Adverse Reactions, Clinical Pharmacology, Clinical Studies |
| Pantoprazole | ALIMENTARY TRACT AND METABOLISM\ | A02BC02 | CYP2C19 | Clinical Pharmacology |
| Parathyroid Hormone | SYSTEMIC HORMONAL PREPARATIONS, EXCL. SEX HORMONES AND INSULINS | H05AA03 | CASR | Indications and Usage, Clinical Studies |
| Paroxetine | NERVOUS SYSTEM | N06AB05 | CYP2D6 | Drug Interactions, Clinical Pharmacology |
| Patisiran | NERVOUS SYSTEM | N07XX12 | TTR | Adverse Reactions, Clinical Pharmacology, Clinical Studies |
| Pazopanib | ANTINEOPLASTIC AND IMMUNOMODULATING AGENTS | L01XE11 | UGT1A1, HLA-B | Clinical Pharmacology |
| Peginterferon Alfa-2b | ANTINEOPLASTIC AND IMMUNOMODULATING AGENTS | L03AB10 | IFNL3 (IL28B) | Clinical Pharmacology |
| Pegloticase | MUSCULO-SKELETAL SYSTEM | M04AX02 | G6PD | Boxed Warning, Contraindications, Warnings and Precautions, Patient Counseling Information |
| Pembrolizumab | ANTINEOPLASTIC AND IMMUNOMODULATING AGENTS | L01XC18 | BRAF, CD274 (PD-L1), Microsatellite Instability, Mismatch Repair, EGFR, ALK, Tumor Mutational Burden | Adverse Reactions, Clinical Studies |
| Pemigatinib | ANTINEOPLASTIC AND IMMUNOMODULATING AGENTS | L01EX20 | FGFR2 | Indication and Usage, Dosage and Administration, Clinical Studies |
| Perphenazine | NERVOUS SYSTEM | N05AB03 | CYP2D6 | Precautions, Clinical Pharmacology |
| Pertuzumab | ANTINEOPLASTIC AND IMMUNOMODULATING AGENTS | L01XC13 | ERBB2 (HER2), ESR, PGR (Hormone Receptor) | Indications and Usage, Dosage and Administration, Warnings and Precautions, Adverse Reactions, Clinical Pharmacology, Clinical Studies |
| Phenytoin | NERVOUS SYSTEM | N03AB02 | CYP2C9, CYP2C19 / HLA-B | Clinical Pharmacology / Warnings |
| Pimozide | NERVOUS SYSTEM | N05AG02 | CYP2D6 | Dosage and Administration, Precautions |
| Piroxicam | MUSCULO-SKELETAL SYSTEM | M01AC01 | CYP2C9 | Clinical Pharmacology |
| Pitolisant | NERVOUS SYSTEM | N07XX11 | CYP2D6 | Dosage and Administration, Use in Specific Populations, Clinical Pharmacology |
| Ponatinib | ANTINEOPLASTIC AND IMMUNOMODULATING AGENTS | L01XE24 | BCR-ABL1 (Philadelphia chromosome) | Indications and Usage, Warnings and Precautions, Adverse Reactions, Use in Specific Populations, Clinical Studies |
| Pralsetinib | ANTINEOPLASTIC AND IMMUNOMODULATING AGENTS | - | CCDC6-RET, KIF5B-RET,  RET | Indications and Usage, Dosage and Administration, Adverse Reactions, Use in Specific Populations, Clinical Pharmacology, Clinical Studies |
| Prasugrel | BLOOD AND BLOOD FORMING ORGANS | B01AC22 | CYP2C19, CYP2C9, CYP3A5, CYP2B6 | Use in Specific Populations, Clinical Pharmacology, Clinical Studies |
| Primaquine | ANTIPARASITIC PRODUCTS, INSECTICIDES AND REPELLENTS | P01BA03 | G6PD, CYB5R | Contraindications, Warnings, Precautions, Adverse Reactions, Overdosage |
| Probenecid | MUSCULO-SKELETAL SYSTEM | M04AB01 | G6PD | Adverse Reactions |
| Procainamide | CARDIOVASCULAR SYSTEM | C01BA02 | Nonspecific (NAT) | Adverse Reactions, Clinical Pharmacology |
| Propafenone | CARDIOVASCULAR SYSTEM | C01BC03 | CYP2D6 | Dosage and Administration, Warnings and Precautions, Drug Interactions, Clinical Pharmacology |
| Propranolol | CARDIOVASCULAR SYSTEM | C07AA05 | CYP2D6 | Clinical Pharmacology |
| Protriptyline | NERVOUS SYSTEM | N06AA11 | CYP2D6 | Precautions |
| Quinidine | CARDIOVASCULAR SYSTEM | C01BA01 | CYP2D6 | Precautions |
| Quinine Sulfate | ANTIPARASITIC PRODUCTS, INSECTICIDES AND REPELLENTS | P01BC01 | G6PD / CYP2D6 | Warnings and Precautions / Drug Interactions |
| Rabeprazole | ALIMENTARY TRACT AND METABOLISM\ | A02BC04 | CYP2C19 | Drug Interactions, Clinical Pharmacology |
| Raloxifene | GENITO URINARY SYSTEM AND SEX HORMONES | G03XC01 | ESR (Hormone Receptor) | Clinical Studies |
| Raltegravir | ANTIINFECTIVES FOR SYSTEMIC USE | J05AX08 | UGT1A1 | Clinical Pharmacology |
| Ramucirumab | ANTINEOPLASTIC AND IMMUNOMODULATING AGENTS | L01XC21 | EGFR, RAS | Indications and Usage, Dosage and Administration, Adverse Reactions, Clinical Studies |
| Ranolazine | CARDIOVASCULAR SYSTEM | C01EB18 | X |  |
| Rasburicase | VARIOUS | V03AF07 | G6PD, CYB5R | Boxed Warning, Contraindications, Warnings and Precautions |
| Regorafenib | ANTINEOPLASTIC AND IMMUNOMODULATING AGENTS | L01XE21 | RAS | Indications and Usage, Clinical Studies |
| Ribociclib | ANTINEOPLASTIC AND IMMUNOMODULATING AGENTS | L01XE42 | ESR, PGR (Hormone Receptor), ERBB2 (HER2) | Indications and Usage, Adverse Reactions, Clinical Studies |
| Rimegepant | NERVOUS SYSTEM | - | CYP2C9 | Clinical Pharmacology |
| Risdiplam | MUSCULO-SKELETAL SYSTEM | M09AX10 | SMN1, SMN2 | Clinical Studies |
| Risperidone | NERVOUS SYSTEM | N05AX08 | CYP2D6 | Clinical Pharmacology |
| Rituximab | ANTINEOPLASTIC AND IMMUNOMODULATING AGENTS | L01XC02 | MS4A1 (CD20 antigen) | Indications and Usage, Dosage and Administration, Adverse Reactions, Use in Specific Populations, Clinical Studies |
| Rivaroxaban | BLOOD AND BLOOD FORMING ORGANS | B01AF01 | F5 (Factor V Leiden) | Clinical Studies |
| Ropivacaine | NERVOUS SYSTEM | N01BB09 | G6PD, Nonspecific (Congenital Methemoglobinemia) | Warnings |
| Rosuvastatin | CARDIOVASCULAR SYSTEM | C10AA07 | SLCO1B1 | Clinical Pharmacology |
| Rucaparib | ANTINEOPLASTIC AND IMMUNOMODULATING AGENTS | L01XK03 | BRCA, CYP2D6, CYP1A2, BRCA, Loss of Heterozygosity (Homologous Recombination Deficiency) | Indications and Usage, Dosage and Administration, Adverse Reactions, Clinical Studies |
| Ruxolitinib | ANTINEOPLASTIC AND IMMUNOMODULATING AGENTS | L01XE18 | X |  |
| Sacituzumab Govitecan-hziy | ANTINEOPLASTIC AND IMMUNOMODULATING AGENTS | L01 | UGT1A1 | Warnings and Precautions, Clinical Pharmacology |
| Satralizumab-mwge | ANTINEOPLASTIC AND IMMUNOMODULATING AGENTS | L04AC19 | AQP4 | Indications and Usage, Adverse Reactions, Clinical Studies |
| Selpercatinib | ANTINEOPLASTIC AND IMMUNOMODULATING AGENTS | L01 | RET | Indications and Usage, Dosage and Administration, Adverse Reactions, Use in Specific Populations, Clinical Studies |
| Setmelanotide | ALIMENTARY TRACT AND METABOLISM | - | LEPR, PCSK1, POMC | Indications and Usage, Dosage and Administration, Adverse Reactions, Clinical Studies |
| Sevoflurane | NERVOUS SYSTEM | N01AB08 | RYR1 | Warnings |
| Sildenafil | GENITO URINARY SYSTEM AND SEX HORMONES | G04BE03 | X |  |
| Simeprevir | ANTIINFECTIVES FOR SYSTEMIC USE | J05AP05 | IFNL3 (IL28B) | Clinical Pharmacology, Clinical Studies |
| Siponimod | ANTINEOPLASTIC AND IMMUNOMODULATING AGENTS | L04AA42 | CYP2C9 | Dosage and Administration, Contraindications, Drug Interactions, Use in Specific Populations, Clinical Pharmacology |
| Sodium Nitrite | VARIOUS | V03AB08 | G6PD, Nonspecific (Congenital Methemoglobinemia) | Warnings and Precautions, Boxed Warning, Warnings and Precautions |
| Sodium Oxybate | NERVOUS SYSTEM | N01AX11 | ALDH5A1 (Succinic Semialdehyde Dehydrogenase Deficiency) | Contraindications |
| Sodium Phenylbutyrate | ALIMENTARY TRACT AND METABOLISM\ | A16AX03 | ASS1, CPS1, OTC (Urea Cycle Disorders) | Indications and Usage, Dosage and Administration |
| Sofosbuvir | ANTIINFECTIVES FOR SYSTEMIC USE | J05AP08 | IFNL3 (IL28B) | Clinical Studies |
| Sofosbuvir and Velpatasvir | ANTIINFECTIVES FOR SYSTEMIC USE | J05AP55 | IFNL3 (IL28B) | Clinical Studies |
| Sofosbuvir, Velpatasvir, and Voxilaprevir | ANTIINFECTIVES FOR SYSTEMIC USE | J05AP56 | IFNL3 (IL28B) | Clinical Studies |
| Sotorasib | ANTINEOPLASTIC AND IMMUNOMODULATING AGENTS | - | KRAS | Indication and Usage, Dosage and Administration, Adverse Reactions, Clinical Pharmacology, Clinical Studies |
| Succimer | VARIOUS | V09CA02 | G6PD | Clinical Pharmacology |
| Succinylcholine | MUSCULO-SKELETAL SYSTEM | M03AB01 | BCHE | Warnings, Precautions |
| Sulfadiazine | DERMATOLOGICALS | D06BA01 | G6PD | Warnings |
| Sulfamethoxazole and Trimethoprim | ANTIINFECTIVES FOR SYSTEMIC USE | J01EE01 | G6PD, Nonspecific (NAT) | Precautions |
| Sulfasalazine | ALIMENTARY TRACT AND METABOLISM\ | A07EC01 | G6PD, Nonspecific (NAT) | Precautions, Clinical Pharmacology |
| Synthetic Conjugated Estrogens, A | GENITO URINARY SYSTEM AND SEX HORMONES | - | PROC, PROS1, SERPINC1 (Antithrombin III) | Contraindications |
| Tafamidis | NERVOUS SYSTEM | N07XX08 | TTR | Clinical Pharmacology, Clinical Studies |
| Tafenoquine | ANTIINFECTIVES FOR SYSTEMIC USE | P01BA07 | G6PD | Dosage and Administration, Contraindications, Warnings and Precautions, Use in Specific Populations, Patient Counseling Information |
| Talazoparib | ANTINEOPLASTIC AND IMMUNOMODULATING AGENTS | L01XK04 | BRCA, ERBB2 (HER2) | Indications and Usage, Dosage and Administration, Adverse Reactions, Clinical Studies |
| Tamoxifen | ANTINEOPLASTIC AND IMMUNOMODULATING AGENTS | L02BA01 | ESR, PGR (Hormone Receptor), F5 (Factor V Leiden), F2 (Prothrombin), CYP2D6 | Indications and Usage, Adverse Reactions, Clinical Pharmacology, Clinical Studies |
| Tamsulosin | GENITO URINARY SYSTEM AND SEX HORMONES | G04CA02 | CYP2D6 | Warnings and Precautions, Adverse Interactions, Clinical Pharmacology |
| Telaprevir | ANTIINFECTIVES FOR SYSTEMIC USE | J05AP02 | IFNL3 (IL28B) | Clinical Pharmacology, Clinical Studies |
| Tepotinib | ANTINEOPLASTIC AND IMMUNOMODULATING AGENTS | L01EX21 | MET/ALK, EGFR | Indications and Usage, Dosage and Administration, Adverse Reactions, Use in Specific Populations/Clinical Studies |
| Tetrabenazine | NERVOUS SYSTEM | N07XX06 | CYP2D6 | Dosage and Administration, Warnings and Precautions, Use in Specific Populations, Clinical Pharmacology |
| Thioguanine | ANTINEOPLASTIC AND IMMUNOMODULATING AGENTS | L01BB03 | TPMT, NUDT15 | Dosage and Administration, Warnings, Precautions, Clinical Pharmacology |
| Thioridazine | NERVOUS SYSTEM | N05AC02 | CYP2D6 | Contraindications, Warnings, Precautions |
| Ticagrelor | BLOOD AND BLOOD FORMING ORGANS | B01AC24 | CYP2C19 | Clinical Pharmacology |
| Tipiracil and Trifluridine | ANTINEOPLASTIC AND IMMUNOMODULATING AGENTS | L01BC59 | ERBB2 (HER2), RAS | Indications and Usage, Adverse Reactions, Clinical Studies |
| Tolazamide | ALIMENTARY TRACT AND METABOLISM | A10BB05 | G6PD | Precautions |
| Tolbutamide | ALIMENTARY TRACT AND METABOLISM | A10BB03 | G6PD | Precautions |
| Tolterodine | GENITO URINARY SYSTEM AND SEX HORMONES | G04BD07 | CYP2D6 | Warnings and Precautions, Drug Interactions, Clinical Pharmacology |
| Toremifene | ANTINEOPLASTIC AND IMMUNOMODULATING AGENTS | L02BA02 | ESR (Hormone Receptor) | Indications and Usage, Clinical Studies |
| Tramadol | NERVOUS SYSTEM | N02AX02 | CYP2D6 | Boxed Warning, Warnings and Precautions, Use in Specific Populations, Clinical Pharmacology, Patient Counseling Information |
| Trametinib | ANTINEOPLASTIC AND IMMUNOMODULATING AGENTS | L01XE25 | BRAF, G6PDRAS | Indications and Usage, Dosage and Administration, Adverse Reactions, Clinical Pharmacology, Clinical Studies |
| Trastuzumab | ANTINEOPLASTIC AND IMMUNOMODULATING AGENTS | L01XC03 | ERBB2 (HER2), ESR, PGR (Hormone Receptor) | Indications and Usage, Dosage and Administration, Clinical Pharmacology, Clinical Studies |
| Tretinoin | ANTINEOPLASTIC AND IMMUNOMODULATING AGENTS | L01XX14 | PML-RARA | Indications and Usage, Warnings, Clinical Pharmacology |
| Triheptanoin | ALIMENTARY TRACT AND METABOLISM\ | A16AX17 | ACADVL, CPT2, HADHA, HADHB (Long-Chain Fatty Acid Oxidation Disorders) | Indications and Usage, Clinical Studies |
| Trimipramine | NERVOUS SYSTEM | N06AA06 | CYP2D6 | Precautions |
| Tucatinib | ANTINEOPLASTIC AND IMMUNOMODULATING AGENTS | L01EH03 | ERBB2 (HER2) | Indications and Usage, Adverse Reactions, Clinical Studies |
| Umeclidinium | RESPIRATORY SYSTEM | R03AL03 | CYP2D6 | Clinical Pharmacology |
| Upadacitinib | ANTINEOPLASTIC AND IMMUNOMODULATING AGENTS | L04AA44 | CYP2D6 | Clinical Pharmacology |
| Ustekinumab | ANTINEOPLASTIC AND IMMUNOMODULATING AGENTS | L04AC05 | IL12A, IL12B, IL23A | Warnings and Precautions |
| Valbenazine | NERVOUS SYSTEM | N07XX13 | CYP2D6 | Dosage and Administration, Warnings and Precautions, Use in Specific Populations, Clinical Pharmacology |
| Valproic Acid | NERVOUS SYSTEM | N03AG01 | POLG, Nonspecific (Urea Cycle Disorders) | Boxed Warning, Contraindications, Warnings and Precautions |
| Vandetanib | ANTINEOPLASTIC AND IMMUNOMODULATING AGENTS | L01XE12 | X |  |
| Velaglucerase alfa | ALIMENTARY TRACT AND METABOLISM\ | A16AB10 | X |  |
| Vemurafenib | ANTINEOPLASTIC AND IMMUNOMODULATING AGENTS | L01XE15 | BRAF, RAS | Indications and Usage, Dosage and Administration, Warnings and Precautions, Adverse Reactions, Use in Specific Populations, Clinical Pharmacology, Clinical Studies, Patient Counseling Information |
| Venetoclax | ANTINEOPLASTIC AND IMMUNOMODULATING AGENTS | L01XX52 | Chromosome 17p, Chromosome 11q, TP53, IDH1, IDH2, IGH, NPM1, FLT3 | Clinical Studies |
| Venlafaxine | NERVOUS SYSTEM | N06AX16 | CYP2D6 | Drug Interactions, Use in Specific Populations, Clinical Pharmacology |
| Viloxazine | NERVOUS SYSTEM | N06AX09 | CYP2D6, SLCO1B1 | Clinical Pharmacology |
| Viltolarsen | NERVOUS SYSTEM | - | DMD | Indications and Usage, Adverse Reactions, Use in Specific Populations, Clinical Pharmacology, Clinical Studies |
| Vincristine | ANTINEOPLASTIC AND IMMUNOMODULATING AGENTS | L01CA02 | BCR-ABL1 (Philadelphia chromosome) | Indications and Usage, Adverse Reactions, Clinical Studies |
| Vitamin C | ALIMENTARY TRACT AND METABOLISM | A11GA01 | X |  |
| Voriconazole | NERVOUS SYSTEM | J02AC03 | CYP2C19 | Clinical Pharmacology |
| Vortioxetine | NERVOUS SYSTEM | N06AX26 | CYP2D6 | Dosage and Administration, Clinical Pharmacology |
| Voxelotor | BLOOD AND BLOOD FORMING ORGANS | - | HBB | Clinical Pharmacology, Clinical Studies |
| Warfarin | BLOOD AND BLOOD FORMING ORGANS | B01AA03 | CYP2C9, VKORC1/PROS1, PROC | Dosage and Administration, Drug Interactions, Clinical Pharmacology/Warnings and Precautions |

**S4 Table.** Contents of drug labeling in Europe

| **Drug** | **Therapeutic Area** | **ATC code** | **Biomarker** | **Labelling Section** |
| --- | --- | --- | --- | --- |
| Abacavir | ANTIINFECTIVES FOR SYSTEMIC USE | J05AF06 | HLA-B(type 5701) | Indications, Warnings and precautions for use |
| Abemaciclib | ANTINEOPLASTIC AND IMMUNOMODULATING AGENTS | L01EF03 | HR, HER2 | Indications, Pharmacodynamic properties |
| Ado-Trastuzumab Emtansine | ANTINEOPLASTIC AND IMMUNOMODULATING AGENTS | L01XC14 | HER2 (human epidermal growth factor) | Indications, Posology and method of administration, Pharmacodynamic properties, Pharmacokinetic properties |
| Aducanumab-avwa | NERVOUS SYSTEM | - | - | - |
| Afatinib | ANTINEOPLASTIC AND IMMUNOMODULATING AGENTS | L01XE13 | EGFR | Indications, Posology and method of administration, Special warnings and precautions for use, Undesirable effects, Pharmacodynamic properties |
| Alectinib | ANTINEOPLASTIC AND IMMUNOMODULATING AGENTS | L01XE36 | ALK | Indications, Posology and method of administration, Undesirable effects, Pharmacodynamic properties, Pharmacokinetic properties |
| Alglucosidase Alfa | ALIMENTARY TRACT AND METABOLISM\ | A16AB07 | alpha-glucosidase(GAA) | Therapeutic indications, warnings and precautions for use, Pharmacodynamic properties |
| Alirocumab | CARDIOVASCULAR SYSTEM | C10AX14 | PCSK9 | Interaction, Pharmacodynamic properties, Pharmacokinetic properties |
| Aliskiren | CARDIOVASCULAR SYSTEM | C09XA02 | P-gp(MDR1)(ABCB1) | Contraindications, Special warnings and precautions for use, Interaction, Pharmacokinetic properties, Preclinical safety data |
| Allopurinol | MUSCULO-SKELETAL SYSTEM | M04AA01 | X | X |
| Alpelisib | ANTINEOPLASTIC AND IMMUNOMODULATING AGENTS | L01EM03 | HR, HER2, PIK3CA | Indications, Posology and method of administration, Pharmacodynamic properties |
| Amifampridine | NERVOUS SYSTEM | N07XX05 | NAT2 | Pharmacokinetic properties |
| Amitriptyline | NERVOUS SYSTEM | N06AA09 | CYP2D6 | Posology and method of administration, interaction, pharmacokinetic properties |
| Amivantamab-vmjw | ANTINEOPLASTIC AND IMMUNOMODULATING AGENTS | L01FX18 | - | - |
| Amoxapine | NERVOUS SYSTEM | N06AA17 | X | X |
| Amphetamine | NERVOUS SYSTEM | N06BA01 | - | - |
| Anakinra | ANTINEOPLASTIC AND IMMUNOMODULATING AGENTS | L04AC03 | CIAS1/NLRP3 | Pharmacodynamic properties |
| Anastrozole | ANTINEOPLASTIC AND IMMUNOMODULATING AGENTS | L02BG03 | hormone receptor | Indications, Posology and method of administration, Pharmacodynamic properties |
| Arformoterol | RESPIRATORY SYSTEM | R03 | - | - |
| Aripiprazole | NERVOUS SYSTEM | N05AX12 | CYP2D6 | Posology and method of administration, interaction, pharmacokinetic properties |
| Arsenic Trioxide | ANTINEOPLASTIC AND IMMUNOMODULATING AGENTS | L01XX27 | promyelocytic leukaemia/retinoic-acid-receptor-alpha (PML/RAR-alpha) gene | Indications, Pharmacodynamic properties |
| Articaine and Epinephrine | NERVOUS SYSTEM | N01BB58 | X | X |
| Ascorbic Acid, PEG-3350, Potassium Chloride, Sodium Ascorbate, Sodium Chloride, and Sodium Sulfate | ALIMENTARY TRACT AND METABOLISM\ | A06AD65 | X | X |
| Atazanavir | ANTIINFECTIVES FOR SYSTEMIC USE | J05AE08 | CYP2C19 | Warnings and precautions for use, Interaction |
| Atezolizumab | ANTINEOPLASTIC AND IMMUNOMODULATING AGENTS | L01XC32 | PD-L1, EGFR, ALK | Indications, Posology and method of administration, pregnancy, Pharmacodynamic properties, Pharmacokinetic properties, preclinical safety data, warnings and precautions for use |
| Atomoxetine | NERVOUS SYSTEM | N06BA09 | X | X |
| Atorvastatin | CARDIOVASCULAR SYSTEM | C10AA05 | X | X |
| Avapritinib | ANTINEOPLASTIC AND IMMUNOMODULATING AGENTS | L01EX18 | platelet-derived growth factor receptor alpha (PDGFRA) | Indications, Posology and method of administration, Pharmacodynamic properties |
| Avatrombopag | BLOOD AND BLOOD FORMING ORGANS | B02BX08 | Prothrombin(1), Factor V Leiden(2), CYP2C9(3) | Posology and method of administration, warnings and precautions for use, Interaction, pharmacokinetic properties |
| Avelumab | ANTINEOPLASTIC AND IMMUNOMODULATING AGENTS | L01XC31 | PD-L1 | pregnancy, Pharmacodynamic properties, pharmacokinetic properties, Preclinical safety data |
| Axitinib | ANTINEOPLASTIC AND IMMUNOMODULATING AGENTS | L01XE17 | CYP2C19, UGT1A1 | Interaction, pharmacokinetic properties |
| Azathioprine | ANTINEOPLASTIC AND IMMUNOMODULATING AGENTS | L04AX01 | X | X |
| Belimumab | ANTINEOPLASTIC AND IMMUNOMODULATING AGENTS | L04AA26 | BLyS (TNFSF13B, BAFF) | Pharmacodynamic properties |
| Belinostat | ANTINEOPLASTIC AND IMMUNOMODULATING AGENTS | L01XH04 | X | X |
| Binimetinib | ANTINEOPLASTIC AND IMMUNOMODULATING AGENTS | L01EE03 | BRAF | Indications, warnings and precautions for use, Undesirable effects, Pharmacodynamic properties |
| Blinatumomab | ANTINEOPLASTIC AND IMMUNOMODULATING AGENTS | L01XC19 | CD19, Philadelphia chromosome, MRD | Indications, Posology and method of administration, Undesirable effects, Pharmacodynamic properties, Pharmacokinetic properties |
| Boceprevir | ANTIINFECTIVES FOR SYSTEMIC USE | J05AP03 | - | - |
| Bosutinib | ANTINEOPLASTIC AND IMMUNOMODULATING AGENTS | - | BCR-ABL,  Philadelphia chromosome | Posology and method of administration, warnings and precautions for use, Undesirable effects, Pharmacodynamic properties, indications |
| Brentuximab Vedotin | ANTINEOPLASTIC AND IMMUNOMODULATING AGENTS | L01XC12 | CD30 / TNFRSF8 | Indications, warnings and precautions for use, Undesirable effects, Pharmacodynamic properties |
| Brexpiprazole | NERVOUS SYSTEM | N05AX16 | CYP2D6 | Posology and method of administration, interaction, pharmacokinetic properties |
| Brigatinib | ANTINEOPLASTIC AND IMMUNOMODULATING AGENTS | L01EA04 | ALK (anaplastic lymphoma kinase) | Indications, Posology and method of administration, Undesirable effects, Pharmacodynamic properties, Pharmacokinetic properties |
| Brivaracetam | NERVOUS SYSTEM | N03AX23 | CYP2C19 | Interaction, pharmacokinetic properties |
| Bupivacaine | NERVOUS SYSTEM | N01BB01 | X | X |
| Bupropion | NERVOUS SYSTEM | N06AX12 | X | X |
| Busulfan | ANTINEOPLASTIC AND IMMUNOMODULATING AGENTS | L01AB01 | X | X |
| Cabotegravir and Rilpivirine | ANTIINFECTIVES FOR SYSTEMIC USE | J05AJ04 | - | - |
| Cabozantinib | ANTINEOPLASTIC AND IMMUNOMODULATING AGENTS | L01XE26 | RET | Indications, Pharmacodynamic properties |
| Capecitabine | ANTINEOPLASTIC AND IMMUNOMODULATING AGENTS | L01BC06 | DPD | Contraindications, warnings and precautions for use, Pharmacokinetic properties |
| Capmatinib | ANTINEOPLASTIC AND IMMUNOMODULATING AGENTS | L01EX17 | X | X |
| Carbamazepine | NERVOUS SYSTEM | N03AF01 | X | X |
| Carglumic Acid | ALIMENTARY TRACT AND METABOLISM\ | A16AA05 | N-acetylglutamate synthase | Indications, Posology and method of administration, Undesirable effects, Pharmacodynamic properties |
| Cariprazine | NERVOUS SYSTEM | N05AX15 | X | X |
| Carisoprodol | MUSCULO-SKELETAL SYSTEM | M03BA02 | X | X |
| Carvedilol | CARDIOVASCULAR SYSTEM | C07AG02 | X | X |
| Casimersen | NERVOUS SYSTEM | - | - | - |
| Ceftriaxone | ANTIINFECTIVES FOR SYSTEMIC USE | J01DD04 | X | X |
| Celecoxib | ANTINEOPLASTIC AND IMMUNOMODULATING AGENTS | M01AH01 | X | X |
| Cemiplimab-rwlc | ANTINEOPLASTIC AND IMMUNOMODULATING AGENTS | L01XC33 | PD-L1, EGFR, ALK, ROS1 | Therapeutic indications, Posology and method of administration |
| Ceritinib | ANTINEOPLASTIC AND IMMUNOMODULATING AGENTS | L01XE28 | ALK | Indications, posology and method of administration, Undesirable effects, Pharmacodynamic properties |
| Cerliponase Alfa | ALIMENTARY TRACT AND METABOLISM\ | A16AB17 | TPP1 | Indications, pharmacodynamic properties |
| Cetuximab | ANTINEOPLASTIC AND IMMUNOMODULATING AGENTS | L01XC06 | EGFR(1), RAS(2) | Indications, pregnancy, Pharmacodynamic properties, Preclinical safety data, Posology and method of administration, Contraindications, Special warnings and precautions for use |
| Cevimeline | NERVOUS SYSTEM | N07AX03 | - | - |
| Chloroprocaine | NERVOUS SYSTEM | N01BA04 | - | - |
| Chloroquine | ANTIPARASITIC PRODUCTS, INSECTICIDES AND REPELLENTS | P01BA02 | X | X |
| Chlorpropamide | ALIMENTARY TRACT AND METABOLISM | A10BB02 | - | - |
| Cholic acid | ALIMENTARY TRACT AND METABOLISM | A05AA03 | reductase | Indications, Posology and method of administration, interaction, Pharmacodynamic properties |
| Cisplatin | ANTINEOPLASTIC AND IMMUNOMODULATING AGENTS | L01XA01 | X | X |
| Citalopram | NERVOUS SYSTEM | N06AB10 | X | X |
| Clobazam | NERVOUS SYSTEM | N05BA09 | X | X |
| Clomipramine | NERVOUS SYSTEM | N06AA04 | - | - |
| Clopidogrel | BLOOD AND BLOOD FORMING ORGANS | B01AC04 | CYP2C19 | Warnings and precautions for use, Interaction, Pharmacokinetic properties |
| Clozapine | NERVOUS SYSTEM | N05AH02 | X | X |
| Cobimetinib | ANTINEOPLASTIC AND IMMUNOMODULATING AGENTS | L01XE38 | BRAF | Indications, Posology and method of administration, Warnings and precautions for use, Undesirable effects, Pharmacodynamic properties |
| Codeine | RESPIRATORY SYSTEM | R05DA04 | CYP2D6 | Contraindications, Warnings and precautions for use, Fertility/pregnancy and lactation |
| Crizanlizumab-tmca | BLOOD AND BLOOD FORMING ORGANS | B06AX01 | P-selectin | Pharmacodynamic properties |
| Crizotinib | ANTINEOPLASTIC AND IMMUNOMODULATING AGENTS | L01XE16 | ALK, ROS1 | Indications, Posology and method of administration, Warnings and precautions for use, Undesirable effects, Pharmacodynamic properties, pharmacokinetic properties |
| Dabrafenib | ANTINEOPLASTIC AND IMMUNOMODULATING AGENTS | L01XE23 | BRAF | indications, posology and method of administration, warnings and precautions for use, Undesirable effects, Pharmacodynamic properties |
| Daclatasvir | ANTIINFECTIVES FOR SYSTEMIC USE | J05AP07 | - | - |
| Dacomitinib | ANTINEOPLASTIC AND IMMUNOMODULATING AGENTS | L01XE47 | EGFR | Indications, Posology and method of administration, Warnings and precautions for use, Pharmacodynamic properties |
| Dapsone | ANTIINFECTIVES FOR SYSTEMIC USE | J04BA02 | - | - |
| Darifenacin | GENITO URINARY SYSTEM AND SEX HORMONES | G04BD10 | CYP2D6 | Posology and method of administration, interaction, Pharmacokinetic properties |
| Darunavir | ANTIINFECTIVES FOR SYSTEMIC USE | J05AE10 | X | X |
| Dasabuvir, Ombitasvir, Paritaprevir, and Ritonavir | ANTIINFECTIVES FOR SYSTEMIC USE | J05AP52 | X | X |
| Dasatinib | ANTINEOPLASTIC AND IMMUNOMODULATING AGENTS | L01XE06 | BCR-ABL Philadelphia chromosome | Indications, Warnings and precautions for use, Undesirable effects, Pharmacodynamic properties |
| Denileukin Diftitox | ANTINEOPLASTIC AND IMMUNOMODULATING AGENTS | L01XX29 | X | X |
| Desflurane | NERVOUS SYSTEM | N01AB07 | X | X |
| Desipramine | NERVOUS SYSTEM | N06AA01 | - | - |
| Desvenlafaxine | NERVOUS SYSTEM | N06AX23 | - | - |
| Deutetrabenazine | NERVOUS SYSTEM | - | - | - |
| Dexlansoprazole | ALIMENTARY TRACT AND METABOLISM\ | A02BC06 | X | X |
| Dextromethorphan and Quinidine | NERVOUS SYSTEM | N07XX59 | - | - |
| Diazepam | NERVOUS SYSTEM | N05BA01 | - | - |
| Dinutuximab | ANTINEOPLASTIC AND IMMUNOMODULATING AGENTS | L01XC16 | - | - |
| Divalproex sodium | NERVOUS SYSTEM | N03AG01 | - | - |
| Docetaxel | ANTINEOPLASTIC AND IMMUNOMODULATING AGENTS | L01CD02 | HER2 | Indications, Pharmacodynamic properties |
| Dolutegravir | ANTIINFECTIVES FOR SYSTEMIC USE | J05AX12 | UGT1A1 | Interaction, Pharmacokinetic properties |
| Donepezil | NERVOUS SYSTEM | N06DA02 | X | X |
| Dostarlimab-gxly | ANTINEOPLASTIC AND IMMUNOMODULATING AGENTS | L01XC40 | mismatch repair deficient (dMMR), microsatellite instability-high (MSI-H) | Therapeutic indications, Posology and method of administration |
| Doxepin | NERVOUS SYSTEM | N06AA12 | - | - |
| Dronabinol | ALIMENTARY TRACT AND METABOLISM\ | A04AD10 | X | X |
| Drospirenone and Ethinyl Estradiol | GENITO URINARY SYSTEM AND SEX HORMONES | G03AA12 | X | X |
| Duloxetine | NERVOUS SYSTEM | N06AX21 | CYP2D6 | Interaction, Pharmacokinetic properties |
| Durvalumab | ANTINEOPLASTIC AND IMMUNOMODULATING AGENTS | L01XC28 | PD-L1 | Indications, Posology and method of administration, Pregnancy, Pharmacodynamic properties, Pharmacokinetic properties, Preclinical safety data |
| Duvelisib | ANTINEOPLASTIC AND IMMUNOMODULATING AGENTS | L01EM04 | X | X |
| Eculizumab | ANTINEOPLASTIC AND IMMUNOMODULATING AGENTS | L04AA25 | AChR, AQP4 | Indications, Pharmacodynamic properties |
| Efavirenz | ANTIINFECTIVES FOR SYSTEMIC USE | J05AG03 | CYP2B6 | Warnings and precautions for use, Interaction, Pharmacodynamic properties, Pharmacokinetic properties |
| Elagolix | SYSTEMIC HORMONAL PREPARATIONS, EXCL. SEX HORMONES AND INSULINS | H01CC03 | X | X |
| Elbasvir and Grazoprevir | ANTIINFECTIVES FOR SYSTEMIC USE | J05AP54 | IL28B | Pharmacodynamic properties |
| Elexacaftor, Ivacaftor, and Tezacaftor | RESPIRATORY SYSTEM | R07AX32 | CFTR(1) / CYP3A(2) | Indications, Pharmacodynamic properties, Posology and method of administration, Warnings and precautions for use, Interaction, Pharmacokinetic properties |
| Eliglustat | ALIMENTARY TRACT AND METABOLISM\ | A16AX10 | CYP2D6 | Indications, Posology and method of administration, Contraindications, Warnings and precautions for use, Interaction, Pharmacodynamic properties, Pharmacokinetic properties |
| Elosulfase | ALIMENTARY TRACT AND METABOLISM\ | A16AB12 | GALNS(N-acetylgalactosamine-6-sulfatase) | Pharmacodynamic properties |
| Eltrombopag | BLOOD AND BLOOD FORMING ORGANS | B02BX05 | Chromosome 7 / Factor V, ATIII | Posology and method of administration, Warnings and precautions for use, Undesirable effects |
| Emapalumab-lzsg | ANTINEOPLASTIC AND IMMUNOMODULATING AGENTS | L04AA39 | X | X |
| Enasidenib | ANTINEOPLASTIC AND IMMUNOMODULATING AGENTS | L01XX59 | IDH2 | Indications, How is this medicine expected to work |
| Encorafenib | ANTINEOPLASTIC AND IMMUNOMODULATING AGENTS | L01EC03 | BRAF, RAS | Indications, Warnings and precautions for use, Undesirable effects, Pharmacodynamic properties, Pharmacokinetic properties, Posology and method of administration |
| Enflurane | ANTINEOPLASTIC AND IMMUNOMODULATING AGENTS | N01AB04 | - | - |
| Entrectinib | ANTINEOPLASTIC AND IMMUNOMODULATING AGENTS | L01EX14 | NTRK, ROS1 | Indications, Posology and method of administration, Warnings and precautions for use, Undesirable effects, Pharmacodynamic properties, Pharmacokinetic properties, Pharmacodynamic properties |
| Enzalutamide | ANTINEOPLASTIC AND IMMUNOMODULATING AGENTS | L02BB | androgen receptor | Pharmacodynamic properties |
| Erdafitinib | ANTINEOPLASTIC AND IMMUNOMODULATING AGENTS | L01EX16 | X | X |
| Eribulin | ANTINEOPLASTIC AND IMMUNOMODULATING AGENTS | L01XX41 | HER2 | Pharmacodynamic properties |
| Erlotinib | ANTINEOPLASTIC AND IMMUNOMODULATING AGENTS | L01XE03 | EGFR, UGT1A1 | Indications, Posology and method of administration, Warnings and precautions for use, Interaction, Undesirable effects, Pharmacodynamic properties |
| Erythromycin and Sulfisoxazole | ANTIINFECTIVES FOR SYSTEMIC USE | J01FA01 |  |  |
| Escitalopram | NERVOUS SYSTEM | N06AB10 | X | X |
| Esomeprazole | ALIMENTARY TRACT AND METABOLISM\ | A02BC05 | CYP2C19 | Warnings and precautions for use, Interaction, Pharmacokinetic properties |
| Estradiol and Progesterone | GENITO URINARY SYSTEM AND SEX HORMONES | G03FB06 | X | X |
| Eteplirsen | MUSCULO-SKELETAL SYSTEM | M09AX06 | - | - |
| Ethinyl estradiol and Norelgestromin | GENITO URINARY SYSTEM AND SEX HORMONES | G03AA13 | Protein C, protein S, antithrombin-III, Factor V Leiden | Contraindications |
| Everolimus | ANTINEOPLASTIC AND IMMUNOMODULATING AGENTS | L01XE10 | HER2 | Indications, Pharmacodynamic properties |
| Evinacumab-dgnb | ALIMENTARY TRACT AND METABOLISM | - | homozygous familial hypercholesterolaemia (HoFH) | Therapeutic indications |
| Evolocumab | CARDIOVASCULAR SYSTEM | C10AX13 | PCSK9 | Indications, Interaction, Pharmacodynamic properties, Pharmacokinetic properties |
| Exemestane | ANTINEOPLASTIC AND IMMUNOMODULATING AGENTS | L02BG06 | X | X |
| Fampridine | NERVOUS SYSTEM | N07XX07 | X | X |
| Fam-Trastuzumab Deruxtecan-nxki | ANTINEOPLASTIC AND IMMUNOMODULATING AGENTS | L01XC03 | X | X |
| Fesoterodine | GENITO URINARY SYSTEM AND SEX HORMONES | G04BD11 | CYP2D6 | Warnings and precautions for use, Interaction, Pharmacokinetic properties, Preclinical safety data |
| Flibanserin | GENITO URINARY SYSTEM AND SEX HORMONES | G02CX02 | X | X |
| Fluorouracil | ANTINEOPLASTIC AND IMMUNOMODULATING AGENTS | L01BC02 | X | X |
| Fluoxetine | NERVOUS SYSTEM | N06AB03 | X | X |
| Flurbiprofen | MUSCULO-SKELETAL SYSTEM | M02AA19 | X | X |
| Flutamide | ANTINEOPLASTIC AND IMMUNOMODULATING AGENTS | L02BB01 | X | X |
| Fluvoxamine | NERVOUS SYSTEM | N06AB08 | CYP2D6 | Interaction, Pharmacokinetic properties |
| Formoterol | RESPIRATORY SYSTEM | R03CC | - | - |
| Fosphenytoin | NERVOUS SYSTEM | N03AB05 | - | - |
| Fulvestrant | ANTINEOPLASTIC AND IMMUNOMODULATING AGENTS | L02BA03 | HR, HER2 | Indications, Undesirable effects, Pharmacodynamic properties |
| Galantamine | NERVOUS SYSTEM | N06DA04 | X | X |
| Gefitinib | ANTINEOPLASTIC AND IMMUNOMODULATING AGENTS | L01XE02 | EGFR, CYP2D6 | Indications, Warnings and precautions for use, Pharmacodynamic properties, Pharmacokinetic properties, Preclinical safety data, Posology and method of administration, Interaction |
| Gemtuzumab Ozogamicin | ANTINEOPLASTIC AND IMMUNOMODULATING AGENTS | L01XC05 | CD33 | Indications, Pharmacodynamic properties, Pharmacokinetic properties |
| Gilteritinib | ANTINEOPLASTIC AND IMMUNOMODULATING AGENTS | L01EX13 | FLT3 | Indications, Posology and method of administration, Interaction, Pharmacodynamic properties, pharmacokinetic properties |
| Givosiran | ALIMENTARY TRACT AND METABOLISM\ | A16AX16 | X | X |
| Glimepiride | ALIMENTARY TRACT AND METABOLISM | A10BB12 | X | X |
| Glipizide | ALIMENTARY TRACT AND METABOLISM | A10BB07 | X | X |
| Glyburide | ALIMENTARY TRACT AND METABOLISM | A10BB01 | ATP-sensitive potassium channel, chromosome 6q24, G6PD | Indications, Pharmacodynamic properties, Warnings and precautions for use |
| Golodirsen | MUSCULO-SKELETAL SYSTEM | M09AX08 | - | - |
| Goserelin | ANTINEOPLASTIC AND IMMUNOMODULATING AGENTS | L02AE03 | X | X |
| Hydralazine | CARDIOVASCULAR SYSTEM | C02DB02 | - | - |
| Hydroxychloroquine | ANTIPARASITIC PRODUCTS, INSECTICIDES AND REPELLENTS | P01BA02 | X | X |
| Ibritumomab | VARIOUS | V10XX02 | CD20 / MS4A1 | Indications, Posology and method of administration, Pharmacodynamic properties |
| Ibrutinib | ANTINEOPLASTIC AND IMMUNOMODULATING AGENTS | L01XE27 | 17p, TP53, 11q | Pharmacodynamic properties |
| Iloperidone | NERVOUS SYSTEM | N05AX14 | - | - |
| Imatinib | ANTINEOPLASTIC AND IMMUNOMODULATING AGENTS | L01XE01 | KIT, BCR-ABL, PDGFR, FIP1L1-PDGFR | Indications, Warnings and precautions for use, Undesirable effects, Pharmacodynamic properties |
| Imipramine | NERVOUS SYSTEM | N06AA02 | - | - |
| Indacaterol | RESPIRATORY SYSTEM | R03AC18 | X | X |
| Inebilizumab-cdon | ANTINEOPLASTIC AND IMMUNOMODULATING AGENTS | L04AA47 | X | X |
| Infigratinib | ANTINEOPLASTIC AND IMMUNOMODULATING AGENTS | - | - | - |
| Inotersen | NERVOUS SYSTEM | N07XX15 | TTR | Indications, Undesirable effects, Pharmacodynamic properties, pharmacokinetic properties, Preclinical safety data |
| Inotuzumab Ozogamicin | ANTINEOPLASTIC AND IMMUNOMODULATING AGENTS | L01XC26 | CD22, Philadelphia chromosome | Indications, Posology and method of administration, Pharmacodynamic properties |
| Ipilimumab | ANTINEOPLASTIC AND IMMUNOMODULATING AGENTS | L01XC11 | PD-L1, ALK, EGFR | Indications, Warnings and precautions for use, Pharmacodynamic properties |
| Irinotecan | ANTINEOPLASTIC AND IMMUNOMODULATING AGENTS | L01XX19 | UGT1A1 | Posology and method of administration, Warnings and precautions for use, Interaction, Undesirable effects, pharmacodynamic properties, pharmacokinetic properties |
| Isatuximab- irfc | ANTINEOPLASTIC AND IMMUNOMODULATING AGENTS | L01XC38 | CD38 | Warnings and precautions for use, Interaction, Pharmacodynamic properties, Pharmacokinetic properties |
| Isoflurane | NERVOUS SYSTEM | N01AB06 | X | X |
| Isoniazid | ANTIINFECTIVES FOR SYSTEMIC USE | J04AC01 | X | X |
| Isoniazid, Pyrazinamide, and Rifampin | ANTIINFECTIVES FOR SYSTEMIC USE | J04AM05 | - | - |
| Isosorbide Dinitrate | CARDIOVASCULAR SYSTEM | C01DA08 | - | - |
| Isosorbide Mononitrate | CARDIOVASCULAR SYSTEM | C01DA14 | - | - |
| Ivabradine | RESPIRATORY SYSTEM | R07AX02 | X | X |
| Ivacaftor and Lumacaftor | RESPIRATORY SYSTEM | R07AX30 | CFTR | Indications, Posology and method of administration, Warnings and precautions for use, Undesirable effects, Pharmacodynamic properties |
| Ivacaftor and Tezacaftor | RESPIRATORY SYSTEM | R07AX31 | CFTR | Indications, Warnings and precautions for use, Pharmacodynamic properties |
| Ivosidenib | ANTINEOPLASTIC AND IMMUNOMODULATING AGENTS | L01XX62 | X | X |
| Ixabepilone | ANTINEOPLASTIC AND IMMUNOMODULATING AGENTS | L01DC04 | - | - |
| Lacosamide | NERVOUS SYSTEM | N03AX18 | CYP2C19 | Interaction, Pharmacokinetic properties |
| Lansoprazole | ALIMENTARY TRACT AND METABOLISM\ | A02BC03 | CYP2C19 | Interaction, Pharmacokinetic properties |
| Lapatinib | ANTINEOPLASTIC AND IMMUNOMODULATING AGENTS | L01XE07 | HER2, oestrogen receptor [ER], progesterone receptor [PgR], HLA-DQA1, HLA-DRB1 | Indications, Posology and method of administration, Overdose, Pharmacodynamic properties, warnings and precautions for use, Undesirable effects |
| Larotrectinib | ANTINEOPLASTIC AND IMMUNOMODULATING AGENTS | L01EX12 | NTRK | Indications, Posology and method of administration, Warnings and precautions for use, Pharmacodynamic properties |
| Ledipasvir and Sofosbuvir | ANTIINFECTIVES FOR SYSTEMIC USE | J05AP51 | IL28B | Pharmacodynamic properties |
| Lenalidomide | ANTINEOPLASTIC AND IMMUNOMODULATING AGENTS | L04AX04 | 5q | Indications, Warnings and precautions for use, Undesirable effects, Pharmacodynamic properties |
| Lenvatinib | ANTINEOPLASTIC AND IMMUNOMODULATING AGENTS | L01XE29 | X | X |
| Lesinurad | MUSCULO-SKELETAL SYSTEM | M04AB05 | - | - |
| Letrozole | ANTINEOPLASTIC AND IMMUNOMODULATING AGENTS | L02BG04 | ER, PgR | Pharmacodynamic properties |
| Lidocaine and Prilocaine | NERVOUS SYSTEM | N01BB | glucose-6-phosphate dehydrogenase | Special warnings and precautions for use |
| Lidocaine and Tetracaine | NERVOUS SYSTEM | N01BB52 | X | X |
| Lofexidine | NERVOUS SYSTEM | N07BC04 | - | - |
| Lomitapide | CARDIOVASCULAR SYSTEM | C10AX12 | X | X |
| Lonafarnib | ALIMENTARY TRACT AND METABOLISM | - | - | - |
| Lorlatinib | ANTINEOPLASTIC AND IMMUNOMODULATING AGENTS | L01ED05 | ALK | Indications, Pharmacodynamic properties |
| Lumasiran | ALIMENTARY TRACT AND METABOLISM | A16AX18 | AGXT | Pharmacodynamic properties |
| Luspatercept–aamt | BLOOD AND BLOOD FORMING ORGANS | B03XA06 | Smad2/3 signalling | Pharmacodynamic properties |
| Lusutrombopag | BLOOD AND BLOOD FORMING ORGANS | B02BX07 | X | X |
| Lutetium Dotatate Lu-177 | VARIOUS | V10XX04 | X | X |
| Mafenide | DERMATOLOGICALS | D06BA03 | - | - |
| Maraviroc | ANTIINFECTIVES FOR SYSTEMIC USE | J05AX09 | CCR5, CXCR4 | Indications, Posology and method of administration, Warnings and precautions for use, Fertility/pregnancy and lactation, Undesirable effects, Pharmacodynamic properties, Pharmacokinetic properties, Preclinical safety data |
| Margetuximab-cmkb | ANTINEOPLASTIC AND IMMUNOMODULATING AGENTS | - | - | - |
| Meclizine | RESPIRATORY SYSTEM | R06AE05 | - | - |
| Meloxicam | MUSCULO-SKELETAL SYSTEM | M01AC06 | X | X |
| Mepivacaine | NERVOUS SYSTEM | N01BB03 | X | X |
| Mercaptopurine | ANTINEOPLASTIC AND IMMUNOMODULATING AGENTS | L01BB02 | TPMT(1) NUDT15(2) | Posology and method of administration, Warnings and precautions for use, Interaction, Pharmacokinetic properties |
| Methylene Blue | VARIOUS | V03AB | G6PD | Contraindications |
| Metoclopramide | ALIMENTARY TRACT AND METABOLISM\ | A03FA01 | X | X |
| Metoprolol | CARDIOVASCULAR SYSTEM | C07AB02 | - | - |
| Metreleptin | ALIMENTARY TRACT AND METABOLISM | A16AA07 | X | X |
| Midostaurin | ANTINEOPLASTIC AND IMMUNOMODULATING AGENTS | L01XE39 | FLT3, KIT | Indications, Posology and method of administration, Undesirable effects, Pharmacodynamic properties |
| Migalastat | ALIMENTARY TRACT AND METABOLISM\ | A16AX14 | GLA | Pharmacodynamic properties |
| Mirabegron | GENITO URINARY SYSTEM AND SEX HORMONES | G04BD12 | CYP2D6 | Interaction, Pharmacokinetic properties |
| Mivacurium | MUSCULO-SKELETAL SYSTEM | M03AC10 | - | - |
| Modafinil | NERVOUS SYSTEM | N06BA07 | X | X |
| Moviprep | Various | - | - | - |
| Mycophenolic Acid | ANTINEOPLASTIC AND IMMUNOMODULATING AGENTS | L04AA06 | HGPRT | Special warnings and precautions for use |
| Nalidixic Acid | ANTIINFECTIVES FOR SYSTEMIC USE | J01MB02 | X | X |
| Nebivolol | CARDIOVASCULAR SYSTEM | C07AB12 | X | X |
| Nefazodone | NERVOUS SYSTEM | N06AX06 | - | - |
| Neratinib | ANTINEOPLASTIC AND IMMUNOMODULATING AGENTS | L01EH02 | HER2 | Indications, Warnings and precautions for use, Pharmacodynamic properties, Pharmacokinetic properties |
| Nilotinib | ANTINEOPLASTIC AND IMMUNOMODULATING AGENTS | L01XE08 | BCR-ABL, Philadelphia chromosome | Indications, Posology and method of administration, Warnings and precautions for use, Undesirable effects, Pharmacodynamic properties |
| Niraparib | ANTINEOPLASTIC AND IMMUNOMODULATING AGENTS | L01XX54 | BRCA | Pharmacodynamic properties |
| Nitrofurantoin | ANTIINFECTIVES FOR SYSTEMIC USE | J01XE01 | - | - |
| Nivolumab | ANTINEOPLASTIC AND IMMUNOMODULATING AGENTS | L01XC17 | BRAF, PD-L1  EGFR, ALK | Indications, Warnings and precautions for use, Undesiable effects, Pharmacodynamic properties, Preclinical safety data |
| Norfloxacin | ANTIINFECTIVES FOR SYSTEMIC USE | J01MA06 | X | X |
| Nortriptyline | NERVOUS SYSTEM | N06AA10 | X | X |
| Nusinersen | MUSCULO-SKELETAL SYSTEM | M09AX07 | SMN2 | Pharmacodynamic properties |
| Obinutuzumab | ANTINEOPLASTIC AND IMMUNOMODULATING AGENTS | L01XC15 | CD20 | Warnings and precautions for use, Pharmacodynamic properties, Pharmacokinetic properties |
| Ofatumumab | Antineoplastic and immunomodulating agents | L01XC10 | X | X |
| Olaparib | ANTINEOPLASTIC AND IMMUNOMODULATING AGENTS | L01XX46 | BRCA, HER2, genomic instability | Indications, Posology and method of administration, Warnings and precautions for use, Pharmacodynamic properties |
| Olaratumab | ANTINEOPLASTIC AND IMMUNOMODULATING AGENTS | L01XC27 | - | - |
| Oliceridine | NERVOUS SYSTEM | - | - | - |
| Omacetaxine | ANTINEOPLASTIC AND IMMUNOMODULATING AGENTS | L01XX40 | - | - |
| Ombitasvir, Paritaprevir, and Ritonavir | ANTIINFECTIVES FOR SYSTEMIC USE | J05AP53 | X | X |
| Omeprazole | ALIMENTARY TRACT AND METABOLISM\ | A02BC01 | CYP2C19 | Warnings and precautions for use, Interaction, Pharmacokinetic properties |
| Ondansetron | ALIMENTARY TRACT AND METABOLISM\ | A04AA01 | X | X |
| Osimertinib | ANTINEOPLASTIC AND IMMUNOMODULATING AGENTS | L01XE35 | EGFR | Indications, Posology and method of administration, Warnings and precautions for use, Undesirable effects, Overdose, Pharmacodynamic properties, Pharmacokinetic properties |
| Ospemifene | GENITO URINARY SYSTEM AND SEX HORMONES | G03XC05 | CYP2C9 | Interaction, Pharmacokinetic properties |
| Oxcarbazepine | NERVOUS SYSTEM | N03AF02 | X | X |
| Oxymetazoline and Tetracaine | RESPIRATORY SYSTEM | R01AA05 | X | X |
| Palbociclib | ANTINEOPLASTIC AND IMMUNOMODULATING AGENTS | L01XE33 | HER2 | Indications, Undesirable effects, Pharmacodynamic properties |
| Paliperidone | NERVOUS SYSTEM | N05AX13 | CYP2D6 | Interaction, Pharmacokinetic properties |
| Palonosetron | ALIMENTARY TRACT AND METABOLISM\ | A04AA05 | CYP2D6 | Interaction, Pharmacokinetic properties |
| Panitumumab | ANTINEOPLASTIC AND IMMUNOMODULATING AGENTS | L01XC08 | RAS, EGFR | Indications, Posology and method of administration, Contraindications,  Warnings and precautions for use, Interaction, Pharmacodynamic properties, Fertility/pregnancy and lactation, Undesirable effects, Pharmacokinetic properties, Preclinical safety data |
| Pantoprazole | ALIMENTARY TRACT AND METABOLISM\ | A02BC02 | X | X |
| Parathyroid Hormone | SYSTEMIC HORMONAL PREPARATIONS, EXCL. SEX HORMONES AND INSULINS | H05AA03 | X | X |
| Paroxetine | NERVOUS SYSTEM | N06AB05 | X | X |
| Patisiran | NERVOUS SYSTEM | N07XX12 | Transthyretin (TTR) | Indications, Warnings and precautions for use, interaction, Undesirable effects, Pharmacodynamic properties, pharmacokinetic properties, Preclinical safety data |
| Pazopanib | ANTINEOPLASTIC AND IMMUNOMODULATING AGENTS | L01XE11 | HLA-B | Warnings and precautions for use, Pharmacodynamic properties |
| Peginterferon Alfa-2b | ANTINEOPLASTIC AND IMMUNOMODULATING AGENTS | L03AB10 | X | X |
| Pegloticase | MUSCULO-SKELETAL SYSTEM | M04AX02 | G6PD | Contraindications |
| Pembrolizumab | ANTINEOPLASTIC AND IMMUNOMODULATING AGENTS | L01XC18 | BRAF, PD-L1, EGFR, ALK, CD274 | Indications, Posology and method of administration, Warnings and precautions for use, Undesirable effects, Pharmacodynamic properties, Preclinical safety data |
| Pemigatinib | ANTINEOPLASTIC AND IMMUNOMODULATING AGENTS | L01EX20 | FGFR2 | Therapeutic indications, Posology and method of administration, Pharmacodynamic properties, Preclinical safety data |
| Perphenazine | NERVOUS SYSTEM | N05AB03 | X | X |
| Pertuzumab | ANTINEOPLASTIC AND IMMUNOMODULATING AGENTS | L01XC13 | HER2 | Indications, Posology and method of administration, Warnings and precautions for use, Pharmacodynamic properties |
| Phenytoin | NERVOUS SYSTEM | N03AB02 | - | - |
| Pimozide | NERVOUS SYSTEM | N05AG02 | - | - |
| Piroxicam | MUSCULO-SKELETAL SYSTEM | M01AC01 | X | X |
| Pitolisant | NERVOUS SYSTEM | N07XX11 | CYP2D6 | Posology and method of administration, Interaction, Pharmacokinetic properties |
| Ponatinib | ANTINEOPLASTIC AND IMMUNOMODULATING AGENTS | L01XE24 | BCR-ABL | Indications, Posology and method of administration, Warnings and precautions for use, Undesirable effects, Pharmacodynamic properties |
| Pralsetinib | ANTINEOPLASTIC AND IMMUNOMODULATING AGENTS | L01EX23 | - | - |
| Prasugrel | BLOOD AND BLOOD FORMING ORGANS | B01AC22 | CYP3A, CYP2B6, CYP2C9, CYP2C19 | Interaction, Pharmacokinetic properties, interaction |
| Primaquine | ANTIPARASITIC PRODUCTS, INSECTICIDES AND REPELLENTS | P01BA03 | - | - |
| Probenecid | MUSCULO-SKELETAL SYSTEM | M04AB01 | - | - |
| Procainamide | CARDIOVASCULAR SYSTEM | C01BA02 | - | - |
| Propafenone | CARDIOVASCULAR SYSTEM | C01BC03 | X | X |
| Propranolol | CARDIOVASCULAR SYSTEM | C07AA05 | CYP2D6 | Pharmacokinetic properties |
| Protriptyline | NERVOUS SYSTEM | N06AA11 | - | - |
| Quinidine | CARDIOVASCULAR SYSTEM | C01BA01 | - | - |
| Quinine Sulfate | ANTIPARASITIC PRODUCTS, INSECTICIDES AND REPELLENTS | P01BC01 | X | X |
| Rabeprazole | ALIMENTARY TRACT AND METABOLISM\ | A02BC04 | X | X |
| Raloxifene | GENITO URINARY SYSTEM AND SEX HORMONES | G03XC01 | Oestrogen Receptor | Pharmacodynamic properties |
| Raltegravir | ANTIINFECTIVES FOR SYSTEMIC USE | J05AX08 | UGT1A1 | Interaction, Pharmacokinetic properties |
| Ramucirumab | ANTINEOPLASTIC AND IMMUNOMODULATING AGENTS | L01XC21 | EGFR, KRAS | Indications, Posology and method of administration, Warnings and precautions for use, Pharmacodynamic properties |
| Ranolazine | CARDIOVASCULAR SYSTEM | C01EB18 | CYP2D6 | Warnings and precautions for use, Interaction, pharmacokinetic properties |
| Rasburicase | VARIOUS | V03AF07 | G6PD | Contraindications, Undesirable effects, Pharmacodynamic properties |
| Regorafenib | ANTINEOPLASTIC AND IMMUNOMODULATING AGENTS | L01XE21 | KRAS | Pharmacodynamic properties |
| Ribociclib | ANTINEOPLASTIC AND IMMUNOMODULATING AGENTS | L01XE42 | HR, HER2 | Indications, Undesirable effects, Pharmacodynamic properties |
| Rimegepant | NERVOUS SYSTEM | - | - | - |
| Risdiplam | MUSCULO-SKELETAL SYSTEM | M09AX10 | SMA Type 1, Type 2 or Type 3, SMN2 | Therapeutic indications, Undesirable effects, Pharmacodynamic properties |
| Risperidone | NERVOUS SYSTEM | N05AX08 | CYP2D6 | Interaction, Pharmacokinetic properties |
| Rituximab | ANTINEOPLASTIC AND IMMUNOMODULATING AGENTS | L01XC02 | CD20, MS4A1 | Indications, Posology and method of administration, Undesirable effects, Pharmacodynamic properties, Preclinical safety data |
| Rivaroxaban | BLOOD AND BLOOD FORMING ORGANS | B01AF01 | Factor Xa | Warnings and precautions for use, Interaction, Overdose, Pharmacodynamic properties, Pharmacokinetic properties |
| Ropivacaine | NERVOUS SYSTEM | N01BB09 | X | X |
| Rosuvastatin | CARDIOVASCULAR SYSTEM | C10AA07 | X | X |
| Rucaparib | ANTINEOPLASTIC AND IMMUNOMODULATING AGENTS | L01XK03 | CYP2D6, CYP1A2, BRCA | Interaction, Pharmacokinetic properties, Indications, Posology and method of administration, Pharmacodynamic properties |
| Ruxolitinib | ANTINEOPLASTIC AND IMMUNOMODULATING AGENTS | L01XE18 | X | X |
| Sacituzumab Govitecan-hziy | ANTINEOPLASTIC AND IMMUNOMODULATING AGENTS | L01 | X | X |
| Satralizumab-mwge | ANTINEOPLASTIC AND IMMUNOMODULATING AGENTS | L04AC19 | anti-aquaporin-4, IgG (AQP4-IgG) | Therapeutic indications |
| Selpercatinib | ANTINEOPLASTIC AND IMMUNOMODULATING AGENTS | L01 | RET | Therapeutic indications |
| Setmelanotide | ALIMENTARY TRACT AND METABOLISM | - | POMC, PCSK1, LEPR | Therapeutic indications, Posology and method of administration |
| Sevoflurane | NERVOUS SYSTEM | N01AB08 | X | X |
| Sildenafil | GENITO URINARY SYSTEM AND SEX HORMONES | G04BE03 | Retinal phosphodiesterases | Warnings and precautions for use |
| Simeprevir | ANTIINFECTIVES FOR SYSTEMIC USE | J05AP05 | X | X |
| Siponimod | ANTINEOPLASTIC AND IMMUNOMODULATING AGENTS | L04AA42 | CYP2C9 | Posology and method of administration, Contraindications, warnings and precautions for use, Interaction, Pharmacodynamic properties, Pharmacokinetic properties |
| Sodium Nitrite | VARIOUS | V03AB08 | X | X |
| Sodium Oxybate | NERVOUS SYSTEM | N01AX11 | Succinic semialdehyde dehydrogenase deficiency | Contraindications |
| Sodium Phenylbutyrate | ALIMENTARY TRACT AND METABOLISM\ | A16AX03 | CPS1 (carbamylphosphate synthetase), OTC (ornithine transcarbamylase) or ASS1 (argininosuccinate synthetase) | Indications, Pharmacodynamic properties |
| Sofosbuvir | ANTIINFECTIVES FOR SYSTEMIC USE | J05AP08 | IL28B | Warnings and precautions for use, Pharmacodynamic properties |
| Sofosbuvir and Velpatasvir | ANTIINFECTIVES FOR SYSTEMIC USE | J05AP55 | IL28B | Pharmacodynamic properties |
| Sofosbuvir, Velpatasvir, and Voxilaprevir | ANTIINFECTIVES FOR SYSTEMIC USE | J05AP56 | IL28B | Pharmacodynamic properties |
| Sotorasib | ANTINEOPLASTIC AND IMMUNOMODULATING AGENTS | L01XX73 | - | - |
| Succimer | VARIOUS | V09CA02 | - | - |
| Succinylcholine | MUSCULO-SKELETAL SYSTEM | M03AB01 | - | - |
| Sulfadiazine | DERMATOLOGICALS | D06BA01 | - | - |
| Sulfamethoxazole and Trimethoprim | ANTIINFECTIVES FOR SYSTEMIC USE | J01EE01 | X | X |
| Sulfasalazine | ALIMENTARY TRACT AND METABOLISM\ | A07EC01 | - | - |
| Synthetic Conjugated Estrogens, A | GENITO URINARY SYSTEM AND SEX HORMONES | - | - | - |
| Tafamidis | NERVOUS SYSTEM | N07XX08 | Transthyretin (TTR) | Indications, Posology and method of administration, Warnings and precautions for use, Interaction, Undesirable effects, Overdose, Pharmacodynamic properties, Pharmacokinetic properties |
| Tafenoquine | ANTIINFECTIVES FOR SYSTEMIC USE | P01BA07 |  |  |
| Talazoparib | ANTINEOPLASTIC AND IMMUNOMODULATING AGENTS | L01XK04 | BRCA, HER2 | Indications, Posology and method of administration, Undesirable effects, Pharmacodynamic properties |
| Tamoxifen | ANTINEOPLASTIC AND IMMUNOMODULATING AGENTS | L02BA01 | X | X |
| Tamsulosin | GENITO URINARY SYSTEM AND SEX HORMONES | G04CA02 | X | X |
| Telaprevir | ANTIINFECTIVES FOR SYSTEMIC USE | J05AP02 | - | - |
| Tepotinib | ANTINEOPLASTIC AND IMMUNOMODULATING AGENTS | L01EX21 | - | - |
| Tetrabenazine | NERVOUS SYSTEM | N07XX06 | X | X |
| Thioguanine | ANTINEOPLASTIC AND IMMUNOMODULATING AGENTS | L01BB03 | X | X |
| Thioridazine | NERVOUS SYSTEM | N05AC02 | - | - |
| Ticagrelor | BLOOD AND BLOOD FORMING ORGANS | B01AC24 | CYP2C19 | Pharmacodynamic properties |
| Tipiracil and Trifluridine | ANTINEOPLASTIC AND IMMUNOMODULATING AGENTS | L01BC59 | HER2, KRAS | Pharmacodynamic properties |
| Tolazamide | ALIMENTARY TRACT AND METABOLISM | A10BB05 | - | - |
| Tolbutamide | ALIMENTARY TRACT AND METABOLISM | A10BB03 | - | - |
| Tolterodine | GENITO URINARY SYSTEM AND SEX HORMONES | G04BD07 | X | X |
| Toremifene | ANTINEOPLASTIC AND IMMUNOMODULATING AGENTS | L02BA02 | estrogen receptor | Indications, Pharmacodynamic properties |
| Tramadol | NERVOUS SYSTEM | N02AX02 | CYP2D6 | Warnings and precautions for use |
| Trametinib | ANTINEOPLASTIC AND IMMUNOMODULATING AGENTS | L01XE25 | BRAF V600 | Indications, Posology and method of administration, Warnings and precautions for use, Undesirable effects, Pharmacodynamic properties |
| Trastuzumab | ANTINEOPLASTIC AND IMMUNOMODULATING AGENTS | L01XC03 | HER2 | Indications, Posology and method of administration, Warnings and precautions for use, Interaction, Pharmacokinetic properties, Pharmacodynamic properties |
| Tretinoin | ANTINEOPLASTIC AND IMMUNOMODULATING AGENTS | L01XX14 | X | X |
| Triheptanoin | ALIMENTARY TRACT AND METABOLISM\ | A16AX17 | X | X |
| Trimipramine | NERVOUS SYSTEM | N06AA06 | - | - |
| Tucatinib | ANTINEOPLASTIC AND IMMUNOMODULATING AGENTS | L01EH03 | HER2 | Therapeutic indication |
| Umeclidinium | RESPIRATORY SYSTEM | R03AL03 | CYP2D6 | Interaction, Pharmacokinetic properties |
| Upadacitinib | ANTINEOPLASTIC AND IMMUNOMODULATING AGENTS | L04AA44 | X | X |
| Ustekinumab | ANTINEOPLASTIC AND IMMUNOMODULATING AGENTS | L04AC05 | IL-12, IL-23 | Pharmacodynamic properties, Pharmacokinetic properties, Preclinical safety data |
| Valbenazine | NERVOUS SYSTEM | N07XX13 | - | - |
| Valproic Acid | NERVOUS SYSTEM | N03AG01 | X | X |
| Vandetanib | ANTINEOPLASTIC AND IMMUNOMODULATING AGENTS | L01XE12 | RET | Indications, Warnings and precautions for use, Pharmacodynamic properties |
| Velaglucerase alfa | ALIMENTARY TRACT AND METABOLISM\ | A16AB10 | Glucocerebrosidase, GBA gene | Fertility/pregnancy and lactation, Pharmacodynamic properties |
| Vemurafenib | ANTINEOPLASTIC AND IMMUNOMODULATING AGENTS | L01XE15 | BRAF, RAS | Indications, Posology and method of administration, Warnings and precautions for use, Undesirable effects, Pharmacodynamic properties, Pharmacokinetic properties |
| Venetoclax | ANTINEOPLASTIC AND IMMUNOMODULATING AGENTS | L01XX52 | 17p, TP53, 11q | Indications, Undesirable effects, Pharmacodynamic properties |
| Venlafaxine | NERVOUS SYSTEM | N06AX16 | CYP2D6 | Interaction, Pharmacokinetic properties |
| Viloxazine | NERVOUS SYSTEM | N06AX09 | - | - |
| Viltolarsen | NERVOUS SYSTEM | - | - | - |
| Vincristine | ANTINEOPLASTIC AND IMMUNOMODULATING AGENTS | L01CA02 | X | X |
| Vitamin C | Alimentary tract and metabolism | A11GA01 | - | - |
| Voriconazole | NERVOUS SYSTEM | J02AC03 | CYP2C19 | Warnings and precautions for use, Interaction, Pharmacokinetic properties |
| Vortioxetine | NERVOUS SYSTEM | N06AX26 | CYP2D6 | Posology and method of administration, Interaction, Pharmacokinetic properties |
| Voxelotor | BLOOD AND BLOOD FORMING ORGANS | - | X | X |
| Warfarin | BLOOD AND BLOOD FORMING ORGANS | B01AA03 | - | - |

**S5 Table.** Contents of drug labeling in Japan

| **Drug** | **Drug (Japanese)** | **Therapeutic Area** | **ATC code** | **Biomarker** | **Labelling Section** |
| --- | --- | --- | --- | --- | --- |
| Abacavir | アバカビル | ANTIINFECTIVES FOR SYSTEMIC USE | J05AF06 | HLA-B*5701 | Other notes |
| Abemaciclib | アベマシクリブ | ANTINEOPLASTIC AND IMMUNOMODULATING AGENTS | L01EF03 | Hormone receptor, HER-2 | Efficacy or Effect |
| Ado-Trastuzumab Emtansine | トラスツズマブエムタンシン | ANTINEOPLASTIC AND IMMUNOMODULATING AGENTS | L01XC14 | HER-2 | Efficacy or effect, Precautions related to efficacy or effect, Pharmacokinetics, Clinical results, Pharmacology |
| Aducanumab-avwa | - | NERVOUS SYSTEM | - | - | - |
| Afatinib | アファチニブ | ANTINEOPLASTIC AND IMMUNOMODULATING AGENTS | L01XE13 | EGFR | Efficacy or effect, Precautions related to Efficacy or effect, Clinical results |
| Alectinib | アレクチニブ | ANTINEOPLASTIC AND IMMUNOMODULATING AGENTS | L01XE36 | ALK | Efficacy or effect, Precautions related to Efficacy or effect, Usage and dosage, Precautions regarding patients with specific backgrounds |
| lucosidase Alfa | アルグルコシダーゼアルファ | ALIMENTARY TRACT AND METABOLISM\ | A16AB07 | X |  |
| Alirocumab | アリロクマブ | CARDIOVASCULAR SYSTEM | C10AX14 | X |  |
| Aliskiren | アリスキレン | CARDIOVASCULAR SYSTEM | C09XA02 | X |  |
| Allopurinol | アロプリノール | MUSCULO-SKELETAL SYSTEM | M04AA01 | HLA-B * 5801 | Other notes |
| Alpelisib | - | ANTINEOPLASTIC AND IMMUNOMODULATING AGENTS | L01EM03 | - | - |
| Amifampridine | - | NERVOUS SYSTEM | N07XX05 | - | - |
| Amitriptyline | アミトリプチリン | NERVOUS SYSTEM | N06AA09 | X |  |
| Amivantamab-vmjw | - | ANTINEOPLASTIC AND IMMUNOMODULATING AGENTS | - | - | - |
| Amoxapine | アモキサピン | NERVOUS SYSTEM | N06AA17 | X |  |
| Amphetamine | - | NERVOUS SYSTEM | N06BA01 | - | - |
| Anakinra | - | ANTINEOPLASTIC AND IMMUNOMODULATING AGENTS | L04AC03 | - | - |
| Anastrozole | アナストロゾール | ANTINEOPLASTIC AND IMMUNOMODULATING AGENTS | L02BG03 | X |  |
| Arformoterol | - | RESPIRATORY SYSTEM | R03 |  |  |
| Aripiprazole | アリピプラゾール | NERVOUS SYSTEM | N05AX12 | X |  |
| Arsenic Trioxide | 三酸化二ヒ素 | ANTINEOPLASTIC AND IMMUNOMODULATING AGENTS | L01XX27 | PML-RAR-α | Precautions related to efficacy or effect |
| Articaine and Epinephrine | - | NERVOUS SYSTEM | N01BB58 | - | - |
| Ascorbic Acid, PEG-3350, Potassium Chloride, Sodium Ascorbate, Sodium Chloride, and Sodium Sulfate | - | ALIMENTARY TRACT AND METABOLISM\ | A06AD65 | - | - |
| Atazanavir | アタザナビル | ANTIINFECTIVES FOR SYSTEMIC USE | J05AE08 | CYP2C19 | Interaction |
| Atezolizumab | アテゾリズマブ | ANTINEOPLASTIC AND IMMUNOMODULATING AGENTS | L01XC32 | PD-L1, HER2, EGFR, ALK | Efficacy or effect, Precautions related to efficacy or effect, Dosage and administration, Precautions related to usage and dosage, Clinical results |
| Atomoxetine | アトモキセチン | NERVOUS SYSTEM | N06BA09 | CYP2D6 | Precautions related to usage and dosage, Precautions regarding patients with specific backgrounds, Other notes, Pharmacokinetics |
| Atorvastatin | アトルバスタチン | CARDIOVASCULAR SYSTEM | C10AA05 | LDLR (familial hypercholesterolemia) | Precautions related to efficacy or effect, Clinical results |
| Avapritinib | - | ANTINEOPLASTIC AND IMMUNOMODULATING AGENTS | L01EX18 | - | - |
| Avatrombopag | - | BLOOD AND BLOOD FORMING ORGANS | B02BX08 | - | - |
| Avelumab | アベルマブ | ANTINEOPLASTIC AND IMMUNOMODULATING AGENTS | L01XC31 | PD-L1 | Clinical result |
| Axitinib | アキシチニブ | ANTINEOPLASTIC AND IMMUNOMODULATING AGENTS | L01XE17 | X |  |
| Azathioprine | アザチオプリン | ANTINEOPLASTIC AND IMMUNOMODULATING AGENTS | L04AX01 | NUDT15, TPMT | Other notes, Pharmacokinetics |
| Belimumab | ベリムマブ | ANTINEOPLASTIC AND IMMUNOMODULATING AGENTS | L04AA26 | X |  |
| Belinostat | - | ANTINEOPLASTIC AND IMMUNOMODULATING AGENTS | L01XH04 | - | - |
| Binimetinib | ビニメチニブ | ANTINEOPLASTIC AND IMMUNOMODULATING AGENTS | L01EE03 | BRAF V600E | Efficacy or effect, Precautions related to efficacy or effect, Usage and dosage, Precautions related to usage and dosage, Pharmacokinetics, Clinical results |
| Blinatumomab | ブリナツモマブ | ANTINEOPLASTIC AND IMMUNOMODULATING AGENTS | L01XC19 | X |  |
| Boceprevir | - | ANTIINFECTIVES FOR SYSTEMIC USE | J05AP03 | - | - |
| Bosutinib | ボスチニブ | ANTINEOPLASTIC AND IMMUNOMODULATING AGENTS | - | BCR-ABL | Precautions related to efficacy or effect, Clinical results |
| Brentuximab Vedotin | ブレンツキシマブベドチン | ANTINEOPLASTIC AND IMMUNOMODULATING AGENTS | L01XC12 | CD30 | Efficacy or effect, Precautions related to efficacy or effect, Usage and dosage, Precautions related to usage and dosage, Important basic notes, Pharmacokinetics, Clinical results |
| Brexpiprazole | ブレクスピプラゾール | NERVOUS SYSTEM | N05AX16 | CYP2D6 | Pharmacokinetics |
| Brigatinib | ブリグチニブ | ANTINEOPLASTIC AND IMMUNOMODULATING AGENTS | L01EA04 | ALK | Efficacy or effect, Precautions related to efficacy or effect, Pharmacokinetics, Clinical results |
| Brivaracetam | - | NERVOUS SYSTEM | N03AX23 | - | - |
| Bupivacaine | ブピバカイン | NERVOUS SYSTEM | N01BB01 | X | X |
| Bupropion | - | NERVOUS SYSTEM | N06AX12 | - | - |
| Busulfan | ブスルファン | ANTINEOPLASTIC AND IMMUNOMODULATING AGENTS | L01AB01 | X |  |
| Cabotegravir and Rilpivirine | - | ANTIINFECTIVES FOR SYSTEMIC USE | J05AJ04 | - | - |
| Cabozantinib | カボザンチニブ | ANTINEOPLASTIC AND IMMUNOMODULATING AGENTS | L01XE26 | X |  |
| Capecitabine | カペシタビン | ANTINEOPLASTIC AND IMMUNOMODULATING AGENTS | L01BC06 | DPD | Other notes |
| Capmatinib | カプマチニブ | ANTINEOPLASTIC AND IMMUNOMODULATING AGENTS | L01EX17 | MET gene exon 14 | Efficacy or effect, Precautions related to efficacy or effect, Interaction, Pharmacokinetics, Pharmacology |
| Carbamazepine | カルバマゼピン | NERVOUS SYSTEM | N03AF01 | HLA-A*3101, HLA-B*1502 | Other notes |
| Carglumic Acid | カルグルミン酸 | ALIMENTARY TRACT AND METABOLISM\ | A16AA05 | NAGS | Efficacy or effect, Side effects, Clinical results |
| Cariprazine | - | NERVOUS SYSTEM | N05AX15 | - | - |
| Carisoprodol | - | MUSCULO-SKELETAL SYSTEM | M03BA02 | - | - |
| Carvedilol | カルベジロール | CARDIOVASCULAR SYSTEM | C07AG02 | X |  |
| Casimersen | - | NERVOUS SYSTEM | - | - | - |
| Ceftriaxone | セフトリアキソン | ANTIINFECTIVES FOR SYSTEMIC USE | J01DD04 | X |  |
| Celecoxib | セレコキシブ | ANTINEOPLASTIC AND IMMUNOMODULATING AGENTS | M01AH01 | CYP2C9 | Pharmacokinetics |
| Cemiplimab-rwlc | - | ANTINEOPLASTIC AND IMMUNOMODULATING AGENTS | L01XC33 | - | - |
| Ceritinib | セリチニブ | ANTINEOPLASTIC AND IMMUNOMODULATING AGENTS | L01XE28 | ALK | Efficacy or effect, Precautions related to efficacy or effect, Side effects, Pharmacokinetics, Clinical results |
| Cerliponase Alfa | セルリポナーゼ アルファ | ALIMENTARY TRACT AND METABOLISM\ | A16AB17 | Ceroid lipofuscinosis type 2 | Efficacy or effect |
| Cetuximab | セツキシマブ | ANTINEOPLASTIC AND IMMUNOMODULATING AGENTS | L01XC06 | EGFR, RAS | Efficacy or effect, Precautions related to usage and dosage, Clinical results |
| Cevimeline | セビメリン | NERVOUS SYSTEM | N07AX03 | X |  |
| Chloroprocaine | - | NERVOUS SYSTEM | N01BA04 | - | - |
| Chloroquine | - | ANTIPARASITIC PRODUCTS, INSECTICIDES AND REPELLENTS | P01BA02 | - | - |
| Chlorpropamide | クロルプロパミド | ALIMENTARY TRACT AND METABOLISM | A10BB02 | X |  |
| Cholic acid | - | ALIMENTARY TRACT AND METABOLISM | A05AA03 | - | - |
| Cisplatin | シスプラチン | ANTINEOPLASTIC AND IMMUNOMODULATING AGENTS | L01XA01 | X |  |
| Citalopram | - | NERVOUS SYSTEM | N06AB10 | - | - |
| Clobazam | クロバザム | NERVOUS SYSTEM | N05BA09 | X |  |
| Clomipramine | クロミプラミン | NERVOUS SYSTEM | N06AA04 | X |  |
| Clopidogrel | クロピドグレル | BLOOD AND BLOOD FORMING ORGANS | B01AC04 | CYP2C19 / HLA-DR4 | Other notes, Pharmacokinetics |
| Clozapine | クロザピン | NERVOUS SYSTEM | N05AH02 | X |  |
| Cobimetinib | - | ANTINEOPLASTIC AND IMMUNOMODULATING AGENTS | L01XE38 | - | - |
| Codeine | コデイン | RESPIRATORY SYSTEM | R05DA04 | CYP2D6 | Precautions regarding patients with specific backgrounds, Other notes |
| Crizanlizumab-tmca | - | BLOOD AND BLOOD FORMING ORGANS | B06AX01 | - | - |
| Crizotinib | クリゾチニブ | ANTINEOPLASTIC AND IMMUNOMODULATING AGENTS | L01XE16 | ALK, ROS1 | Efficacy or effect, Precautions related to efficacy or effect, Clinical results |
| Dabrafenib | ダブラフェニブ | ANTINEOPLASTIC AND IMMUNOMODULATING AGENTS | L01XE23 | BRAF, RAS | Efficacy or effect, Precautions related to efficacy or effect, Other notes, Clinical results |
| Daclatasvir | ダクラタスビル | ANTIINFECTIVES FOR SYSTEMIC USE | J05AP07 | X |  |
| Dacomitinib | ダコミチニブ | ANTINEOPLASTIC AND IMMUNOMODULATING AGENTS | L01XE47 | EGFR, L858R | Efficacy or effect, Precautions related to efficacy or effect, Side effects, Clinical results |
| Dapsone | ジアフェニルスルホン | ANTIINFECTIVES FOR SYSTEMIC USE | J04BA02 | G6PD | Precautions regarding patients with specific backgrounds |
| Darifenacin | - | GENITO URINARY SYSTEM AND SEX HORMONES | G04BD10 | - | - |
| Darunavir | ダルナビル | ANTIINFECTIVES FOR SYSTEMIC USE | J05AE10 | X |  |
| Dasabuvir, Ombitasvir, Paritaprevir, and Ritonavir | - | ANTIINFECTIVES FOR SYSTEMIC USE | J05AP52 | - | - |
| Dasatinib | ダサチニブ | ANTINEOPLASTIC AND IMMUNOMODULATING AGENTS | L01XE06 | BCR-ABL (Philadelphia chromosome) | Efficacy or effect, Precautions related to efficacy or effect, Dosage and administration |
| Denileukin Diftitox | デニロイキン　ジフチトクス | ANTINEOPLASTIC AND IMMUNOMODULATING AGENTS | L01XX29 | X |  |
| Desflurane | デスフルラン | NERVOUS SYSTEM | N01AB07 | X |  |
| Desipramine | - | NERVOUS SYSTEM | N06AA01 | - | - |
| Desvenlafaxine | - | NERVOUS SYSTEM | N06AX23 | - | - |
| Deutetrabenazine | - | NERVOUS SYSTEM | - | - | - |
| Dexlansoprazole | - | ALIMENTARY TRACT AND METABOLISM\ | A02BC06 | - | - |
| Dextromethorphan and Quinidine | - | NERVOUS SYSTEM | N07XX59 | - | - |
| Diazepam | ジアゼパム | NERVOUS SYSTEM | N05BA01 | X |  |
| Dinutuximab | ジヌツキシマブ | ANTINEOPLASTIC AND IMMUNOMODULATING AGENTS | L01XC16 | X | - |
| Divalproex sodium | - | NERVOUS SYSTEM | N03AG01 | - | - |
| Docetaxel | ドセタキセル | ANTINEOPLASTIC AND IMMUNOMODULATING AGENTS | L01CD02 | X |  |
| Dolutegravir | ドルテグラビル | ANTIINFECTIVES FOR SYSTEMIC USE | J05AX12 | X |  |
| Donepezil | ドネペジル | NERVOUS SYSTEM | N06DA02 | X |  |
| Dostarlimab-gxly | - | ANTINEOPLASTIC AND IMMUNOMODULATING AGENTS | L01XC40 | - | - |
| Doxepin | - | NERVOUS SYSTEM | N06AA12 | - | - |
| Dronabinol | - | ALIMENTARY TRACT AND METABOLISM\ | A04AD10 | - | - |
| Drospirenone and Ethinyl Estradiol | ドロスピレノン・エチニルエストラジオール　ベータデクス | GENITO URINARY SYSTEM AND SEX HORMONES | G03AA12 | X |  |
| Duloxetine | デュロキセチン | NERVOUS SYSTEM | N06AX21 | X |  |
| Durvalumab | デュルバルマブ | ANTINEOPLASTIC AND IMMUNOMODULATING AGENTS | L01XC28 | X |  |
| Duvelisib | - | ANTINEOPLASTIC AND IMMUNOMODULATING AGENTS | L01EM04 | - | - |
| Eculizumab | エクリズマブ | ANTINEOPLASTIC AND IMMUNOMODULATING AGENTS | L04AA25 | Anti-acetylcholine receptor antibody, anti-aquaporin 4 antibody | Precautions related to efficacy or effect |
| Efavirenz | エファビレンツ | ANTIINFECTIVES FOR SYSTEMIC USE | J05AG03 | CYP2B6 | Pharmacokinetics |
| Elagolix | - | SYSTEMIC HORMONAL PREPARATIONS, EXCL. SEX HORMONES AND INSULINS | H01CC03 | - | - |
| Elbasvir and Grazoprevir | - | ANTIINFECTIVES FOR SYSTEMIC USE | J05AP54 | - | - |
| Elexacaftor, Ivacaftor, and Tezacaftor | - | RESPIRATORY SYSTEM | R07AX32 | - | - |
| Eliglustat | エリグルスタット | ALIMENTARY TRACT AND METABOLISM\ | A16AX10 | CYP2D6 | Contraindications, Dosage and administration, Precautions related to usage and dosage, Precautions for use, Important basic notes, Interaction, Pharmacokinetics, Clinical results |
| Elosulfase | エロスルファーゼ | ALIMENTARY TRACT AND METABOLISM\ | A16AB12 | GALNS | Pharmacology |
| Eltrombopag | エルトロンボパグ | BLOOD AND BLOOD FORMING ORGANS | B02BX05 | Antithrombin III | Precautions for use |
| Emapalumab-lzsg | - | ANTINEOPLASTIC AND IMMUNOMODULATING AGENTS | L04AA39 | - | - |
| Enasidenib | - | ANTINEOPLASTIC AND IMMUNOMODULATING AGENTS | L01XX59 | - | - |
| Encorafenib | エンコラフェニブ | ANTINEOPLASTIC AND IMMUNOMODULATING AGENTS | L01EC03 | BRAF | Efficacy or effect, Precautions related to efficacy or effect, Usage and dosage, Precautions related to usage and dosage, Pharmacokinetics, Clinical results |
| Enflurane | - | ANTINEOPLASTIC AND IMMUNOMODULATING AGENTS | N01AB04 | - | - |
| Entrectinib | エヌトレクチニブ | ANTINEOPLASTIC AND IMMUNOMODULATING AGENTS | L01EX14 | NTRK, ROS1 | Efficacy or effect, Precautions related to efficacy or effect, Usage and dosage, Precautions regarding patients with specific backgrounds, Clinical results |
| Enzalutamide | エンザルタミド | ANTINEOPLASTIC AND IMMUNOMODULATING AGENTS | L02BB | X |  |
| Erdafitinib | - | ANTINEOPLASTIC AND IMMUNOMODULATING AGENTS | L01EX16 | - | - |
| Eribulin | エリブリン | ANTINEOPLASTIC AND IMMUNOMODULATING AGENTS | L01XX41 | X |  |
| Erlotinib | エルロチニブ | ANTINEOPLASTIC AND IMMUNOMODULATING AGENTS | L01XE03 | EGFR | Efficacy or effect, Clinical results |
| Erythromycin and Sulfisoxazole | - | ANTIINFECTIVES FOR SYSTEMIC USE | J01FA01 | - | - |
| Escitalopram | エスシタロプラム | NERVOUS SYSTEM | N06AB10 | CYP2C19, CYP2D6 | Precautions related to usage and dosage, Pharmacokinetics |
| Esomeprazole | エソメプラゾール | ALIMENTARY TRACT AND METABOLISM\ | A02BC05 | CYP2C19 | Pharmacokinetics |
| Estradiol and Progesterone | - | GENITO URINARY SYSTEM AND SEX HORMONES | G03FB06 | - | - |
| Eteplirsen | - | MUSCULO-SKELETAL SYSTEM | M09AX06 | - | - |
| Ethinyl estradiol and Norelgestromin | - | GENITO URINARY SYSTEM AND SEX HORMONES | G03AA13 | - | - |
| Everolimus | エベロリムス | ANTINEOPLASTIC AND IMMUNOMODULATING AGENTS | L01XE10 | Estrogen receptor, HER-2 | Precautions related to efficacy or effect, Side effect, Clinical result |
| Evinacumab-dgnb | - | ALIMENTARY TRACT AND METABOLISM | - | - | - |
| Evolocumab | エボロクマブ | CARDIOVASCULAR SYSTEM | C10AX13 | Familial hypercholesterolemia Heterozygotes, Familial hypercholesterolemia homozygotes | Dosage and administration, Side effects, Other notes, Clinical results |
| Exemestane | エキセメスタン | ANTINEOPLASTIC AND IMMUNOMODULATING AGENTS | L02BG06 | X |  |
| Fampridine | - | NERVOUS SYSTEM | N07XX07 | - | - |
| Fam-Trastuzumab Deruxtecan-nxki | トラスツズマブ　デルクステカン | ANTINEOPLASTIC AND IMMUNOMODULATING AGENTS | L01XC03 | HER2 | Efficacy or effect, Precautions related to efficacy or effect, Usage and dosage, Pharmacokinetics, Clinical result |
| Fesoterodine | フェソテロジン | GENITO URINARY SYSTEM AND SEX HORMONES | G04BD11 | CYP2D6 | Precautions regarding patients with specific backgrounds, Pharmacokinetics |
| Flibanserin | - | GENITO URINARY SYSTEM AND SEX HORMONES | G02CX02 | - | - |
| Fluorouracil | フルオロウラシル | ANTINEOPLASTIC AND IMMUNOMODULATING AGENTS | L01BC02 | DPD | Other notes |
| Fluoxetine | - | NERVOUS SYSTEM | N06AB03 | - | - |
| Flurbiprofen | フルルビプロフェン | MUSCULO-SKELETAL SYSTEM | M02AA19 | X |  |
| Flutamide | フルタミド | ANTINEOPLASTIC AND IMMUNOMODULATING AGENTS | L02BB01 | X |  |
| Fluvoxamine | フルボキサミン | NERVOUS SYSTEM | N06AB08 | X |  |
| Formoterol | ホルモテロール | RESPIRATORY SYSTEM | R03CC | X |  |
| Fosphenytoin | ホスフェニトイン | NERVOUS SYSTEM | N03AB05 | X |  |
| Fulvestrant | フルベストラント | ANTINEOPLASTIC AND IMMUNOMODULATING AGENTS | L02BA03 | Estrogen receptor, HER2 | Precautions related to efficacy / effect, Clinical results |
| Galantamine | ガランタミン | NERVOUS SYSTEM | N06DA04 | X |  |
| Gefitinib | ゲフィチニブ | ANTINEOPLASTIC AND IMMUNOMODULATING AGENTS | L01XE02 | EGFR | Efficacy or effect, Precautions related to efficacy or effect |
| Gemtuzumab Ozogamicin | ゲムツズマブオゾガマイシン | ANTINEOPLASTIC AND IMMUNOMODULATING AGENTS | L01XC05 | CD33 | Efficacy or effect, Precautions related to efficacy or effect, Pharmacokinetics, Clinical results |
| Gilteritinib | ギルテリチニブ | ANTINEOPLASTIC AND IMMUNOMODULATING AGENTS | L01EX13 | FLT3 | Efficacy or effect, Precautions related to efficacy or effect, Clinical results |
| Givosiran | ギボシラン | ALIMENTARY TRACT AND METABOLISM\ | A16AX16 | X | - |
| Glimepiride | グリメピリド | ALIMENTARY TRACT AND METABOLISM | A10BB12 | X |  |
| Glipizide | - | ALIMENTARY TRACT AND METABOLISM | A10BB07 | - | - |
| Glyburide (Glibenclamide) | グリベンクラミド | ALIMENTARY TRACT AND METABOLISM | A10BB01 | X |  |
| Golodirsen | - | MUSCULO-SKELETAL SYSTEM | M09AX08 | - | - |
| Goserelin | ゴセレリン | ANTINEOPLASTIC AND IMMUNOMODULATING AGENTS | L02AE03 | X |  |
| Hydralazine | ヒドララジン | CARDIOVASCULAR SYSTEM | C02DB02 | X |  |
| Hydroxychloroquine | ヒドロキシクロロキン | ANTIPARASITIC PRODUCTS, INSECTICIDES AND REPELLENTS | P01BA02 | G6PD | Precautions for use |
| Ibritumomab | イブリツモマブ | VARIOUS | V10XX02 | CD20 | Efficacy or effect, Clinical results |
| Ibrutinib | イブルチニブ | ANTINEOPLASTIC AND IMMUNOMODULATING AGENTS | L01XE27 | X |  |
| Iloperidone | - | NERVOUS SYSTEM | N05AX14 | - | - |
| Imatinib | イマチニブ | ANTINEOPLASTIC AND IMMUNOMODULATING AGENTS | L01XE01 | KIT (CD117), Philadelphia chromosome, FIP1L1-PDGFRα | Efficacy or Effect, Precautions related to efficacy or effect, Usage and dosage, Important basic notes, Clinical results |
| Imipramine | イミプラミン | NERVOUS SYSTEM | N06AA02 | X |  |
| Indacaterol | インダカテロール | RESPIRATORY SYSTEM | R03AC18 | UGT1A1 | Pharmacokinetics |
| Inebilizumab-cdon | イネビリズマブ | ANTINEOPLASTIC AND IMMUNOMODULATING AGENTS | L04AA47 | AQP4 | Precautions related to efficacy or effect, Clinical results |
| Infigratinib | - | ANTINEOPLASTIC AND IMMUNOMODULATING AGENTS | - | - | - |
| Inotersen | - | NERVOUS SYSTEM | N07XX15 | - | - |
| Inotuzumab Ozogamicin | イノツズマブオゾガマイシン | ANTINEOPLASTIC AND IMMUNOMODULATING AGENTS | L01XC26 | CD22, Philadelphia chromosome | Efficacy or effect, Precautions related to efficacy or effect, Side effects, Pharmacokinetics, Clinical results |
| Ipilimumab | イピリムマブ | ANTINEOPLASTIC AND IMMUNOMODULATING AGENTS | L01XC11 | Microsatellite instability, Mismatch Repair, PD-L1, EGFR, ALK, HLA-A2* 0201 | Efficacy or effect, Precautions related to efficacy or effect, Dosage and administration, Precautions related to usage and dosage, Clinical results |
| Irinotecan | イリノテカン | ANTINEOPLASTIC AND IMMUNOMODULATING AGENTS | L01XX19 | UGT1A1 | Precautions related to efficacy or effect, Important basic notes, Pharmacokinetics, Clinical results |
| Isatuximab- irfc | イサツキシマブ | ANTINEOPLASTIC AND IMMUNOMODULATING AGENTS | L01XC38 | X |  |
| Isoflurane | イソフルラン | NERVOUS SYSTEM | N01AB06 | X |  |
| Isoniazid | イソニアジド | ANTIINFECTIVES FOR SYSTEMIC USE | J04AC01 | NAT2 | Pharmacokinetics |
| Isoniazid, Pyrazinamide, and Rifampin | - | ANTIINFECTIVES FOR SYSTEMIC USE | J04AM05 | - | - |
| Isosorbide Dinitrate | 硝酸イソソルビド | CARDIOVASCULAR SYSTEM | C01DA08 | X |  |
| Isosorbide Mononitrate | 一硝酸イソソルビド | CARDIOVASCULAR SYSTEM | C01DA14 | X |  |
| Ivacaftor | - | RESPIRATORY SYSTEM | R07AX02 | - | - |
| Ivacaftor and Lumacaftor | - | RESPIRATORY SYSTEM | R07AX30 | - | - |
| Ivacaftor and Tezacaftor | - | RESPIRATORY SYSTEM | R07AX31 | - | - |
| Ivosidenib | - | ANTINEOPLASTIC AND IMMUNOMODULATING AGENTS | L01XX62 | - | - |
| Ixabepilone | - | ANTINEOPLASTIC AND IMMUNOMODULATING AGENTS | L01DC04 | - | - |
| Lacosamide | ラコサミド | NERVOUS SYSTEM | N03AX18 | CYP2C19 | Pharmacokinetics |
| Lansoprazole | ランソプラゾール | ALIMENTARY TRACT AND METABOLISM\ | A02BC03 | X |  |
| Lapatinib | ラパチニブ | ANTINEOPLASTIC AND IMMUNOMODULATING AGENTS | L01XE07 | HER2 / hormone receptor, HLA | Effective or Efficacy, Precautions related to efficacy or effect, Other notes, Clinical results, |
| Larotrectinib | ラロトレクチニブ硫酸塩 | ANTINEOPLASTIC AND IMMUNOMODULATING AGENTS | L01EX12 | NTRK | Efficacy or effect, Precautions related to efficacy or effect, Precautions regarding patients with specific backgrounds, Clinical results |
| Ledipasvir and Sofosbuvir | レジパスビル　アセトン付加物・ソホスブビル | ANTIINFECTIVES FOR SYSTEMIC USE | J05AP51 | X |  |
| Lenalidomide | レナリドミド | ANTINEOPLASTIC AND IMMUNOMODULATING AGENTS | L04AX04 | long arm of chromosome 5 | Efficacy or effect, Precautions related to efficacy or effect, Usage and dosage, Precautions related to usage and dosage, Pharmacokinetics, Clinical results |
| Lenvatinib | レンバチニブ | ANTINEOPLASTIC AND IMMUNOMODULATING AGENTS | L01XE29 | X |  |
| Lesinurad | - | MUSCULO-SKELETAL SYSTEM | M04AB05 | - | - |
| Letrozole | レトロゾール | ANTINEOPLASTIC AND IMMUNOMODULATING AGENTS | L02BG04 | CYP2A6 | pharmacokinetics |
| Lidocaine and Prilocaine | リドカイン・プロピトカイン配合剤 | NERVOUS SYSTEM | N01BB | G6PD | Precautions for use |
| Lidocaine and Tetracaine | - | NERVOUS SYSTEM | N01BB52 | - | - |
| Lofexidine | - | NERVOUS SYSTEM | N07BC04 | - | - |
| Lomitapide | ロミタピド | CARDIOVASCULAR SYSTEM | C10AX12 | Homozygous familial hypercholesterolemia | Efficacy or effect, Clinical results |
| Lonafarnib | - | ALIMENTARY TRACT AND METABOLISM | - | - | - |
| Lorlatinib | ロルラチニブ | ANTINEOPLASTIC AND IMMUNOMODULATING AGENTS | L01ED05 | ALK, ROS1 | Effective or Efficacy, Pharmacokinetics, Clinical results |
| Lumasiran | - | ALIMENTARY TRACT AND METABOLISM | A16AX18 | - | - |
| Luspatercept–aamt | - | BLOOD AND BLOOD FORMING ORGANS | B03XA06 | - | - |
| Lusutrombopag | ルストロンボパグ | BLOOD AND BLOOD FORMING ORGANS | B02BX07 | X |  |
| Lutetium Dotatate Lu-177 | ルテチウムオキソドトレオチド（１７７Ｌｕ） | VARIOUS | V10XX04 | X | - |
| Mafenide | - | DERMATOLOGICALS | D06BA03 | - | - |
| Maraviroc | マラビロク | ANTIINFECTIVES FOR SYSTEMIC USE | J05AX09 | CCR5 | Efficacy or effect, Precautions related to efficacy or effect, Pharmacology |
| Margetuximab-cmkb | - | ANTINEOPLASTIC AND IMMUNOMODULATING AGENTS | - | - | - |
| Meclizine | - | RESPIRATORY SYSTEM | R06AE05 | - | - |
| Meloxicam | メロキシカム | MUSCULO-SKELETAL SYSTEM | M01AC06 | X |  |
| Mepivacaine | メピバカイン | NERVOUS SYSTEM | N01BB03 | X |  |
| Mercaptopurine | メルカプトプリン | ANTINEOPLASTIC AND IMMUNOMODULATING AGENTS | L01BB02 | NUDT15 | Other notes |
| Methylene Blue | メチルチオニニウム塩化物水和物 | VARIOUS | V03AB | G6PD, NADPH reductase | Contraindications, Important basic notes |
| Metoclopramide | メトクロプラミド | ALIMENTARY TRACT AND METABOLISM\ | A03FA01 | X |  |
| Metoprolol (Metoprolol tartrate) | メトプロロール | CARDIOVASCULAR SYSTEM | C07AB02 | X |  |
| Metreleptin | メトレレプチン | ALIMENTARY TRACT AND METABOLISM | A16AA07 | X | X |
| Midostaurin | - | ANTINEOPLASTIC AND IMMUNOMODULATING AGENTS | L01XE39 | - | - |
| Migalastat | ミガーラスタット塩酸塩 | ALIMENTARY TRACT AND METABOLISM\ | A16AX14 | GLA | Efficacy or effect, Precautions related to efficacy or effect, Clinical results |
| Mirabegron | ミラベグロン | GENITO URINARY SYSTEM AND SEX HORMONES | G04BD12 | X |  |
| Mivacurium | - | MUSCULO-SKELETAL SYSTEM | M03AC10 | - | - |
| Modafinil | モダフィニル | NERVOUS SYSTEM | N06BA07 | X |  |
| Moviprep |  | Various | - | G6PD | Precautions for use |
| Mycophenolic Acid | ミコフェノール酸 | ANTINEOPLASTIC AND IMMUNOMODULATING AGENTS | L04AA06 | HGPRT | Important basic notes |
| Nalidixic Acid |  | ANTIINFECTIVES FOR SYSTEMIC USE | J01MB02 | G6PD | Side effects |
| Nebivolol | - | CARDIOVASCULAR SYSTEM | C07AB12 | - | - |
| Nefazodone | - | NERVOUS SYSTEM | N06AX06 | - | - |
| Neratinib | - | ANTINEOPLASTIC AND IMMUNOMODULATING AGENTS | L01EH02 | - | - |
| Nilotinib | ニロチニブ | ANTINEOPLASTIC AND IMMUNOMODULATING AGENTS | L01XE08 | BCR-ABL | Precautions related to efficacy or effect, pharmacology |
| Niraparib | ニラパリブ | ANTINEOPLASTIC AND IMMUNOMODULATING AGENTS | L01XX54 | homologous recombination repair deficiency, BCRA | Efficacy or effect, Precautions related to efficacy or effect, Clinical results |
| Nitrofurantoin | - | ANTIINFECTIVES FOR SYSTEMIC USE | J01XE01 | - | - |
| Nivolumab | ニボルマブ | ANTINEOPLASTIC AND IMMUNOMODULATING AGENTS | L01XC17 | microsatellite instability (MSI), EGFR, ALK, PD-L1, BRAF (Clinical results), mismatch repair (Clinical results) | Efficacy or effect, Precautions related to efficacy or effect, Usage and dosage, Precautions related to usage and dosage, Clinical results |
| Norfloxacin | ノルフロキサシン | ANTIINFECTIVES FOR SYSTEMIC USE | J01MA06 | X |  |
| Nortriptyline | ノルトリプチリン | NERVOUS SYSTEM | N06AA10 | X |  |
| Nusinersen | ヌシネルセン | MUSCULO-SKELETAL SYSTEM | M09AX07 | SMN2 | Precautions related to efficacy or effect, Clinical results |
| Obinutuzumab | オビヌツズマブ | ANTINEOPLASTIC AND IMMUNOMODULATING AGENTS | L01XC15 | CD20 | Effective or Efficacy, Precautions related to efficacy or effect, Pharmacokinetics, Clinical results |
| Ofatumumab | オファツムマブ | Antineoplastic and immunomodulating agents | L01XC10 | CD20 | Efficacy or effect, Precautions related to efficacy or effect |
| Olaparib | オラパリブ | ANTINEOPLASTIC AND IMMUNOMODULATING AGENTS | L01XX46 | BRCA, HER2, homologous recombination repair | Efficacy or effect, Precautions related to efficacy or effect, Usage and dosage, Precautions related to usage and dosage, Clinical results |
| Olaratumab | - | ANTINEOPLASTIC AND IMMUNOMODULATING AGENTS | L01XC27 | - | - |
| Oliceridine | - | NERVOUS SYSTEM | - | - | - |
| Omacetaxine | - | ANTINEOPLASTIC AND IMMUNOMODULATING AGENTS | L01XX40 | - | - |
| Ombitasvir, Paritaprevir, and Ritonavir |  | ANTIINFECTIVES FOR SYSTEMIC USE | J05AP53 | - | - |
| Omeprazole | オメプラゾール | ALIMENTARY TRACT AND METABOLISM\ | A02BC01 | CYP2C19 | Pharmacokinetics |
| Ondansetron | オンダンセトロン | ALIMENTARY TRACT AND METABOLISM\ | A04AA01 | X |  |
| Osimertinib | オシメルチニブ | ANTINEOPLASTIC AND IMMUNOMODULATING AGENTS | L01XE35 | EGFR | Effective or Efficacy, Precautions related to efficacy or effect, Clinical results |
| Ospemifene | - | GENITO URINARY SYSTEM AND SEX HORMONES | G03XC05 | - | - |
| Oxcarbazepine | オクスカルバゼピン | NERVOUS SYSTEM | N03AF02 | - | - |
| Oxymetazoline and Tetracaine | - | RESPIRATORY SYSTEM | R01AA05 | - | - |
| Palbociclib | パルボシクリブ | ANTINEOPLASTIC AND IMMUNOMODULATING AGENTS | L01XE33 | Hormone receptor (HR), HER2 | Effective or Efficacy, Side effects, Clinical results |
| Paliperidone | パリペリドン | NERVOUS SYSTEM | N05AX13 | X |  |
| Palonosetron | パロノセトロン | ALIMENTARY TRACT AND METABOLISM\ | A04AA05 | CYP2D6 | Pharmacokinetics |
| Panitumumab | パニツムマブ | ANTINEOPLASTIC AND IMMUNOMODULATING AGENTS | L01XC08 | RAS | Effective or Efficacy, Precautions related to efficacy or effect, Side effects, Clinical results |
| Pantoprazole | - | ALIMENTARY TRACT AND METABOLISM\ | A02BC02 | - | - |
| Parathyroid Hormone | - | SYSTEMIC HORMONAL PREPARATIONS, EXCL. SEX HORMONES AND INSULINS | H05AA03 | - | - |
| Paroxetine | パロキセチン | NERVOUS SYSTEM | N06AB05 | CYP2D6 | Pharmacokinetics |
| Patisiran | パチシランナトリウム | NERVOUS SYSTEM | N07XX12 | Transthyretin (TTR) | Efficacy or effect, Precautions related to efficacy or effect, Important basic notes, Pharmacokinetics, Clinical results |
| Pazopanib | パゾパニブ | ANTINEOPLASTIC AND IMMUNOMODULATING AGENTS | L01XE11 | X |  |
| Peginterferon Alfa-2b | ペグインターフェロン　アルファ－２b | ANTINEOPLASTIC AND IMMUNOMODULATING AGENTS | L03AB10 | X |  |
| Pegloticase | - | MUSCULO-SKELETAL SYSTEM | M04AX02 | - | - |
| Pembrolizumab | ペムブロリズマブ | ANTINEOPLASTIC AND IMMUNOMODULATING AGENTS | L01XC18 | microsatellite instability, PD-L1, ALK, EGFR | Efficacy or effect, Precautions related to efficacy or effect, Usage and dosage, Clinical results |
| Pemigatinib | ペミガチニブ | ANTINEOPLASTIC AND IMMUNOMODULATING AGENTS | L01EX20 | FGFR2 | Efficacy or effect, Precautions related to efficacy or effect, Clinical results |
| Perphenazine | ペルフェナジン | NERVOUS SYSTEM | N05AB03 | X |  |
| Pertuzumab | ペルツズマブ | ANTINEOPLASTIC AND IMMUNOMODULATING AGENTS | L01XC13 | HER2 | Effective or Efficacy, Precautions related to efficacy or effect, Pharmacokinetics, Clinical results |
| Phenytoin | フェニトイン | NERVOUS SYSTEM | N03AB02 | X |  |
| Pimozide | ピモジド | NERVOUS SYSTEM | N05AG02 | X |  |
| Piroxicam | ピロキシカム | MUSCULO-SKELETAL SYSTEM | M01AC01 | X |  |
| Pitolisant | - | NERVOUS SYSTEM | N07XX11 | - | - |
| Ponatinib | ポナチニブ | ANTINEOPLASTIC AND IMMUNOMODULATING AGENTS | L01XE24 | BCR-ABL (Philadelphia chromosome) | Efficacy or effect, Precautions related to efficacy or effect |
| Pralsetinib | - | ANTINEOPLASTIC AND IMMUNOMODULATING AGENTS | - | - | - |
| Prasugrel | プラスグレル | BLOOD AND BLOOD FORMING ORGANS | B01AC22 | X |  |
| Primaquine | プリマキン | ANTIPARASITIC PRODUCTS, INSECTICIDES AND REPELLENTS | P01BA03 | G6PD, NADH / methemoglobin reductase | Warning, Contraindications, Precautions for use |
| Probenecid | プロベネシド | MUSCULO-SKELETAL SYSTEM | M04AB01 | X |  |
| Procainamide | プロカインアミド | CARDIOVASCULAR SYSTEM | C01BA02 | X |  |
| Propafenone | プロパフェノン | CARDIOVASCULAR SYSTEM | C01BC03 | X |  |
| Propranolol | プロプラノロール | CARDIOVASCULAR SYSTEM | C07AA05 | X |  |
| Protriptyline | - | NERVOUS SYSTEM | N06AA11 | - | - |
| Quinidine | キニジン | CARDIOVASCULAR SYSTEM | C01BA01 | X |  |
| Quinine Sulfate | キニーネ塩酸塩水和物 | ANTIPARASITIC PRODUCTS, INSECTICIDES AND REPELLENTS | P01BC01 | X |  |
| Rabeprazole | ラベプラゾール | ALIMENTARY TRACT AND METABOLISM\ | A02BC04 | CYP2C19 | pharmacokinetics, pharmacology |
| Raloxifene | ラロキシフェン | GENITO URINARY SYSTEM AND SEX HORMONES | G03XC01 | X |  |
| Raltegravir | ラルテグラビル | ANTIINFECTIVES FOR SYSTEMIC USE | J05AX08 | X |  |
| Ramucirumab | ラムシルマブ | ANTINEOPLASTIC AND IMMUNOMODULATING AGENTS | L01XC21 | EGFR | Precautions related to efficacy or effect, Usage and dosage, Clinical results |
| Ranolazine | - | CARDIOVASCULAR SYSTEM | C01EB18 | - | - |
| Rasburicase | ラスブリカーゼ | VARIOUS | V03AF07 | G6PD | Warning, Contraindications |
| Regorafenib | レゴラフェニブ | ANTINEOPLASTIC AND IMMUNOMODULATING AGENTS | L01XE21 | KRAS | Clinical results |
| Ribociclib | - | ANTINEOPLASTIC AND IMMUNOMODULATING AGENTS | L01XE42 | - | - |
| Rimegepant | - | NERVOUS SYSTEM | - | - | - |
| Risdiplam | リスジプラム | MUSCULO-SKELETAL SYSTEM | M09AX10 | SMN1, SMN2 | Precautions related to efficacy or effect |
| Risperidone | リスペリドン | NERVOUS SYSTEM | N05AX08 | X |  |
| Rituximab | リツキシマブ | ANTINEOPLASTIC AND IMMUNOMODULATING AGENTS | L01XC02 | CD20 | Efficacy or effect, Precautions related to efficacy or effect |
| Rivaroxaban | リバーロキサバン | BLOOD AND BLOOD FORMING ORGANS | B01AF01 | X |  |
| Ropivacaine | ロピバカイン | NERVOUS SYSTEM | N01BB09 | X |  |
| Rosuvastatin | ロスバスタチン | CARDIOVASCULAR SYSTEM | C10AA07 | X |  |
| Rucaparib | - | ANTINEOPLASTIC AND IMMUNOMODULATING AGENTS | L01XK03 | - | - |
| Ruxolitinib | ルキソリチニブ | ANTINEOPLASTIC AND IMMUNOMODULATING AGENTS | L01XE18 | JAK2 | Pharmacology |
| Sacituzumab Govitecan-hziy | - | ANTINEOPLASTIC AND IMMUNOMODULATING AGENTS | L01 | - | - |
| Satralizumab-mwge | サトラリズマブ | ANTINEOPLASTIC AND IMMUNOMODULATING AGENTS | L04AC19 | Anti-aquaporin 4 antibody | Precautions related to efficacy or effect |
| Selpercatinib | - | ANTINEOPLASTIC AND IMMUNOMODULATING AGENTS | L01 | RET | Efficacy or effect, Precautions related to efficacy or effect, Clinical results |
| Setmelanotide | - | ALIMENTARY TRACT AND METABOLISM | - | - | - |
| Sevoflurane | セボフルラン | NERVOUS SYSTEM | N01AB08 | X |  |
| Sildenafil | シルデナフィル | GENITO URINARY SYSTEM AND SEX HORMONES | G04BE03 | X |  |
| Simeprevir | シメプレビル | ANTIINFECTIVES FOR SYSTEMIC USE | J05AP05 | - | - |
| Siponimod | シポニモド　フマル酸 | ANTINEOPLASTIC AND IMMUNOMODULATING AGENTS | L04AA42 | CYP2C9 | Contraindications, Precautions related to usage and dosage, Precautions regarding patients with specific backgrounds, Other notes, Pharmacokinetics |
| Sodium Nitrite | - | VARIOUS | V03AB08 | - | - |
| Sodium Oxybate | - | NERVOUS SYSTEM | N01AX11 | - | - |
| Sodium Phenylbutyrate | フェニル酪酸ナトリウム | ALIMENTARY TRACT AND METABOLISM\ | A16AX03 | X |  |
| Sofosbuvir | ソホスブビル | ANTIINFECTIVES FOR SYSTEMIC USE | J05AP08 | X |  |
| Sofosbuvir and Velpatasvir | ソホスブビル・ベルパタスビル | ANTIINFECTIVES FOR SYSTEMIC USE | J05AP55 | X |  |
| Sofosbuvir, Velpatasvir, and Voxilaprevir | - | ANTIINFECTIVES FOR SYSTEMIC USE | J05AP56 | - | - |
| Sotorasib | - | ANTINEOPLASTIC AND IMMUNOMODULATING AGENTS | - | - | - |
| Succimer | - | VARIOUS | V09CA02 | - | - |
| Succinylcholine | スキサメトニウム塩化物水和物 | MUSCULO-SKELETAL SYSTEM | M03AB01 | X |  |
| Sulfadiazine | スルファジアジン銀 | DERMATOLOGICALS | D06BA01 | G6PD | Precautions for use |
| Sulfamethoxazole and Trimethoprim | スルファメトキサゾール・トリメトプリム | ANTIINFECTIVES FOR SYSTEMIC USE | J01EE01 | G6PD | Contraindications |
| Sulfasalazine | サラゾスルファピリジン | ALIMENTARY TRACT AND METABOLISM\ | A07EC01 | G6PD | Precautions for use |
| Synthetic Conjugated Estrogens, A | - | GENITO URINARY SYSTEM AND SEX HORMONES | - | - | - |
| Tafamidis | タファミジスメグルミン | NERVOUS SYSTEM | N07XX08 | Transthyretin-type (TTR) | Efficacy or effect, Precautions related to efficacy or effect, Usage and dosage, Clinical results |
| Tafenoquine | - | ANTIINFECTIVES FOR SYSTEMIC USE | P01BA07 | - | - |
| Talazoparib | - | ANTINEOPLASTIC AND IMMUNOMODULATING AGENTS | L01XK04 | - | - |
| Tamoxifen | タモキシフェン | ANTINEOPLASTIC AND IMMUNOMODULATING AGENTS | L02BA01 | X |  |
| Tamsulosin | タムスロシン | GENITO URINARY SYSTEM AND SEX HORMONES | G04CA02 | X |  |
| Telaprevir | テラプレビル | ANTIINFECTIVES FOR SYSTEMIC USE | J05AP02 | - | - |
| Tepotinib | テポチニブ | ANTINEOPLASTIC AND IMMUNOMODULATING AGENTS | L01EX21 | MET gene exon 14 | Efficacy or effect, Precautions related to efficacy or effect |
| Tetrabenazine | テトラベナジン | NERVOUS SYSTEM | N07XX06 | CYP2D6 | Precautions related to usage and dosage, Pharmacokinetics |
| Thioguanine | - | ANTINEOPLASTIC AND IMMUNOMODULATING AGENTS | L01BB03 | - | - |
| Thioridazine | - | NERVOUS SYSTEM | N05AC02 | - | - |
| Ticagrelor | チカグレロル | BLOOD AND BLOOD FORMING ORGANS | B01AC24 | X |  |
| Tipiracil and Trifluridine | トリフルリジン・チピラシル | ANTINEOPLASTIC AND IMMUNOMODULATING AGENTS | L01BC59 | HER2, KRAS | Clinical results |
| Tolazamide | - | ALIMENTARY TRACT AND METABOLISM | A10BB05 | - | - |
| Tolbutamide | - | ALIMENTARY TRACT AND METABOLISM | A10BB03 | - | - |
| Tolterodine | トルテロジン | GENITO URINARY SYSTEM AND SEX HORMONES | G04BD07 | CYP2D6 | Pharmacokinetic, Clinical results |
| Toremifene | トレミフェン | ANTINEOPLASTIC AND IMMUNOMODULATING AGENTS | L02BA02 | Estrogen receptor (ER) | Pharmacology |
| Tramadol | トラマドール | NERVOUS SYSTEM | N02AX02 | CYP2D6 | Other notes |
| Trametinib | トラメチニブ | ANTINEOPLASTIC AND IMMUNOMODULATING AGENTS | L01XE25 | BRAF | Efficacy or effect, Precautions related to efficacy or effect, Clinical results |
| Trastuzumab | トラスツズマブ | ANTINEOPLASTIC AND IMMUNOMODULATING AGENTS | L01XC03 | HER2 | Efficacy or effect, Precautions related to efficacy or effect, Usage and dosage, Precautions related to usage and dosage, Side effects, Pharmacokinetics, Clinical results |
| Tretinoin | トレチノイン | ANTINEOPLASTIC AND IMMUNOMODULATING AGENTS | L01XX14 | PML-RAR-α | Pharmacology |
| Triheptanoin | - | ALIMENTARY TRACT AND METABOLISM\ | A16AX17 | - | - |
| Trimipramine | トリミプラミン | NERVOUS SYSTEM | N06AA06 | X |  |
| Tucatinib | - | ANTINEOPLASTIC AND IMMUNOMODULATING AGENTS | L01EH03 | - | - |
| Umeclidinium | ウメクリジニウム | RESPIRATORY SYSTEM | R03AL03 | X |  |
| Upadacitinib | ウパダシチニブ | ANTINEOPLASTIC AND IMMUNOMODULATING AGENTS | L04AA44 | X |  |
| Ustekinumab | ウステキヌマブ | ANTINEOPLASTIC AND IMMUNOMODULATING AGENTS | L04AC05 | X |  |
| Valbenazine | - | NERVOUS SYSTEM | N07XX13 | - | - |
| Valproic Acid | バルプロ酸 | NERVOUS SYSTEM | N03AG01 | urea cycle disorder | Contraindications, Precautions regarding patients with specific backgrounds |
| Vandetanib | バンデタニブ | ANTINEOPLASTIC AND IMMUNOMODULATING AGENTS | L01XE12 | RET | Pharmacology |
| Velaglucerase alfa | ベラグルセラーゼ　アルファ | ALIMENTARY TRACT AND METABOLISM\ | A16AB10 | β-glucocerebrosidase | Efficacy or effect, Precautions related to efficacy or effect, Important basic notes, Pharmacology |
| Vemurafenib | ベムラフェニブ | ANTINEOPLASTIC AND IMMUNOMODULATING AGENTS | L01XE15 | BRAF / RAS | Efficacy or effect, Precautions related to efficacy or effect, Other notes, Clinical results |
| Venetoclax | ベネトクラクス | ANTINEOPLASTIC AND IMMUNOMODULATING AGENTS | L01XX52 | X |  |
| Venlafaxine | ベンラファキシン | NERVOUS SYSTEM | N06AX16 | CYP2D6 | Pharmacokinetic |
| Viloxazine | - | NERVOUS SYSTEM | N06AX09 | - | - |
| Viltolarsen | - | NERVOUS SYSTEM | - | - | - |
| Vincristine | ビンクリスチン | ANTINEOPLASTIC AND IMMUNOMODULATING AGENTS | L01CA02 | X |  |
| Vitamin C | ビタミンC | Alimentary tract and metabolism | A11GA01 | X |  |
| Voriconazole | ボリコナゾール | NERVOUS SYSTEM | J02AC03 | CYP2C19 | Pharmacokinetic |
| Vortioxetine | ボルチオキセチン | NERVOUS SYSTEM | N06AX26 | CYP2D6 | Precautions related to usage and dosage, Precautions regarding patients with specific backgrounds, Pharmacokinetic |
| Voxelotor | - | BLOOD AND BLOOD FORMING ORGANS | - | - | - |
| Warfarin | ワルファリ | BLOOD AND BLOOD FORMING ORGANS | B01AA03 | CYP2C9 | Pharmacokinetic |
